# A semi-empirical Bayes approach for calibrating weak instrumental bias in sex-specific Mendelian randomization studies

**DOI:** 10.1016/j.ajhg.2025.07.015

**Published:** 2025-09-04

**Authors:** Yu-Jyun Huang, Nuzulul Kurniansyah, Daniel F. Levey, Joel Gelernter, Jennifer E. Huffman, Kelly Cho, Peter W.F. Wilson, Daniel J. Gottlieb, Kenneth M. Rice, Tamar Sofer

**Affiliations:** 1 Department of Medicine, Harvard Medical School, Boston, MA, USA; 2 CardioVascular Institute (CVI), Beth Israel Deaconess Medical Center, Boston, MA, USA; 3 Department of Medicine, Brigham and Women’s Hospital, Boston, MA, USA; 4 Division of Human Genetics, Department of Psychiatry, Yale University School of Medicine, New Haven, CT, USA; 5 Department of Psychiatry, Veterans Affairs Connecticut Healthcare Center, West Haven, CT, USA; 6 Massachusetts Veterans Epidemiology Research and Information Center, VA Healthcare System, Boston, MA, USA; 7 VA Palo Alto Health Care System, Palo Alto, CA, USA; 8 Palo Alto Veterans Institute for Research, Palo Alto, CA, USA; 9 Division of Aging, Department of Medicine, Brigham and Women’s Hospital and Harvard Medical School, Boston, MA, USA; 10 Atlanta VA Healthcare System, Decatur, GA, USA; 11 Department of Biostatistics, University of Washington, Seattle, WA, USA; 12 Department of Biostatistics, Harvard T.H. Chan School of Public Health, Boston, MA, USA

## Abstract

Strong sex differences exist in sleep phenotypes and also cardiovascular diseases (CVDs). However, sex-specific causal effects of sleep phenotypes on CVD-related outcomes have not been thoroughly examined. Mendelian randomization (MR) analysis is a useful approach for estimating the causal effect of a risk factor on an outcome of interest when interventional studies are not available. We first conducted sex-specific genome-wide association studies (GWASs) for suboptimal-sleep phenotypes (insomnia, obstructive sleep apnea [OSA], short and long sleep durations, and excessive daytime sleepiness) utilizing the Million Veteran Program (MVP) dataset. We then developed a semi-empirical Bayesian framework that (1) calibrates variant-phenotype effect estimates by leveraging information across sex groups and (2) applies shrinkage sex-specific effect estimates in MR analysis to alleviate weak instrumental bias when sex groups are analyzed in isolation. Simulation studies demonstrate that the causal effect estimates derived from our framework are substantially more efficient than those obtained through conventional methods. We estimated the causal effects of sleep phenotypes on CVD-related outcomes using sex-specific GWAS data from the MVP and All of Us. Significant sex differences in causal effects were observed, particularly between OSA and chronic kidney disease, as well as long sleep duration on several CVD-related outcomes. By applying shrinkage estimates for instrumental variable selection, we identified multiple sex-specific significant causal relationships between OSA and CVD-related phenotypes. The method is generalizable and can be used to improve power and alleviate weak instrument bias when only a small sample is available for a specific condition or group.

## Introduction

Investigating sex differences in health and disease mechanisms is a leading public health research priority.^1–3^ Sex differences are evident in various health conditions, including suboptimal-sleep phenotypes and cardiovascular diseases (CVDs). For example, there is a higher prevalence of insomnia in women,^4,5^ whereas obstructive sleep apnea (OSA) is more common in men.^6,7^ Cardiovascular-related diseases, such as myocardial infarction and hypertension (HTN), generally present with a higher incidence in male adults compared to females.^8–10^ Increasing numbers of genome-wide association studies (GWASs), which, like other genomic studies, often analyze only autosomal chromosomes, have identified strong signals of sex differences.^11–13^ Examples include, but are not limited to, sex differences in genetic variant effect sizes,^14–16^ sex-specific genetic risk associations,^17–19^ and sex-biased gene/protein expression level.^20–22^ Sex-specific causal effects of modifiable risk exposures on outcomes can, under some conditions, be obtained via Mendelian randomization (MR) analysis.^23^ But because of the paucity of sex-specific interventional studies, or studies with sufficient sex-stratified sample sizes, much remains unknown about how sex-specific causal effects may inform targeted disease treatments or interventions, ultimately limiting efforts to reduce sex disparities in health.^24–26^

MR analysis is widely used in genetic epidemiology because it can, using GWAS summary statistics alone,^27–29^ estimate causal effects from observational data. Sex-specific MR analysis, however, is limited in comparison: not only is each sex group’s sample size smaller than the total in any one study, but GWAS reporting is not always sex specific. This problem can be worse where GWAS participation is biased for structural or other reasons. For example, in the Million Veteran Program (MVP) of the US Department of Veterans Affairs (VA) healthcare system, which collected genetic data and extensive phenotypes from US veterans, only 10% of participants are female, in line with the proportion of female veterans. In sex-specific MR analysis, the small sample size of the female MVP population particularly limits the strength of instrumental variables (IVs) identified from female-specific GWASs, which may make causal effect estimates unstable due to the corresponding weak IV bias.^30,31^

To address these challenges, we (1) performed sex-specific GWASs of sleep phenotypes in the MVP, (2) developed a novel statistical approach to enhance the precision of sex-specific variant-phenotype effect estimates by leveraging information across sex groups, and (3) integrated our new shrinkage estimator into MR analyses to improve causal effect estimates, particularly for the sex group with smaller sample sizes. Motivation for this approach comes from recent findings of the high correlation between the genetic components of females and males of multiple traits,^32,33^ suggesting that many (though not all) variant associations are similar between sexes. Thus, focusing on the MVP, where the female population is small, our approach is to borrow information from the male population in an adaptive manner to improve female-specific variant effect estimates and, ultimately, exposure-outcome causal effect estimates.

The proposed approach, incorporating the spirit of both transfer learning and empirical Bayes, uses male-specific effect size estimates to specify prior distributions on the female-specific variant-exposure effect sizes (i.e., using information from the larger sample to improve power in the smaller). The inverse variance-weighted meta-analysis estimator and the adaptive weight (AW) estimator (proposed for analyzing secondary outcomes in case-control studies^34–36^) can both be derived as the posterior mean in the proposed framework. In simulation studies, compared to the standard use of variant-exposure summary statistics in MR analysis, our approach improves the efficiency of exposure-outcome causal effect estimates. Finally, using sex-specific data from the MVP, along with genetic association results from the All of Us (AoU) study, we applied a two-sample MR approach to estimate the causal effects of sleep phenotypes on CVD-related outcomes. Our method identified several sex-specific causal associations. Specifically, insomnia was causally associated with an increased risk of chronic kidney disease (CKD) in females, long sleep was linked to a higher risk of HTN in females, and short sleep was associated with an increased risk of coronary artery disease (CAD) in males. A statistically significant sex difference in the causal effect of OSA on CKD was also identified. In addition, using shrinkage estimates for IV selection, we detected several statistically significant causal effects of OSA on CVD-related outcomes, as well as distinct sex differences in the causal patterns of long sleep on CVD-related outcomes, with higher risks observed in females.

## Methods

### Overview of semi-empirical Bayesian method for calibrating genetic variant effect size estimates utilizing information across groups

While our method is general, we focus on the need to improve the estimation of variant effect sizes in the relatively small MVP female population and do so by borrowing information from the male population. Another simplification we make in the exposition is to focus on the “exposure” GWAS, even though the same framework can be applied to any trait GWAS, regardless of its role in an MR analysis. Throughout this paper, we use γ to represent “variant-exposure” effect size and Γ to represent “variant-outcome” effect size. To motivate our method, we consider a Bayesian prior on $\gamma_{jF}$, the female-specific effect size of the *j*-th SNP on the sleep phenotype, specifically

(Equation 1) $$\gamma_{jF}\sim N\left(\theta_{jF},\tau_{jF}^{2}\right),$$

where $\theta_{jF}$ and $\tau_{jF}$ are the prior mean and standard deviation (SD), respectively. The approximate distribution of the “raw” female-specific effect size estimate (i.e., an estimate that relies on female data only) is given by

(Equation 2) $${\hat{\gamma }}_{jF,\mathrm{raw}}\sim N\left(\gamma_{jF},{\hat{\sigma }}_{\gamma ,jF,\mathrm{raw}}^{2}\right),$$

where ${\hat{\sigma }}_{\gamma ,jF,\mathrm{raw}}$ is the estimated standard error of ${\hat{\gamma }}_{jF,\mathrm{raw}}$. The normality assumption here is appropriate due to the large GWAS sample sizes,^30,31^ regardless of the specific method used for estimation (maximum likelihood, method of moments, etc.).

### Potential specification of the prior distribution of female-specific SNP effect sizes and resulting posterior estimates

An intuitive way to borrow information from the male for the female population is to specify the prior mean and variance $\theta_{jF}$, $\tau_{jF}^{2}$ in Equation 1 as the male-specific effect size estimate and its estimated variance. Formally, the prior is

(Equation 3) $$\gamma_{jF}\sim N\left(\theta_{jF}={\hat{\gamma }}_{jM,raw},\tau_{jF}^{2}={\hat{\sigma }}_{\gamma ,jM,raw}^{2}\right),$$

leading to posterior

(Equation 4) $$\gamma_{jF}\left|{\hat{\gamma }}_{jF,raw},{\hat{\sigma }}_{\gamma ,jF,raw}^{2},{\hat{\gamma }}_{jM,raw},{\hat{\sigma }}_{\gamma ,jM,raw}^{2}\sim N\left(R_{jF},K_{jF}^{2}\right)\right.,$$

where the posterior mean $R_{jF}=\frac{{\hat{\sigma }}_{\gamma ,jM,\mathrm{raw}}^{2}}{{\hat{\sigma }}_{\gamma ,jF,\mathrm{raw}}^{2}+{\hat{\sigma }}_{\gamma ,jM,\mathrm{raw}}^{2}}\times {\hat{\gamma }}_{jF,\mathrm{raw}}+\frac{{\hat{\sigma }}_{\gamma ,jF,\mathrm{raw}}^{2}}{{\hat{\sigma }}_{\gamma ,jF,\mathrm{raw}}^{2}+{\hat{\sigma }}_{\gamma ,jM,\mathrm{raw}}^{2}}\times {\hat{\gamma }}_{jM,\mathrm{raw}}$ and the posterior variance $K_{jF}^{2}=\frac{1}{\frac{1}{{\hat{\sigma }}_{\gamma ,jF,\mathrm{raw}}^{2}}+\frac{1}{\sigma_{\gamma ,jM,\mathrm{raw}}^{2}}}$. The posterior mean is a weighted average of the prior mean and sample mean, with the weight on the group-specific estimates being proportional to their precisions (i.e., the inverse of their variances). The posterior mean and variance are exactly identical to those from conventional fixed-effects (FE) inverse-variance meta-analysis of the sex-specific estimates (${\hat{\gamma }}_{j,\mathrm{meta}}$, called the FE meta estimate henceforth). A more detailed illustration of the Bayesian approach to meta-analysis is given by Dominguez and Rice.^37^ The FE meta estimate has been shown to be as efficient as pooling individual-level data when effects are identical across combined studies,^38^ so in that setting, there is no penalty for using meta-analysis over any standard competing method.

However, the estimated strength of sex differences, i.e., ${\hat{\gamma }}_{j\mathrm{F,raw}}-{\hat{\gamma }}_{jM,\mathrm{raw}}$, is not considered in the FE meta estimate or standard competing methods, making their use unappealing when there are strong sex differences. To incorporate information on sex differences, we consider the prior with

(Equation 5) $$\gamma_{jF}\sim N\left(\theta_{jF}={\hat{\gamma }}_{j,\mathrm{meta}},\tau_{jF}^{2}={\left({\hat{\gamma }}_{jF,\mathrm{raw}}-{\hat{\gamma }}_{jM,\mathrm{raw}}\right)}^{2}+{\hat{\sigma }}_{\gamma ,jM,\mathrm{raw}}^{2}\right).$$

Here, the prior mean is the efficient FE meta estimate ${\hat{\gamma }}_{j,\mathrm{meta}}$, while the strength of sex differences is incorporated in the prior variance. The posterior distribution of $\gamma_{jF}$ is normal, with mean $R_{jF}=\frac{\hat{\delta^{2}}}{{\hat{\delta }}^{2}\;+\;{\hat{\psi }}^{2}}{\hat{\gamma }}_{jF,\mathrm{raw}}+\frac{\hat{\psi^{2}}}{{\hat{\delta }}^{2}\;+\;{\hat{\psi }}^{2}}{\hat{\gamma }}_{j,\mathrm{meta}}$, where ${\hat{\delta }}^{2}=\left({\hat{\gamma }}_{jF,\mathrm{raw}}-\right.{\left.{\hat{\gamma }}_{jM,\mathrm{raw}}\right)}^{2}+{\hat{\sigma }}_{\gamma ,jM,\mathrm{raw}}^{2}$ is related to both the difference in the effect size estimate and the variance of the male-specific estimate. Here, ${\hat{\psi }}^{2}$ is ${\hat{\sigma }}_{\gamma ,jF,raw}^{2}$. The posterior variance can be written as $K_{jF}^{2}=\frac{1}{\frac{1}{{\hat{\sigma }}_{\gamma ,jF,raw}^{2}}+\frac{1}{{\left({\hat{\gamma }}_{jF,raw}-{\hat{\gamma }}_{jM,raw}\right)}^{2}+{\hat{\sigma }}_{\gamma ,jM,raw}^{2}}}$. Because the posterior mean adapts ${\hat{\gamma }}_{jF,\mathrm{raw}}$ to the observed differences in sex-specific estimates, we call it the adaptive posterior mean (APM) estimator $\left({\hat{\gamma }}_{jF,APM}\right)$. From Equation 5, the prior mean and variance depend not only on sex-specific groups but also on sex-combined effect estimates and sex differences. This is why we refer to our framework as a “semi-empirical” Bayes approach.

The APM estimator is related to the AW estimator, initially proposed for gene-environment interactions or gene-secondary outcome associations in case-control studies.^34–36^ In both of those contexts, the AW estimator was developed to provide a population-level estimate (i.e., not specific to either cases or controls) by adaptively combining information from both groups using weighting. In our approach, we instead use shrinkage estimators to obtain group-specific estimates. The APM estimator also differs from the original AW in its weighting parameters. APM incorporates the variance of group-specific effect estimates into its prior variance for $\gamma_{jF}$, to avoid underestimating posterior variance due to smaller estimated sex differences (i.e., ${\hat{\gamma }}_{jF,\mathrm{raw}}-{\hat{\gamma }}_{jM,\mathrm{raw}}\approx 0$). In other words, if ${\hat{\sigma }}_{\gamma ,jM,\mathrm{raw}}^{2}=0$ in Equation 5, then the APM estimator may reduce to the AW estimator. Table 1 summarizes the derivation of the FE meta and APM estimates under the Bayesian normal-normal modeling scheme. As stated earlier, we focus on the calibration of the female effects ${\hat{\gamma }}_{F}$, but the framework is general and can be similarly applied to the male population.

### Exposure-outcome causal effect estimation

**Figure 1.**
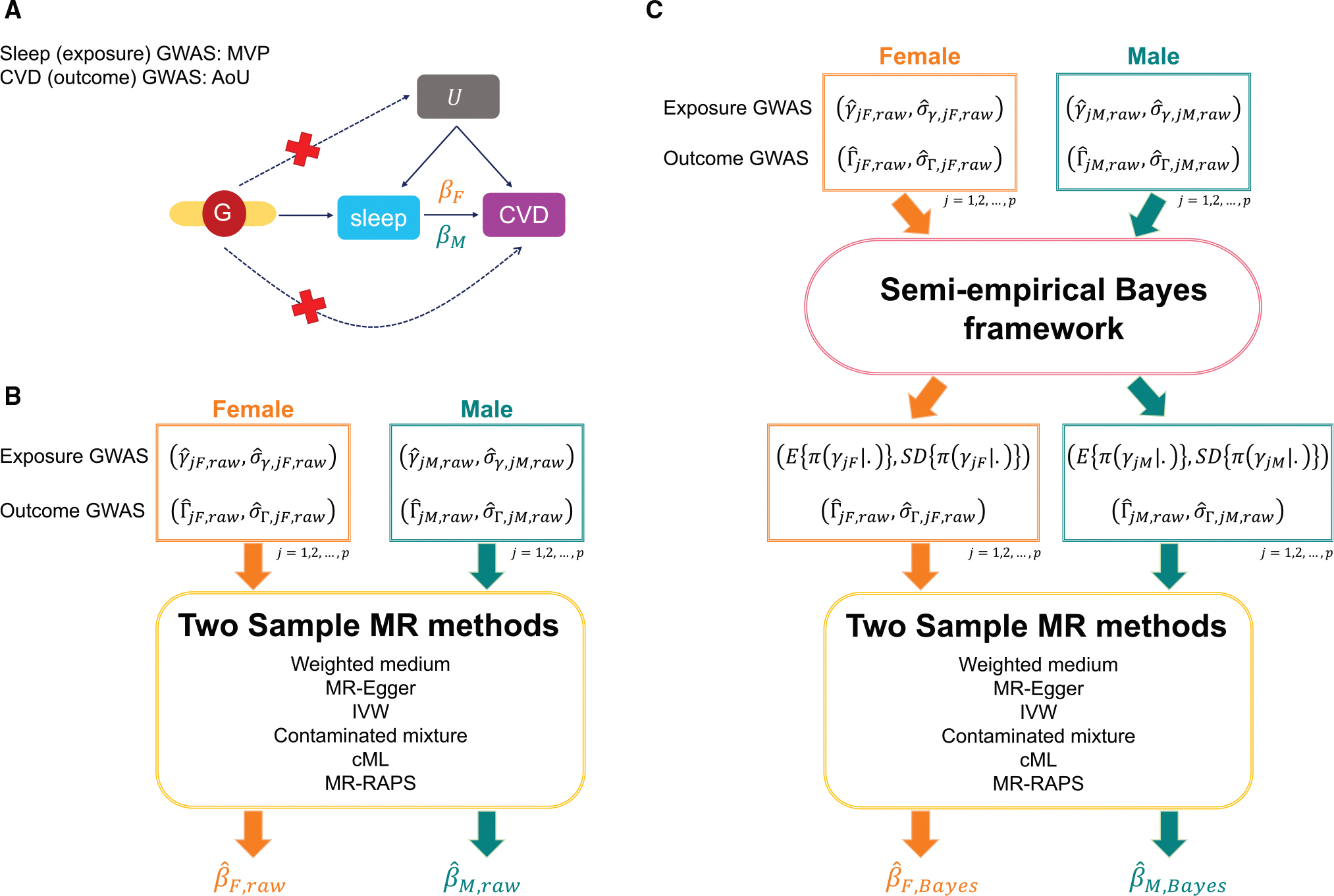
Schematic overview of the sex-specific MR analysis (A) A causal diagram underlying the MR framework in this manuscript. We consider sleep-related phenotypes to be the exposure factors and cardiovascular-related measures as outcome variables. $\beta_{F}$ denotes the underlying female-specific exposure-outcome causal effect; $\beta_{M}$ represents the underlying male-specific exposure-outcome causal effect. In our analysis, $\beta_{F}$ and $\beta_{M}$ are estimated separately. The GWAS summary statistics for sleep phenotypes were derived from the MVP dataset, and the summary statistics for cardiovascular-related diseases were computed in AoU. (B) Estimation of sex-specific causal effects using the two-sample MR approaches. The inputs for the two-sample MR methods are raw sex-specific exposure (female: ${\hat{\gamma }}_{jF,\mathrm{raw}}$ and ${\hat{\sigma }}_{\gamma ,jF,\mathrm{raw}}$; male: ${\hat{\gamma }}_{jM,\mathrm{raw}}$ and ${\hat{\sigma }}_{\gamma ,jM,\mathrm{raw}}$) and outcome GWAS summary statistics (female: ${\hat{\Gamma }}_{jF,\mathrm{raw}}$ and ${\hat{\sigma }}_{\Gamma ,jF,\mathrm{raw}}$; male: ${\hat{\Gamma }}_{jM,\mathrm{raw}}$ and ${\hat{\sigma }}_{\Gamma ,jM,\mathrm{raw}}$) from independent samples. The outputs are estimated female-specific causal effect ${\hat{\beta }}_{F,\mathrm{raw}}$ and estimated male-specific causal effect ${\hat{\beta }}_{M,\mathrm{raw}}$. (C) The proposed Bayesian method. We first calibrate the raw sex-specific exposure effects by borrowing information from one sex group to use in the other or across both sex groups. Two-sample MR analysis then uses the shrinkage exposure summary statistics, i.e., the posterior mean and posterior SD of γ (female: $E\left\{\pi \left(\gamma_{jF}|\cdot \right)\right\}$ and $SD\left\{\pi \left(\gamma_{jF}|\cdot \right)\right\}$; male: $E\left\{\pi \left(\gamma_{jM}|\cdot \right)\right\}$ and $SD\left\{\pi \left(\gamma_{jM}|\cdot \right)\right\}$), with the raw outcome summary statistics to provide a more robust basis for causal effect estimation. We use π(γ|.) to denote the posterior distribution of γ. The outputs are the estimated female-specific causal effect ${\hat{\beta }}_{F,\mathrm{Bayes}}$ and estimated male-specific causal effect ${\hat{\beta }}_{M,\mathrm{Bayes}}$; both use shrinkage exposure effect estimates in their construction. MR, Mendelian randomization; F, female; M, male; CVD, cardiovascular disease; GWAS, genome-wide association study; MVP, Million Veteran Program; AoU, All of Us; SD, standard deviation; cML, constrained maximum likelihood.

Two-sample MR approaches estimate causal effects from two independent sets of summary statistics. These describe variant associations with an exposure and with the outcome phenotype, where the variants are selected to be valid IVs for the exposure of interest.^30,39–42^ A causal effect $\beta_{j}$ of exposure on outcome can then be estimated using estimated associations of variant j with exposure (${\hat{\gamma }}_{j,\mathrm{raw}}$) and outcome (${\hat{\Gamma }}_{j,\mathrm{raw}}$) via the Wald ratio estimate ${\hat{\beta }}_{j,\mathrm{raw}}=\frac{{\hat{\Gamma }}_{j,\mathrm{raw}}}{{\hat{\gamma }}_{j,\mathrm{raw}}}$. Using multiple IVs, the causal effect β can then be estimated by aggregating estimates from all valid IVs through various weighting approaches. Here, we assume that all variants are valid IVs. Our proposed Bayesian framework, therefore, is conceptualized as a preliminary step before applying MR methods. More specifically, we first calibrate the ${\hat{\gamma }}_{\mathrm{raw}}$ estimates using the proposed Bayesian models. Then, the shrinkage estimates, i.e., the posterior means and SDs in Equation 4, are used as inputs in existing MR algorithms. The causal effect is then estimated using the newly estimated ${\hat{\gamma }}_{\mathrm{meta}}$ and ${\hat{\gamma }}_{APM}$ (with corresponding posterior SD) coupled with the (raw) variant-outcome estimated effect sizes. A schematic overview of the sex-specific MR analysis and the proposed Bayesian framework is illustrated in Figure 1. We also consider multivariable MR (MVMR), which may be used to account for potential confounders (see Appendix A).

## Results

### Simulation studies

We used simulations primarily to evaluate and compare the performance of exposure-outcome causal effect (β) estimation using raw (${\hat{\gamma }}_{\mathrm{raw}}$) and shrinkage (${\hat{\gamma }}_{\mathrm{meta}}$ and ${\hat{\gamma }}_{APM}$) variant-exposure effects with respective SD estimates, combined with each selected MR method, particularly focusing on the population with a smaller sample size (female population in our analysis). For our Bayesian methods, the posterior SDs of γ are treated as standard error estimates. We also incorporated the AW estimator (${\hat{\gamma }}_{\mathrm{AW}}$) as another approach for calibrating female-specific variant-exposure effect size estimates. The two-sample MR methods considered in the analyses are summarized in Table 2 (Appendix A).

To mimic the structure of the MVP dataset, we generated 2,000 female individuals and 20,000 male individuals for the exposure GWAS, maintaining a similar proportion of females to males as in the MVP. For the outcome GWAS, we generated balanced datasets of 10,000 individuals for both female and male populations. We generated 100 independent SNPs as IVs in all simulations, with all allele frequencies set at 0.3. We use $D_{j\gamma }=\gamma_{jF}-\gamma_{jM}$, j=1,2,…,p to denote the strength of sex differences in variant associations with the exposure, γ. We write $D_{\gamma }=\left\{j|D_{j\gamma }=\gamma_{jF}-\gamma_{jM}\neq 0\right\}$ and $\left|D_{\gamma }\right|$ to represent the set of variants and the number of variants with sex differences in variant-exposure effect size, respectively. We considered three simulation scenarios: (1) fixed $D_{j\gamma }=0.05$ if variant $j\in D_{\gamma }$, (2) random $D_{j\gamma }$, and (3) using MVP OSA GWAS summary statistics to guide the simulated differences $D_{j\gamma }$, which consists of strong $D_{j\gamma }$ patterns with weak IVs in the female population (average F-statistic < 10). In simulations 2 and 3, all variants $j\in D_{\gamma }$ (i.e., $\left|D_{\gamma }\right|=100$). Within each simulation, we also considered a few levels of sex differences in causal effect settings (i.e., $\beta_{F}\neq \beta_{M}$). A more detailed description of the simulation studies is provided in Appendix A, Note S1, and Tables S1 and S2. Table S3 summarizes the average F-statistic (across SNPs and across simulation repetitions), which measures the average strength of IVs, when using ${\hat{\gamma }}_{\mathrm{raw}}$ and ${\hat{\gamma }}_{\mathrm{APM}}$. Across simulation settings, the average F-statistics derived from ${\hat{\gamma }}_{APM}$ in the female stratum are more than 3-fold higher than those from ${\hat{\gamma }}_{\mathrm{raw}}$, demonstrating improved IV strength.

**Figure 2.**
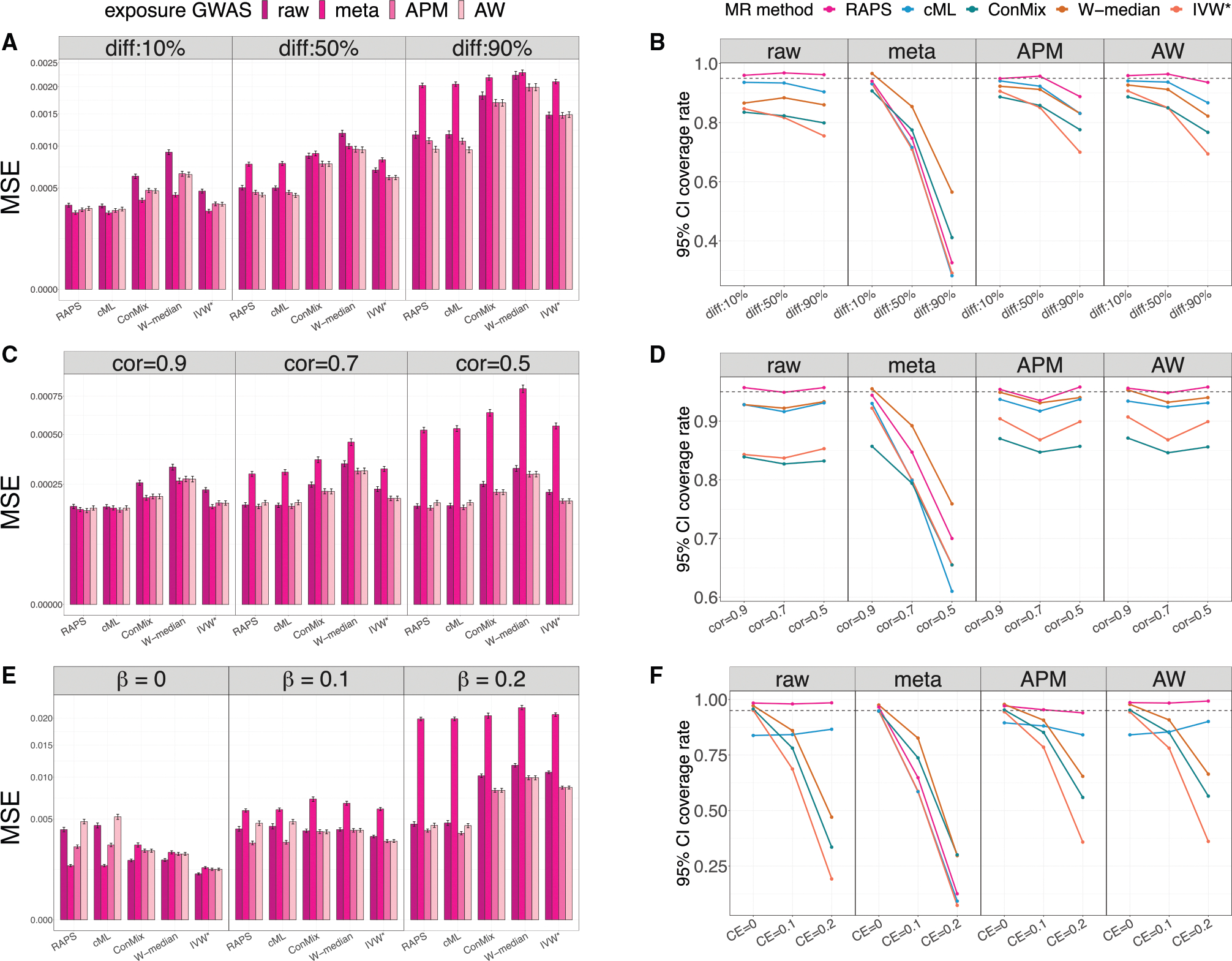
MSE and 95% confidence interval coverage rate for female-specific causal effect estimation Results from estimating female-specific causal effects $\beta_{F}$ under three simulation scenarios where there are no sex differences in causal effects (i.e., $\beta_{F}=\beta_{M}$) across sex groups. The left image shows the MSEs of the estimated female-specific causal effects, while the right image presents the 95% confidence interval coverage rates of the true underlying causal effects. We considered five two-sample MR methods for estimating the causal effect, which are MR-RAPS (RAPS), constrained maximum likelihood (cML), contaminated mixture (ConMix), weighted median (W-median), and penalized and robust IVW (IVW*). In the MSE results, the uncalibrated approach (${\hat{\gamma }}_{\mathrm{raw}}$) is represented by the bars with darker colors. The other three shrinkage approaches (${\hat{\gamma }}_{\mathrm{meta}}$, ${\hat{\gamma }}_{APM}$, and ${\hat{\gamma }}_{AW}$) are shown in gradient colors. For the coverage rate results, the uncalibrated approach is shown in the first column, and the other three shrinkage approaches are shown in the second to fourth columns. The results of fixed sex differences in variant-exposure effect simulations are presented in (A) and (B). The results of random sex differences in variant-exposure effect simulations are shown in (C) and (D). The results of using MVP OSA GWAS summary statistics to guide sex-specific variant-exposure effect simulations are shown in (E) and (F). The underlying true causal effect is set at $\beta_{F}=0.1$ in (A)–(D). The underlying true causal effects are shown on the top of (E) and the bottom of (F). MSEs were computed over 1,000 simulation replicates. Intervals around the estimated MSE correspond to the MSE ± one estimated standard error. MSE, mean square error; MR, Mendelian randomization; APM, adaptive posterior mean; AW, adaptive weight; diff, different level of sex differences in variant-exposure effects; Cor, correlation between female and male variant-exposure effect; CE, causal effect; RAPS, MR-RAPS; cML, constrained maximum likelihood; ConMix, contaminated mixture; W-median, weighted medium; IVW*, penalized and robust IVW.

We summarized the estimation performance of the ${\hat{\beta }}_{F}$ and ${\hat{\beta }}_{M}$ in the simulation studies using two metrics: (1) the mean-squared error (MSE) of the estimated effect and (2) the 95% confidence interval (CI)’s actual coverage of the true effect, where these 95% CIs for β under each MR method were computed using standard asymptotic normality properties. The female results from simulations with no sex differences in the causal effect are presented in Figure 2 and summarized below. The full simulation results, including the male-specific causal effect estimation and sex differences in causal effect simulations, are summarized in Note S1 and Figures S1–S3.

#### Results from simulation setting 1: Fixed sex differences in variant-exposure effect sizes

These results are provided in Figures 2A and 2B. The ${\hat{\gamma }}_{\mathrm{meta}}$ estimator performs best when $D_{\gamma }$ includes only 10% of the variants (i.e., $\left|D_{\gamma }\right|=10$), compared to other shrinkage methods and to the raw ${\hat{\gamma }}_{F,raw}$. The estimators ${\hat{\gamma }}_{F,APM}$ and ${\hat{\gamma }}_{F,AW}$ had similar performance, and both gave better causal effect estimates than ${\hat{\gamma }}_{F,\mathrm{raw}}$, regardless of the proportion of variants with sex differences. We found that ${\hat{\gamma }}_{F,APM}$ performed better than ${\hat{\gamma }}_{\mathrm{meta}}$ when more than 10% of variants were being selected into $D_{\gamma }$. The coverage rate of $\beta_{F}$ using ${\hat{\gamma }}_{F,APM}$ was lower than that of ${\hat{\gamma }}_{F,\mathrm{raw}}$ when many variants were included in $D_{\gamma }$. However, ${\hat{\gamma }}_{F,APM}$ always achieved a smaller MSE for $\beta_{F}$ compared with ${\hat{\gamma }}_{F,\mathrm{raw}}$.

#### Results from simulation setting 2: Random sex differences in variant-exposure effect sizes

For random sex differences in γ (Figures 2C and 2D), when every variant has a sex difference, i.e., all $j\in D_{\gamma }$, and most sex difference $D_{j\gamma }$ were strong, the estimate ${\hat{\beta }}_{F}$ that relies on the ${\hat{\gamma }}_{\mathrm{meta}}$ had a higher MSE than the one relying on raw ${\hat{\gamma }}_{F,\mathrm{raw}}$. In contrast, using ${\hat{\gamma }}_{F,APM}$ improved $\beta_{F}$ estimation performance in terms of MSE and performed similarly to ${\hat{\gamma }}_{F,AW}$. In these simulations, the APM estimator ${\hat{\gamma }}_{F,APM}$ resulted in similar 95% CI coverage compared to the raw approach but achieved a smaller MSE for $\beta_{F}$.

#### Results from simulation setting 3: Using OSA GWAS summary statistics to guide the simulated variant-exposure effect sizes

Here, all variants have substantial sex differences in γ, while the selected variants are all weak IVs, meaning that the F-statistic is < 10 in the female population. Using ${\hat{\gamma }}_{F,APM}$ and ${\hat{\gamma }}_{F,AW}$ improved $\beta_{F}$ estimation as demonstrated by the improved MSE (Figures 2E and 2F). In most cases, ${\hat{\gamma }}_{F,APM}$ performed better than ${\hat{\gamma }}_{F,AW}$. Both ${\hat{\gamma }}_{F,APM}$ and ${\hat{\gamma }}_{F,AW}$ approaches improved the 95% CI coverage rate compared to using ${\hat{\gamma }}_{F,raw}$. These results highlight that even though less information could be transferred from the male to the female population in these simulations (due to the strong sex differences in γ), adaptive estimates still improved causal effect estimation. Moreover, the estimates ${\hat{\gamma }}_{\mathrm{meta}}$ had a larger MSE than the raw approach under most settings and performed poorly when the female causal effect $\beta_{F}$ was non-null.

In summary, borrowing power from the stratum with the larger sample size to give to the stratum with the lower sample size using adaptive (i.e., shrinkage) variant-exposure estimates (${\hat{\gamma }}_{F,APM}$ and ${\hat{\gamma }}_{F,AW}$) improves $\beta_{F}$ estimates. Using estimators ${\hat{\gamma }}_{F,APM}$ and ${\hat{\gamma }}_{F,AW}$ performed well in most simulation studies, regardless of the degree of sex differences $D_{\gamma j}$. The estimator ${\hat{\gamma }}_{\mathrm{meta}}$ performs best when the underlying true γ were similar in the two groups. Among the two-sample MR methods considered, MR using robust adjusted profile score (RAPS), known to perform well when weak instruments are used, demonstrated at least no worse performance than the other methods.

### Secondary simulation studies

Note S2 and Figures S4–S16 provide results from secondary simulation studies. We expanded upon simulation setting 1 and further examined two scenarios: (1) estimation in the presence of pleiotropic effects (both balanced and directional) of some IVs and (2) calibration of both γ and Γ effect estimates using the proposed framework. We also considered an increased sample size of the female population in the exposure GWAS to simulate a scenario where borrowing information from the male group may be less useful. Lastly, we evaluated the performance of a test for sex differences, i.e., a test of the null hypothesis $H_{0}:\beta_{F}=\beta_{M}$, using the estimated $\beta_{F}$ and $\beta_{M}$ based on various MR approaches (but always using the raw variant-exposure ${\hat{\gamma }}_{\mathrm{raw}}$ and variant-outcome associations ${\hat{\Gamma }}_{\mathrm{raw}}$).

In brief, ${\hat{\gamma }}_{APM}$ and ${\hat{\gamma }}_{AW}$ substantially improved the estimation accuracy of γ (Figures S4–S6) compared to the ${\hat{\gamma }}_{\mathrm{raw}}$ in all settings and, under substantial sex differences, performed better than ${\hat{\gamma }}_{\mathrm{meta}}$. In the simulations where pleiotropy was present, ${\hat{\gamma }}_{APM}$ still produced a lower MSE when estimating $\beta_{F}$ compared to ${\hat{\gamma }}_{\mathrm{raw}}$ (Figures S7 and S9). The penalized and robust IVW, contaminated mixture, constrained maximum likelihood (cML), and MR-RAPS performed similarly in the balanced pleiotropy analysis. In the directional pleiotropy analysis (Figures S9 and S10), where the assumptions of the MR-RAPS method are violated, MR-RAPS showed reduced power, with the 95% coverage rate most affected due to underestimation of standard error. In terms of MSE, MR-RAPS performed less well compared to the robust IVW, performed similarly to cML, and still outperformed traditional methods such as MR-Egger and IVW. Additional calibration of Γ improved the estimation of the $\beta_{F}$ when males and females had the same causal effect but not otherwise (Figures S11 and S12). When increasing female sample sizes, the shrinkage approaches resulted in nearly the same MSE for $\beta_{F}$ estimation as that for ${\hat{\gamma }}_{\mathrm{raw}}$, indicating that no estimation efficiency is lost when using the shrinkage approaches, even though potentially less information is transferred from the male population (Figures S13 and S14). The test of sex differences in the causal effect between groups showed that MR-RAPS controls type I error rate close to the nominal level under most settings while maintaining similar power compared to other MR methods for detecting sex differences in causal effects (Figures S15 and S16).

### Sex-specific causal estimates of the effect of sleep phenotypes on cardiovascular-related outcomes

We estimated sex-specific causal effects of sleep phenotypes on CVD-related outcomes. Specifically, we considered five binary sleep traits: OSA, insomnia, short sleep duration, long sleep duration, and excessive daytime sleepiness (sleepiness). The outcomes were six binary CVD-related phenotypes, with data from AoU: atrial fibrillation (AF), CAD, CKD, heart failure (HF), HTN, and type 2 diabetes mellitus (T2DM). Detailed information on the AoU analysis is provided in Note S4. A summary of the results from MVP GWASs, including sample sizes for sex-specific sleep trait GWASs, Miami plots, and Q-Q plots, is presented in Note S5. The MVP sex-specific GWASs of sleep phenotypes were conducted using the same procedure described elsewhere.^16^ We only included individuals from the White harmonized race/ethnicity and genetic ancestry (HARE) group, as the large HARE group. Based on these results, we selected variants and performed variant-outcome associations in the AoU dataset, focusing on the group of White individuals. For each trait, two types of analyses were performed: one adjusting for body mass index (BMI) and the other without BMI adjustment, in both the exposure GWAS and outcome association analyses. BMI was adjusted for because it is recognized as a strong risk factor for sleep phenotypes. For example, high BMI is the strongest risk factor for OSA. This adjustment aims to estimate sleep-CVD causal effects in pathways that are independent of BMI. We only used common variants (minor-allele frequency ≥ 0.01) with imputation quality scores ≥ 0.8. More details are summarized in Appendix A.

### IV selection strategies

**Figure 3.**
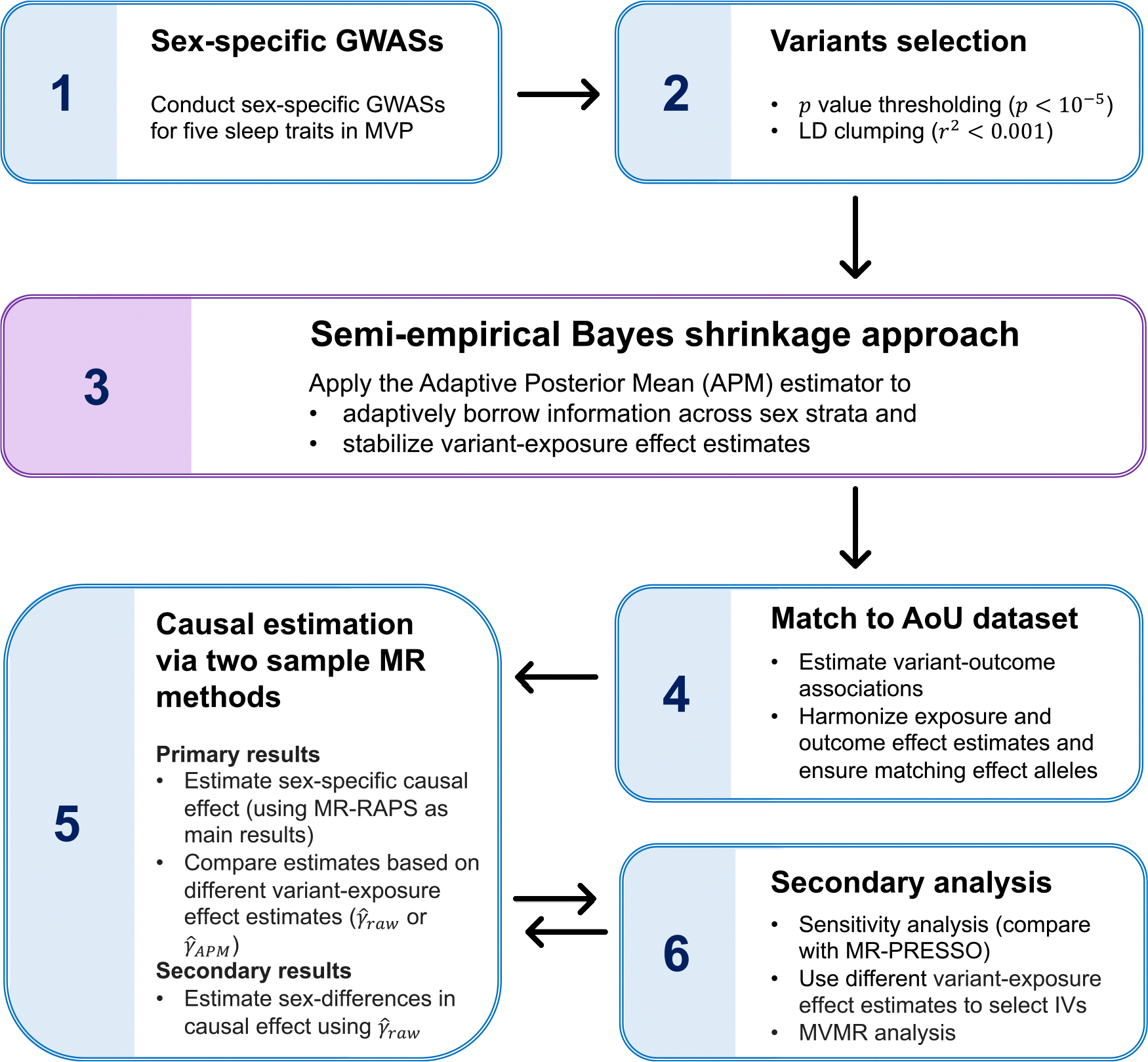
Overview of data analysis workflow The main workflow consists of six key steps. In step 1, we performed sex-specific GWASs of five sleep traits using participants from MVP. Step 2 involved the selection of IVs based on the sleep GWASs. In step 3, we applied our proposed semi-empirical Bayesian approach to stabilize the variant-exposure effect estimates, particularly for the female group, which had a small sample size in MVP. Step 4 involved harmonizing the exposure and outcome datasets by aligning allele effects, where the variant-outcome associations were computed from the AoU dataset. In step 5, we conducted the primary two-sample MR analysis using the MR-RAPS method to estimate sex-specific causal effects, followed by the testing of sex differences in causal effect estimates. Finally, in step 6, we conducted secondary analyses, including sensitivity analyses comparing results from MR-RAPS and MR-PRESSO, evaluating alternative IV selection strategies based on different variant-exposure effect estimates and MVMR analysis. GWAS, genome-wide association study; MVP, Million Veteran Program; IV, instrumental variable; AoU, All of Us; MR, Mendelian randomization; MVMR, multivariable MR.

We applied *p* value thresholding and clumping procedures on exposure GWASs using the “clump_data” function from the “TwoSampleMR” R package (v.0.6.8; https://mrcieu.github.io/TwoSampleMR/reference/clump_data.html). The clumping window was set to 10,000 kb, the correlation threshold was set to 0.001, and the European population reference panel was used. Due to having a limited number of variants (or even no variants) with $p\;<\;5\;\times \;10^{-8}$ and $p\;<\;10^{-7}$ in the female sleep GWASs, a *p* value threshold of 10^−5^ was selected for IV selection in all analyses. The number of variants remaining after *p* value thresholding ($p\;<\;10^{-5}$) and clumping is given in Table S4. We then matched the list of variants selected as targeted IVs from the exposure GWAS to those available in the AoU dataset. Harmonization of the exposure and outcome datasets was performed using the “harmonise_data” function from the TwoSampleMR R package (v.0.6.8; https://mrcieu.github.io/TwoSampleMR/reference/harmonise_data.html). This function ensures that the effect estimates for each variant are aligned to the same effect allele across exposure and outcome datasets by checking both the direction of effect sizes and the allele frequency of the reported “effect allele.” It also accounts for strand ambiguity and removes variants with mismatched allele frequencies or palindromic alleles that cannot be reliably aligned. Detailed instructions for the harmonization procedure can be found at https://mrcieu.github.io/TwoSampleMR/articles/harmonise.html. We employed two strategies for IV selection in the primary analyses: (1) sex-specific IVs were selected based on sex-specific GWAS results (${\hat{\gamma }}_{\mathrm{raw}}$), potentially resulting in different variants for male and female analyses, and (2) IVs were selected using APM shrinkage estimates (${\hat{\gamma }}_{APM}$), where additional “potential” IVs that could not be selected using ${\hat{\gamma }}_{F,\mathrm{raw}}$ estimates (due to the smaller female sample size) were included by borrowing information from the male population. In the secondary analysis, we applied the ${\hat{\gamma }}_{\mathrm{meta}}$ estimate for IV selection in sex-combined analyses. Figure 3 presents a flowchart summarizing the data analysis workflow.

### Sex-specific causal estimates

**Figure 4.**
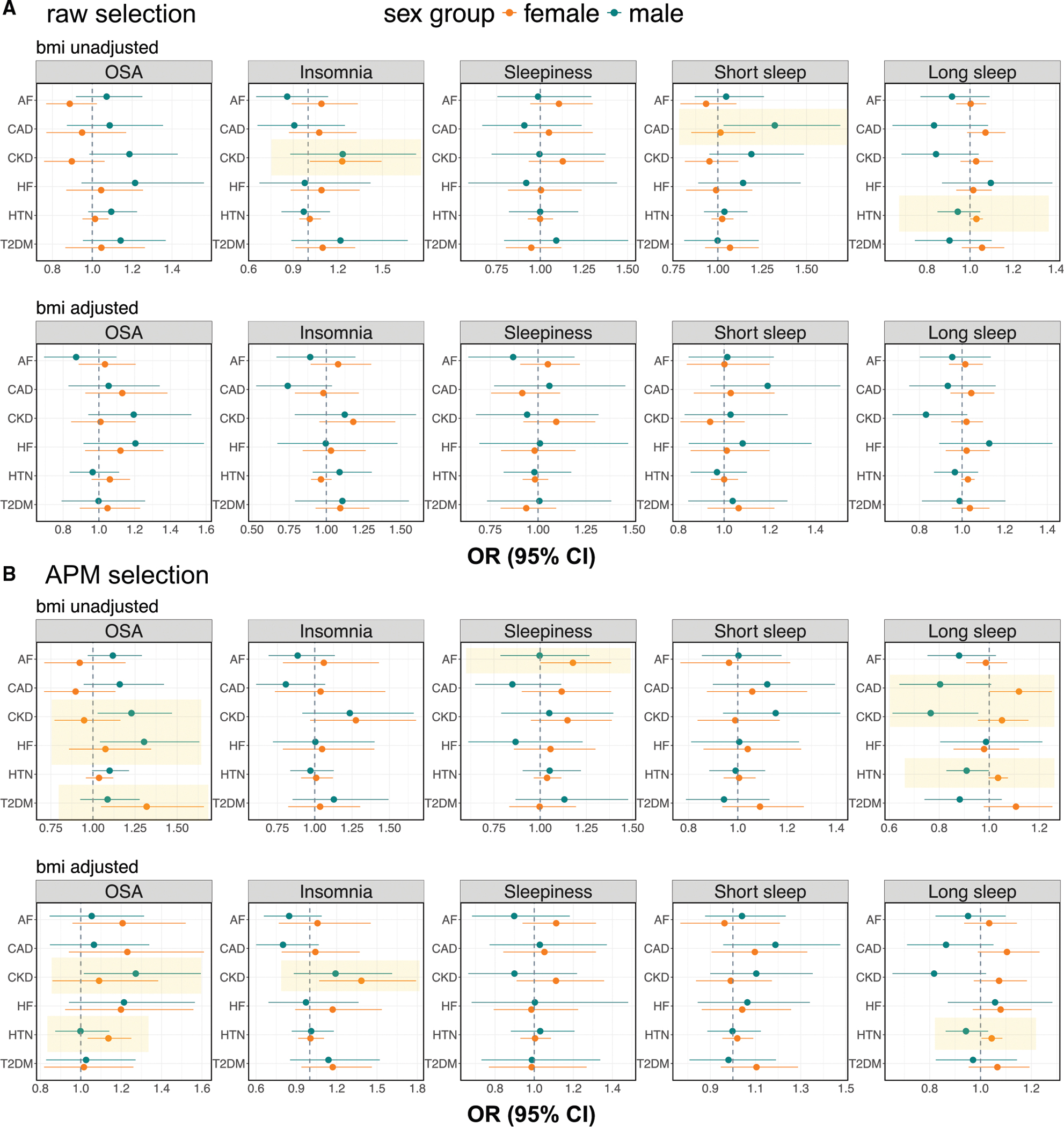
Results from sex-specific causal effect estimation (A) Provides sex-specific causal effect estimates with the corresponding 95% CIs based on IVs selected by using ${\hat{\gamma }}_{\mathrm{raw}}$, while (B) shows results using ${\hat{\gamma }}_{\mathrm{APM}}$ for IV selection. The estimated causal effects (from MR-RAPS) with ${\hat{\gamma }}_{\mathrm{APM}}$ variant-exposure effect estimates are displayed on an OR scale. In each image, variant-phenotype estimates without BMI adjustment are displayed at the top, and those with BMI adjustment are shown on the bottom. Female-specific results are indicated in orange, while male-specific results are shown in green. Vertical dashed lines indicate the null causal effects. The exposure variables are shown as the titles of the boxes, while the row names provide the outcome variables. Statistically significant results (p<0.05) for either the female- or male-specific analysis are highlighted with yellow background. CI, confidence interval; APM, adaptive posterior mean; IV, instrumental variable; MR-RAPS, MR using robust adjusted profile score method; MR, Mendelian randomization; OR, odds ratio; BMI, body mass index; OSA, obstructive sleep apnea; AF, atrial fibrillation; CAD, coronary artery disease; CKD, chronic kidney disease; HF, heart failure; HTN, hypertension; T2DM, type 2 diabetes mellitus.

The primary results use the ${\hat{\gamma }}_{APM}$ estimates with the MR-RAPS method, specifically developed for handling weak instruments and demonstrating superior performance in simulation studies. Figure 4 shows sex-specific causal effect estimates of sleep-related phenotypes on CVD-related outcomes, employing two different IV selection strategies: IVs selected based on sex-specific ${\hat{\gamma }}_{APM}$ (primary, proposed) and sex-specific ${\hat{\gamma }}_{\mathrm{raw}}$. Three pairs of causal effect estimates showed statistically significant associations (p<0.05) when IVs were selected using ${\hat{\gamma }}_{\mathrm{raw}}$: insomnia on CKD in females (odds ratio [OR]: 1.23, 95% CI: 1.01–1.49), long sleep on HTN in females (OR: 1.03, 95% CI: 1.00–1.06), and short sleep on CAD in males (OR: 1.32, 95% CI: 1.03–1.69). In contrast, when ${\hat{\gamma }}_{\mathrm{APM}}$ was used for IV selection, enabling more potential IVs to be selected by borrowing information across sex groups, additional statistically significant causal estimates were identified. For instance, in females, we found a significant effect of OSA on T2DM (OR: 1.32, 95% CI: 1.05–1.66) in BMI-unadjusted analyses and also an effect on HTN (OR: 1.14, 95% CI: 1.03–1.25) in BMI-adjusted analyses. Notably, the causal relationships between insomnia and CKD (OR: 1.38, 95% CI: 1.07–1.79) and long sleep and HTN (OR: 1.04, 95% CI: 1.00–1.09) were replicated in the APM selection analyses (both from BMI-adjusted analyses). Among males, multiple causal associations were observed between OSA and CVD-related outcomes, including OSA on CKD (BMI unadjusted, OR: 1.23 and 95% CI: 1.03–1.47; BMI adjusted, OR: 1.27 and 95% CI: 1.02–1.59) and OSA on HF in the BMI-unadjusted analysis (OR: 1.30, 95% CI: 1.04–1.63).

Sex difference tests identified a statistically significant difference in the causal effect of OSA on CKD, with a stronger effect in males (Table 3; Figure S17). Also, there were sex differences in the causal effects of long sleep on several CVD-related outcomes: long sleep increased the risk of CAD, CKD, HTN, and T2DM in females but was protective in males. These sex differences were statistically significant in the APM IV selection analysis (Table 3; Figures S19 and S20).

The key findings from sex-specific causal effect estimation and sex difference tests are summarized in Tables 3 and 4. Full results, including the causal estimation using ${\hat{\gamma }}_{\mathrm{raw}}$, ${\hat{\gamma }}_{\mathrm{meta}}$, and ${\hat{\gamma }}_{AW}$, as well as the sex differences in causal estimates tests, are summarized in Figures S17–S20. Causal estimates from other considered MR methods are summarized in Data S1 (${\hat{\gamma }}_{\mathrm{raw}}$ IV selection) and S2 ${\hat{\gamma }}_{\mathrm{APM}}$ IV selection). The average F-statistics are summarized in Table S5. Focusing on the female population, when selecting IVs using sex-specific ${\hat{\gamma }}_{\mathrm{raw}}$, the average F-statistics are similar between ${\hat{\gamma }}_{\mathrm{raw}}$ and ${\hat{\gamma }}_{APM}$, consistent with the fairly similar causal effect estimates between analyses that rely on ${\hat{\gamma }}_{\mathrm{raw}}$ and ${\hat{\gamma }}_{\mathrm{APM}}$ when selecting IVs based on ${\hat{\gamma }}_{\mathrm{raw}}$ (Figures S17 and S18). In contrast, when IVs are selected based on ${\hat{\gamma }}_{APM}$, the average F-statistic is substantially higher for ${\hat{\gamma }}_{\mathrm{APM}}$ compared to ${\hat{\gamma }}_{\mathrm{raw}}$ because selecting IVs based on ${\hat{\gamma }}_{APM}$ identified SNPs that had relatively weak ${\hat{\gamma }}_{\mathrm{raw}}$ associations in the female stratum before borrowing information from the male stratum. Indeed, several causal associations are statistically significant in female-specific analysis only when using ${\hat{\gamma }}_{\mathrm{APM}}$ IV selection.

### Results from secondary analyses

We compared the causal effect estimates of MR-RAPS, which we used in the primary analyses, with those of MR-PRESSO,^44^ a widely used approach for detecting IVs with pleiotropic effects and removing them in MR analysis. The results from the two methods are similar and are summarized in Note S3 and Figures S21 and S22.

A comparison of causal effect estimates using ${\hat{\gamma }}_{\mathrm{raw}}$ and ${\hat{\gamma }}_{\mathrm{APM}}$ for IV selection is presented in Figure S23. In the male population, more consistent results were observed across different IV selection methods. For the analysis of the OSA phenotype, the ${\hat{\gamma }}_{APM}$ selection strategy identified more variants as IVs in both male and female populations. Several significant causal effects between OSA and CVD-related outcomes were only detected when ${\hat{\gamma }}_{\mathrm{APM}}$ was used for IV selection. These findings highlight an additional advantage of our proposed shrinkage estimate: it not only helps correct for weak IV bias in causal effect estimation but also enhances the IV selection process, increasing the potential for identifying novel causal effects.

We applied ${\hat{\gamma }}_{\mathrm{meta}}$ for IV selection in the sex-combined analysis. In this analysis, the variant-exposure effect estimates were based on ${\hat{\gamma }}_{\mathrm{meta}}$, and the variant-outcome effect estimates were computed from a sex-combined analysis in the AoU study. The results are shown in Figure S24. Significant causal relationships between OSA and several CVD-related outcomes were identified, consistent with the findings from the primary analysis using ${\hat{\gamma }}_{APM}$ for IV selection. However, some of the significant sex-specific findings, such as the female-specific causal effect of long sleep on HTN, were not identified in sex-combined analysis, likely because the ${\hat{\gamma }}_{\mathrm{meta}}$ is closed to ${\hat{\gamma }}_{M,\mathrm{raw}}$ due to the predominantly male sample size in MVP.

Lastly, as another approach for estimation of causal effects while accounting for BMI as a common cause of sleep exposures and CVD outcomes, we applied MVMR to estimate BMI-adjusted causal effects while using summary statistics derived from the BMI-unadjusted GWAS. Specifically, BMI was incorporated as an additional exposure in the MVMR model. We applied the robust multivariable inverse-variance weighted (MV-IVW) method in the MVMR analysis. Results from the MVMR analysis are presented in Figure S25, Table S6, and Data S3, with further details provided in Note S3 and Appendix A. We compare the MVMR results to the two sets of results: (1) univariate MR using BMI-unadjusted summary statistics (as this analysis relies on exactly the same set of IVs as the univariate analysis) and (2) using BMI-adjusted summary statistics (as this analysis has the same purpose of estimating causal effects that are independent of BMI).

First, results from comparing MVMR to univariate MR using BMI-unadjusted summary statistics are shown in Figures S26 and S27. Focusing on the primary univariate MR analysis (MR-RAPS), most exposure-outcome associations retained the same direction of effect estimates. Several associations also remained statistically significant, including the increased risk of CKD associated with insomnia in females, the increased risk of CAD associated with short sleep duration in males, and the protective effects of long sleep duration on CKD and HTN in males. However, some associations, specifically in OSA analysis, were attenuated and no longer statistically significant in the MVMR analysis. For instance, the male-specific causal effect of OSA on HF was estimated at an OR of 1.30 (95% CI: 1.04–1.63) in the univariable analysis but decreased to 1.09 (95% CI: 0.82–1.45) in MVMR; the female-specific effect of OSA on T2DM was 1.32 (95% CI: 1.05–1.66) in the univariable analysis, compared to 1.13 (95% CI: 0.85–1.49) in MVMR.

Second, we compared the BMI-adjusted univariable MR analysis to the MVMR results. This time, there were a few strong differences between the results. For example, no significant associations were identified in the BMI-adjusted GWAS using the raw selection method, whereas MVMR detected several significant findings, as described above. This is not unexpected: raw selection has lower power, especially in BMI-adjusted analysis, as estimated genetic associations with the sleep exposures are weaker compared to those from BMI-unadjusted analysis. Using APM for IV selection, the causal effect of OSA on HTN in females was significant (OR: 1.14, 95% CI: 1.03–1.25) in the univariable analysis but became non-significant in MVMR (OR: 1.03, 95% CI: 0.95–1.11), and the protective effects of long sleep duration on CKD and CAD were significant in MVMR but not in the BMI-adjusted GWAS univariate MR analysis. These differences may be attributed to differences in IV selection, as well as to the approach for BMI adjustment itself. There were a few cases of differences in the directions of the estimated associations, but all associations with flipped directions between analyses were not statistically significant.

## Discussion

We performed sex-specific analysis of the causal associations of sleep-related phenotypes on CVD-related outcomes. These are biologically important relationships with immediate clinical relevance. The primary rationale in our work was to ameliorate the weak instrumental bias in MR analysis. This can be done by incorporating information from auxiliary datasets. In our case, we used a male-specific dataset to improve female-specific statistics. Acknowledging likely sex differences between male and female individuals, this led to the need for an adaptive estimator, which will intelligently utilizes information across the two sex groups. Female-specific causal estimation is limited by smaller sample sizes (relative to males), leading to large variability in $\gamma_{F}$ estimates, also known as weak IV bias. Thus, we introduced a framework to calibrate the ${\hat{\gamma }}_{F}$ estimates by borrowing information from the male group. We first demonstrated that the FE meta estimate (${\hat{\gamma }}_{\mathrm{meta}}$) is a special case of our proposed framework. We then proposed the APM estimate, which adaptively transfers information across sex groups by considering the strength of the sex difference in $\hat{\gamma }$ in a data-driven manner. Simulation studies demonstrated that (1) employing the shrinkage estimates can substantially improve the efficiency of causal estimation for the population with smaller sample size, (2) using the ${\hat{\gamma }}_{\mathrm{APM}}$ estimate is less sensitive to the existence of sex differences in γ compared to the use of ${\hat{\gamma }}_{\mathrm{meta}}$, and (3) no estimation efficiency is lost by applying the ${\hat{\gamma }}_{APM}$ to the population with larger sample sizes. In real-data analyses, we identified several sex-difference patterns between the causal association of sleep phenotypes and CVD-related outcomes, including OSA on CKD as well as long sleep duration on several CVD-related outcomes, offering potential implications for research in sex-specific cardiovascular medicine. The method itself has broader applicability and could be used to address sex differences for a large set of complex traits. More generally, the method’s utility may extend to other types of stratified, or group-specific, analysis, particularly when some similarities are expected between groups, and beyond causal association analysis. Relevant scenarios are common in both statistical genetics and biomedical research, for example, in improving the predictive performance of polygenic risk score analysis in underrepresented populations by borrowing information from genetic associations in European ancestry individuals,^45,46^ improving the power to detect genetic associations with a rare subtype of a disease, by leveraging genetic associations from a common subtype,^47^ and more broadly, phenotypic or other omics-based characterizations of disease subtypes when sample sizes are limited.

We also applied the proposed framework to the male population, which had a larger sample size in our analysis. The results indicate that less information could be borrowed from the smaller female population. Although the shrinkage approaches did not improve causal effect estimation for the male population in our simulations, using them did not result in a loss of estimation efficiency compared to using the raw $\left({\hat{\gamma }}_{M}\right)$. This supports the usefulness of incorporating shrinkage approaches (especially the adaptive approach) into MR analysis when relevant summary statistics are available, regardless of the corresponding sample size.

Due to the low number of IVs that could be used when applying a genome-wide significance threshold (5 × 10^−8^) for selecting IVs, we considered a lower *p* value threshold ($p\;<\;10^{-5}$), as suggested by Burgess et al.,^27^ as the minimal threshold value for selecting IVs in two-sample MR analysis. However, this strategy may increase the risk of including several weak instruments in MR analysis. Therefore, we applied advanced MR methods that address weak instrumental bias, including MR-RAPS,^30^ cML,^42^ the contaminated mixture model,^41^ and robust MR methods,^40^ in simulations and in real-data analyses. We used MR-RAPS, which demonstrated superior performance in our simulation studies, as the primary method. The consistent findings between MR-RAPS and cML, the top two methods with the best estimation performance in simulation studies, also increase confidence in the data analysis findings. Additionally, to examine the influence of violating the horizontal pleiotropy assumption due to using a lower *p* value threshold to select IVs, we compared the causal effect estimated by MR-RAPS with MR-PRESSO,^44^ another widely used approach developed for detecting horizontal pleiotropy effects, in our primary and secondary analyses. The results from MR-RAPS and MR-PRESSO are highly consistent, increasing the expected reliability of our findings using MR-RAPS.

Sleep traits, here used as exposures, are often correlated with each other,^48^ as well as with other traits. High BMI is a well-known risk factor for some sleep phenotypes, particularly OSA.^49^ However, the direction of causal association is often unclear. Because high BMI is known as an “upstream” risk factor (i.e., a cause) of some of the sleep traits, we performed univariable MR analyses using both BMI-adjusted and BMI-unadjusted GWASs and compared the results as part of our primary analysis, in addition to an MVMR analysis, with BMI incorporated as an exposure, in secondary analysis. There were recent reports in the MR literature that in some cases, applying two-sample MR methodology on summary statistics from a GWAS that adjusted to heritable covariates may induce bias in the estimated causal associations,^50^ depending on the specific causal structure between the exposure, outcome, the covariate, and, possibly, additional unmeasured confounders. Recent papers showed that MVMR may alleviate such bias in some, but not all, settings of causal structures.^51,52^ The overall patterns of our results suggested higher similarity between BMI-unadjusted univariate MR and BMI-adjusted MVMR compared to the BMI-adjusted univariate MR and either unadjusted MR or BMI-adjusted MVMR. Associations tended to be stronger and more statistically significant in the BMI-unadjusted univariate MR and the BMI-adjusted MVMR compared to the BMI-adjusted MR. Notably, in association analyses that use sleep traits as exposure and rely on individual-level data, we often see substantial differences between BMI-adjusted and -unadjusted analyses, including in recent analyses that used the OSA polygenic score in associations with CVD outcomes.^53^ Therefore, it is difficult to determine which analytic approach for BMI adjustment is more reliable, especially given that the underlying causal structure is not entirely clear. Critically, in our MVMR analysis of OSA, the conditional F-statistics after accounting for BMI were all smaller than 10 in both male and female strata. In all, it is important to use these complementary analyses to account for BMI as a common cause or a confounder (it may not be a cause of all sleep traits) of sleep phenotypes and CVD outcomes. Lastly, it is important to acknowledge the potential correlation among sleep traits and the presence of other unmeasured confounders. We chose not to perform additional MVMR analysis due to the unknown causal structure between these traits, and we recommend that readers be mindful of these limitations. Additionally, complementary results from longitudinal and interventional studies are important for making strong conclusions and for ultimately guiding clinical practice.

Motivated by the need to address low female sample size in MVP data and the use of its summary statistics as an exposure GWAS in MR analysis, our focus has been on calibrating the variant-exposure effect estimates. However, we did not apply such calibrations to the outcome GWASs, as the balanced sample sizes across sex-specific GWAS in AoU suggest that further calibrations might not substantially improve the efficiency of sex-specific summary statistics, compared to the exposure GWASs. Simultaneously incorporating the proposed Bayesian framework into exposure and outcome GWASs will be of interest if both GWASs were performed with limited sample sizes.

Some limitations of this work should be discussed. First, the lack of sex-specific sleep GWASs limits the selection of IVs using independent datasets. Instead, we directly utilized the MVP sleep GWASs for the IV selection. Various IV selection strategies were implemented, including sex-specific selection, sex-specific APM estimate selection in primary analysis, and sex-combined selection by using the FE meta estimate in secondary analysis. The results, however, are only sometimes consistent across different selection methods, indicating that the estimation of causal effects is sensitive to the IV selection process. This sensitivity could be due to the strength of the IVs and differences in the underlying genetic architecture captured by the different IVs. A related concern is the limited sample size in the MVP White HARE group (European ancestry) dataset, which likely contributes to the lack of significant findings after multiple testing correction in both sex-specific and sex difference analyses. The statistical power to detect causal effects depends on the exposure GWAS sample size, which is limited in females. Notably, even within the proposed Bayesian framework, the analysis in the female stratum is limited in that information can be borrowed from the male stratum only to the extent that genetic effects appear similar between the two sexes. Thus, null findings may reflect low power. We used the summary statistics from the MVP OSA GWAS to guide the variant-exposure effect estimates in simulations to evaluate sex differences in causal effect; however, the statistical power is low (approximately 0.3–0.4) even when the true sex-specific difference in causal effect was as large as 0.1. While the real-data analysis has a larger sample size, the multiple testing burden is higher (we used a *p* value threshold of 0.05 in the simulations). Further, the simulation results highlight that larger sample sizes are needed for testing for sex differences (likely due to comparison of two estimates, so that the standard error of the test statistics is high), compared to the sample sizes that we ideally need to estimate sex-specific effects with confidence (i.e., with a low enough MSE). These findings suggest that substantially larger sample sizes are necessary to reliably identify differences in causal effects between sexes, especially after correcting for multiple testing.

The second limitation arises from the test of sex differences in exposure-outcome causal effects. Our current framework of shrinkage estimates is developed to improve power for sex-specific estimates but is currently not developed for testing for sex differences. Using these estimates in tests of sex differences may result in correlated causal effect estimates between sexes and, potentially, reduced power, given that information is borrowed between sex strata. A test of sex differences that includes a covariance correction between the shrinkage estimates^32^ can be exploited. However, quantifying the covariance between causal estimates across sexes using shrinkage approaches is challenging and a topic of future work. Therefore, we included only the causal estimates from the raw estimates (${\hat{\gamma }}_{F,\mathrm{raw}}$ and ${\hat{\gamma }}_{M,\mathrm{raw}}$) in our sex differences test, where we applied the conventional two-sample t test, performed under the assumption of independence between the two compared samples.

We used only European ancestry individuals due to the larger sample sizes available. Assessing the transferability of our findings to other populations, and potentially applying a similar shrinkage framework to improve IVs in non-European ancestry populations, is of interest for future research.

While the APM method substantially improves the statistical reliability of female-specific MR analyses by stabilizing weak instruments, it does not address the possibility that different biological pathways underlie the same disease phenotype across sexes. For example, there is emerging evidence that the pathophysiology of OSA in women differs in some respects from that of OSA in men, with a lower severity in women of the physiologic endotypes causing sleep apnea, due in part to the effect of sex hormones on ventilatory drive, resulting in fewer discrete respiratory events, although often with more prolonged “shallow” respiratory event.^6,7,54^ Differences in pathophysiology may be related to differences in causal effects. Thus, interpretation of causal effects in general and of those estimated via the APM framework should carefully consider potential differences in the pathophysiology of exposure traits and the potential implication of applying a Bayesian shrinkage framework when pathophysiological sex differences may be strong. Differences in pathophysiology by sex can also lead to differences in causal effect estimates when sex differences in prevalence of an outcome are small. Interpretation of such sex differences should acknowledge the potential overlap between sleep measures and other confounding factors.

## Supplementary Material

MMC2

MMC4

MMC3

MMC1

Supplemental information

Supplemental information can be found online at https://doi.org/10.1016/j.ajhg.2025.07.015.

## Figures and Tables

**Table 1.** The proposed Bayesian framework for calibrating variant-trait effect size estimates

Likelihood	Prior distribution of $\gamma_{jF}$	Posterior mean of $\gamma_{jF}$	Estimator name
${\overset{ˆ}{\gamma }}_{jF,\mathrm{raw}}\sim N\left(\gamma_{jF},{\overset{ˆ}{\sigma }}_{\gamma ,jF,\mathrm{raw}}^{2}\right)$	$\gamma_{jF}\sim N\left({\overset{ˆ}{\gamma }}_{jM,\mathrm{raw}},{\overset{ˆ}{\sigma }}_{\gamma j,M,\mathrm{raw}}^{2}\right)$	$\frac{{\overset{ˆ}{\sigma }}_{\gamma ,jM,\mathrm{raw}}^{2}}{{\overset{ˆ}{\sigma }}_{\gamma ,jF,\mathrm{raw}}^{2}\;+\;{\overset{ˆ}{\sigma }}_{\gamma ,jM,\mathrm{raw}}^{2}}{\overset{ˆ}{\gamma }}_{jF,\mathrm{raw}}+\frac{{\overset{ˆ}{\sigma }}_{\gamma ,jF,\mathrm{raw}}^{2}}{{\overset{ˆ}{\sigma }}_{\gamma ,jF,\mathrm{raw}}^{2}\;+\;{\overset{ˆ}{\sigma }}_{\gamma ,jM,\mathrm{raw}}^{2}}{\overset{ˆ}{\gamma }}_{jM\mathrm{,raw}}$	FE meta estimate, ${\overset{ˆ}{\gamma }}_{j,\mathrm{meta}}$
	$\gamma_{jF}\sim N({\overset{ˆ}{\gamma }}_{j,\mathrm{meta}},{\left({\overset{ˆ}{\gamma }}_{jF,\mathrm{raw}}-{\overset{ˆ}{\gamma }}_{j_{M,\mathrm{raw}}}\right)}^{2}+{\overset{ˆ}{\sigma }}_{\gamma ,\mathrm{jM,raw}}^{2})$	$\frac{{\left({\overset{ˆ}{\gamma }}_{jF,\mathrm{naw}}\;-\;{\overset{ˆ}{\gamma }}_{jM,\mathrm{raw}}\right)}^{2}\;+\;{\overset{ˆ}{\sigma }}_{\gamma ,jM,\mathrm{raw}}^{2}}{{\left({\overset{ˆ}{\gamma }}_{jF,\mathrm{raw}}\;-\;{\overset{ˆ}{\gamma }}_{jM,\mathrm{raw}}\right)}^{2}\;+\;{\overset{ˆ}{\sigma }}_{\gamma ,jM,\mathrm{raw}}^{2}\;+\;{\overset{ˆ}{\sigma }}_{\gamma jF,\mathrm{raw}}^{2}}{\overset{ˆ}{\gamma }}_{jF,\mathrm{raw}}\;+\;\frac{{\overset{ˆ}{\sigma }}_{\gamma ,jF,\mathrm{raw}}^{2}}{{\left({\overset{ˆ}{\gamma }}_{jF,\mathrm{raw}}\;-\;{\overset{ˆ}{\gamma }}_{jM,\mathrm{raw}}\right)}^{2}\;+\;{\overset{ˆ}{\sigma }}_{\gamma ,jM,\mathrm{raw}}^{2}\;+\;{\overset{ˆ}{\sigma }}_{\gamma ,jF,\mathrm{raw}}^{2}}-{\overset{ˆ}{\gamma }}_{j,\mathrm{meta}}$	APM estimate, ${\overset{ˆ}{\gamma }}_{jF,APM}$

The table summarizes the semi-empirical Bayesian model parameters behind the two proposed variant-trait effect size estimators. The raw effect size estimates are obtained from an analysis of a single stratum (here we focus on the female stratum). By specifying prior distributions on the variant-trait effect sizes (second column), the raw estimates are shrunk toward the prior means to become the estimates provided in the posterior mean column. The last column, estimator name, provides the name and notation of the resulting estimators (FE meta estimate and adaptive posterior mean [APM] estimate).

**Table 2.** Two-sample MR methods used in both simulation studies and real-data analyses

MR method	Description	Software used	Reference
MR-RAPS (primary analysis)	estimation: adjusted profile likelihood estimation with down-weighting of outliers via a robust loss function assumptions: InSIDE, pleiotropic effects follow normal distribution with mean zero	R package: mr.raps R function: mr.raps.overdispersed.robust()	Zhao et al. ^30^
IVW	estimation: aggregate multiple Wald ratio estimates using fixed effect inverse-variance weighting assumptions: InSIDE, zero average pleiotropy effect	R package: MendelianRandomization R function: mr_allmethods()	Burgess et al. ^27^
Constrained ML	estimation: maximum likelihood estimation with a constraint on the number of invalid IVs assumptions: plurality valid	R package: MendelianRandomization R function: mr_cML()	Xue et al. ^42^
Contamination mixture	estimation: profile likelihood estimation assuming that the ratio estimates follow two normal distributions for valid and invalid IVs, respectively assumption: plurality valid	R package: MendelianRandomization R function: mr_conmix()	Burgess et al. ^41^
Weighted median	estimation: compute the median of the causal effect estimated from multiple IVs, weighted by the inverse of the estimate’s sampling variance assumption: majority valid	R package: Mendelian Randomization mr_allmethods()	Bowden et al. ^43^
MR Egger	estimation: weighted meta-regression with an intercept term to capture the average horizontal pleiotropy effect assumption: InSIDE	R package: MendelianRandomization R function: mr _allmethods()	Bowden et al. ^39^
Robust IVW and MR-Egger	estimation: apply robust regression to down-weight or exclude variants with heterogeneous causal estimates	R package: MendelianRandomization R function: mr _allmethods()	Rees et al. ^40^

Summary of the two-sample MR methods used in the simulation studies and real-data analyses. The names of the methods are listed in the first column. A brief description of the estimation approach and the underlying assumptions for each method are provided in the second column. The corresponding software for applying these methods is listed in the third column, and the references are provided in the last column. InSIDE, instrument strength independent of direct effect.

**Table 3.** Top findings from sex-differences test in causal effect estimation

Exposure	Outcome	${\hat{\beta }}_{\mathrm{F,APM}}$	${\hat{\beta }}_{M,APM}$	${\hat{\beta }}_{\mathrm{F,raw}}-{\hat{\beta }}_{\mathrm{M,raw}}$	Sex difference *p* value	Sex difference FDR *p* value
${\hat{\gamma }}_{\mathrm{raw}}$ **selection BMI-unadj**						
OSA	CKD	−0.108 (−0.277, 0.060)	0.171 (−0.015, 0.357)	−0.275 (−0.521, −0.029)	0.028	0.500
${\overset{ˆ}{\gamma }}_{APM}$ **selection BMI-unadj**						
Long sleep	CAD	0.112 (0.001, 0.223)	−0.219 (−0.445, 0.008)	0.328 (0.078, 0.578)	0.009	0.118
Long sleep	CKD	0.049 (−0.046, 0.146)	−0.267 (−0.489, −0.044)	0.317 (0.073, 0.560)	0.011	0.118
Long sleep	HTN	0.035 (−0.003, 0.072)	−0.095 (−0.188, −0.002)	0.128 (0.028, 0.228)	0.011	0.118
Long sleep	T2DM	0.101 (−0.022, 0.225)	−0.125 (−0.299, 0.049)	0.223 (0.014, 0.433)	0.036	0.273
${\hat{\gamma }}_{APM}$ **selection BMI-adj**						
Long sleep	CKD	0.070 (−0.027, 0.168)	−0.202 (−0.425, 0.022)	0.272 (0.028, 0.516)	0.029	0.407
Long sleep	CAD	0.099 (−0.011, 0.209)	−0.146 (−0.341, 0.049)	0.241 (0.019, 0.463)	0.033	0.407
Long sleep	HTN	0.043 (0.004, 0.082)	−0.059 (−0.148, 0.029)	0.101 (0.004, 0.197)	0.041	0.407

Selected findings from tests of sex differences in causal effects. The first column describes the IV selection procedure and whether the exposure GWAS was adjusted for BMI or not. The exposure and outcome of interest are provided in the second and third columns, respectively. Sex-specific causal estimates obtained using MR-RAPS with APM variant-exposure estimates (${\hat{\gamma }}_{APM}$), along with their corresponding 95% confidence intervals, are displayed in the fourth (female-specific) and fifth (male-specific) columns. The estimated causal effects are reported on the log scale, as used in the test of sex differences. The sixth column provides the estimated sex differences in causal effects, calculated from raw variant-exposure estimates (${\hat{\gamma }}_{\mathrm{raw}}$), with 95% confidence intervals. *p* values and FDR-adjusted *p* values for the statistical tests of sex differences are shown in the seventh and eighth columns. The FDR *p* values were computed using the Benjamini-Hochberg procedure. All the sex difference tests are significant in the nominal threshold (p<0.05). IV, instrumental variable; GWAS, genome-wide association study; BMI, body mass index; BMI-unadj, BMI unadjusted; BMI-adj, BMI adjusted; MR-RAPS, MR using robust adjusted profile score method; APM, adaptive posterior mean; OR, odds ratio; OSA, obstructive sleep apnea; CAD, coronary artery disease; CKD, chronic kidney disease; HTN, hypertension; T2DM, type 2 diabetes mellitus; FDR p value, false discovery rate-adjusted *p* value.

**Table 4.** Top findings from sex-specific exposure-outcome causal effect estimation

Exposure	Outcome	BMI adjustment	$exp\{{\overset{ˆ}{\beta }}_{F,APM}\}$	$exp\{{\overset{ˆ}{\beta }}_{M,APM}\}$
${\hat{\gamma }}_{\mathrm{raw}}$ **selection**				
Insomnia	CKD	unadjusted	**1.23 (1.01, 1.49)**	1.23 (0.88, 1.73)
Long sleep	HTN	unadjusted	**1.03 (1.00, 1.06)**	0.94 (0.85, 1.05)
Short sleep	CAD	unadjusted	1.01 (0.85, 1.21)	**1.32 (1.03, 1.69)**
${\overset{ˆ}{\gamma }}_{APM}$ **selection**				
OSA	CKD	unadjusted	0.95 (0.77, 1.16)	**1.23 (1.03, 1.47)**
OSA	HF	unadjusted	1.07 (0.86, 1.35)	**1.30 (1.04, 1.63)**
OSA	T2DM	unadjusted	**1.32 (1.05, 1.66)**	1.09 (0.92, 1.28)
Sleepiness	AF	unadjusted	**1.18 (1.00, 1.38)**	0.99 (0.78, 1.27)
Long sleep	CAD	unadjusted	**1.11 (1.00, 1.25)**	0.80 (0.64, 1.00)
Long sleep	CKD	unadjusted	1.05 (0.95, 1.16)	**0.77 (0.61, 0.96)**
Long sleep	HTN	unadjusted	1.04 (0.99, 1.07)	**0.91 (0.83, 0.99)**
OSA	CKD	adjusted	1.09 (0.86, 1.38)	**1.27 (1.01, 1.59)**
OSA	HTN	adjusted	**1.14 (1.03, 1.25)**	0.99 (0.87, 1.14)
Insomnia	CKD	adjusted	**1.38 (1.07, 1.79)**	1.19 (0.88, 1.61)
Long sleep	HTN	adjusted	**1.04 (1.00, 1.09)**	0.94 (0.86, 1.03)

Selected findings from sex-specific exposure-outcome causal effect estimation, where significant results were identified in at least one sex-stratum, using MR-RAPS with APM variant-exposure estimates (${\overset{ˆ}{\gamma }}_{\mathrm{APM}}$). The results based on ${\overset{ˆ}{\gamma }}_{\mathrm{raw}}$ for IV selection are shown in the top three rows, while the remaining results are from the analyses using ${\hat{\gamma }}_{\mathrm{APM}}$ for IV selection. The exposure and outcome of interest are listed in the second and third columns, respectively. The fourth column indicates whether BMI adjustment was applied in the variant-phenotype association analysis. The estimated causal effects are given in an OR scale (exp{β}). The female-specific causal estimates and the corresponding 95% confidence intervals are shown in the fifth column, and the results for males are in the sixth column. Bold font indicates statistically significant results (p<0.05). MR-RAPS, MR using robust adjusted profile score method; APM, adaptive posterior mean; IV, instrumental variable; OR, odds ratio; OSA, obstructive sleep apnea; AF, atrial fibrillation; CAD, coronary artery disease; CKD, chronic kidney disease; HF, heart failure; HTN, hypertension; T2DM, type 2 diabetes mellitus; BMI, body mass index.

## Data Availability

Summary statistics from sex-specific sleep trait GWASs will become available on the dbGaP repository “Veterans Administration (VA) MVP Summary Results from Omics Studies,” study accession phs001672. The R code used to implement the proposed semi-empirical Bayes framework, two-sample MR approaches, and simulation studies is available on the GitHub repository: https://github.com/Gene-Huang/sex-specific-MR. The harmonized summary statistics for both exposure and outcome GWASs used in our data analysis are also provided in the GitHub repository.

## Appendix A

### F-statistic for IV strength quantification

We computed the F-statistic in both simulation studies and real-data analysis to quantify IV strength. For each variant, the F-statistic was computed as $F=\frac{{\hat{\gamma }}^{2}}{{\hat{\sigma }}_{\gamma }^{2}}$. The average F-statistics across all selected variants was then computed as $\overset{‾}{F}=\frac{1}{p}\sum_{j=1}^{p}\frac{{\hat{\gamma }}_{j}^{2}}{{\hat{\sigma }}_{\gamma ,j}^{2}}$, where p denotes the total number of IVs. We also compute the F-statistic using ${\hat{\gamma }}_{APM}$ and its corresponding standard error estimates for comparison. The average F-statistics from the simulation studies is presented in Table S3. For the real-data analyses, Table S5 reports the average F-statistics for each sleep exposure. Variant-level F-statistic for each exposure-outcome analysis was also computed and is available in our publicly accessible repository: https://github.com/Gene-Huang/sex-specific-MR/tree/main/harmonized_exposure_outcome_sumstat/.

### Sex-specific sleep GWASs in MVP

We performed sex-specific GWASs for four sleep phenotypes (insomnia, long sleep duration, short sleep duration, and excessive daytime sleepiness) and used GWAS summary statistics from previously published GWASs of OSA^16^ using MVP participants. Sleep phenotypes are described below. We only used the European (White) harmonized race/ethnicity and genetic ancestry (HARE) group to match the genetic ancestry of the available outcome sex-stratified GWASs from the AoU. We removed related individuals based on kinship coefficients ≥ 0.0884, where the kinship coefficients were estimated using KING v.2.0,^55^ so that all analyses were conducted using an unrelated set of individuals (including participants from across the female and male strata that were unrelated). Sex chromosome checks confirmed biological sex. Variants were filtered based on imputation quality, requiring an INFO score of at least $R^{2}\geq 0.6$ and a minor-allele frequency ≥ 0.01. Across analyses, sample sizes ranged from ~15,000 to 30,000 (female stratum) and from ~204,000 to 380,000 (male stratum). All analyses were adjusted for age and the first 10 principal components (PCs) of genetic data. For each trait, two GWASs were performed: one with and one without BMI adjustment. BMI-adjusted models had BMI as a covariate using both linear and squared terms. Analyses were performed separately using PLINK v.2.00a3LM^56^ in each sex stratum. The summary of MVP sleep GWAS results is shown in Note S5.

### Sleep phenotypes

Sleep phenotypes were defined as previously reported.^16^ In detail, OSA was defined based on the VA electronic health record using a multimodal automated phenotyping (MAP) algorithm.^57^ The MAP algorithm was applied to predict general sleep apnea and OSA separately. For each trait, it resulted in a score, roughly mapping to a likelihood of having that condition. The final OSA definition combined both scores. Insomnia status was inferred using a MAP algorithm applied to relevant ICD codes and elements extracted using natural language processing. Insomnia status was inferred at the same age as was used in the OSA analysis, i.e., a participant was considered to have insomnia if their age of first insomnia ICD code was the same or earlier than their age in the OSA analysis. All other sleep phenotypes were self-reported based on the baseline questionnaire administered to program participants. Long sleep was defined as sleep durations >9 h, and short sleep as sleep durations <6 h, where the sleep duration was defined based on the response to the question about hours of sleep in a typical day, with responses ranging from “5 or less” to “10 or more,” with increments of 0.5. The excessive daytime sleepiness phenotype was based on the questionnaire item “feeling excessively sleepy during the day (does not include regular naps),” with yes or no answers. All five sleep phenotypes are binary.

### MVP ethics statement

MVP received ethical/study protocol approval from the VA Central Institutional Review Board, and written informed consent was obtained for all participants.

### Association analysis with CVD-related outcomes in AoU

We used short-read whole-genome sequencing (srWGS) data (version 7) from the AoU study to conduct association analysis with CVD-related outcomes. To reduce memory storage requirements, we focused on genomics data pre-filtered by the following criteria: a population-specific allele frequency ≥ 1% or a population-specific allele count > 100 (data are accessed via the AoU online platform using gs://fc-aou-datasets-controlled/v7/wgs/short_read/snpindel/acaf_threshold_v7.1). To align with summary statistics computed from the White HARE group in the MVP, we included only White individuals (as determined by self-reported race and ethnicity) in the AoU analysis. Related individuals were excluded based on information available via the online platform in gs://fcaou-datasets-controlled/v7/wgs/short_read/snpindel/aux/relatedness/relatedness_flagged_samples.tsv. We also restricted the analysis to adults aged 18 to 95, with BMI values ranging from 17 to 55. Following data preprocessing, approximately 114,000 White individuals were included, consisting of 67,600 females and 46,400 males. The exact sample size varied slightly depending on the phenotype analyzed. Six binary CVD-related phenotypes were considered: atrial fibrillation (AF), coronary artery disease (CAD), cardiovascular disease (CVD), heart failure (HF), hypertension (HTN), and type 2 diabetes mellitus (T2DM). Detailed selection criteria for these clinical outcomes and the corresponding SNOMED codes and OMOP Concept IDs in the AoU study are provided in Table S7. The characteristics of AoU participants in the variant-phenotype association analysis are summarized in Table S8. More details are summarized in Note S4.

After matching the variants identified as IVs according to the exposure dataset (MVP), we conducted single-variant association analyses with the six binary outcomes. We used logistic regression, adjusting for age and 16 genetic PCs as covariates in the BMI-unadjusted analyses. For the BMI-adjusted analyses, the models were further adjusted for BMI, including both linear and quadratic terms. The effect sizes of the variants, i.e., log (OR) with the corresponding SD, were then extracted for use as summary statistics in the outcome GWAS.

### MVMR analysis

We performed MVMR to estimate the direct effect of each sleep phenotype on CVD outcomes while adjusting for BMI as a secondary exposure. For both the sleep and CVD traits, we used effect estimates derived from BMI-unadjusted variant-trait associations. BMI summary statistics were downloaded from a large-scale meta-analysis of approximately 700,000 individuals of European ancestry, combining data from the GIANT consortium and the UK Biobank.^58^ As sleep phenotypes were considered the primary exposures of interest, we retained the same list of IVs used in the primary analysis and extracted the corresponding BMI effect estimates for the same set of variants. For the data harmonization, we first aligned effect estimates for sleep phenotypes and BMI using the harmonise_data() function from the TwoSampleMR R package (v.0.6.8), ensuring consistency in the effect allele across exposures. Next, we harmonized both exposures with the CVD outcome using the mv_harmonise_data() function from the same package to align all effect estimates to the same effect allele. MVMR was conducted using the robust multivariable inverse-variance weighted (MV-IVW) method,^59^ and the average conditional F-statistics were computed using the strength_mvmr() function from the MVMR R package (v.0.4.1^60^).

### Simulation studies

We performed simulation studies to evaluate the performance of exposure-outcome causal effect ($\beta_{F}$ and $\beta_{M}$) estimation, using shrinkage variant-exposure effect estimates (${\hat{\gamma }}_{\mathrm{meta}}$ and ${\hat{\gamma }}_{APM}$) as input to existing two-sample MR approaches. We then compared these to the use of uncalibrated (raw) effect estimates (${\hat{\gamma }}_{\mathrm{raw}}$). We employed three settings to simulate sex-specific variant-exposure effect sizes ($\gamma_{F}$ and $\gamma_{M}$) and considered different levels of sex difference in causal effect within each setting.

### Simulation data generation

We simulated data via the structural model presented in Figure 1A, where we use S (for sleep phenotype) to represent the exposure variable and C (for CVD phenotype) to represent the outcome variable. We first generated individual-level genetic data for two datasets, corresponding to two populations used in an exposure GWAS (one population) and an outcome GWAS (second population). In each of the datasets, the data-generating process was carried out independently in the females and males. We set the female sample size of the exposure dataset to $N_{F}^{S}=2,000$ and the sample size of the male stratum to $N_{M}^{S}=20,\;000$, matching the sex sample size proportions in the MVP. In the outcome dataset, the female and male sample sizes were set to be the same: $N_{F}^{C}=N_{M}^{C}=10,\;000$. For each genetic variant, allele counts were generated independently from Binomial(2,0.3) distribution, assuming that all variants are in linkage equilibrium with allele frequencies of 0.3.

In the exposure dataset, we generated the exposure variable according to the following linear model:

(Equation A1)
$$S_{Fi}=1+\sum_{j=1}^{p}\gamma_{jF}\times g_{Fji}+\alpha \times U_{Fi}+\varepsilon_{Fi},i=1,\;2,\;\ldots ,N_{F}^{S}\;\text{and}$$

(Equation A2)
$$S_{Mi}=1+\sum_{j=1}^{p}\gamma_{Mj}\times g_{Mji}+\alpha \times U_{Mi}+\varepsilon_{Mi},i=1,\;2,\;\ldots ,N_{M}^{S},$$

with p=100 independent variants. U represents the unknown confounder, which we generated from normal distribution with mean 0 but different variances in female and male populations: $U_{Fi}\sim N(0,\;1)$ and $U_{Mi}\sim N(0,\;0.5)$. The random errors $\varepsilon_{Fi}$ and $\varepsilon_{Mi}$ were generated from standard normal distribution for both the female and male populations. The $\gamma_{jF}$ and $\gamma_{Mj}$ are the underlying sex-specific variant-exposure effect sizes. The effect size of the unknown confounder to the exposure association was set as α=0.1 for both the female and male populations.

Next, we use the same strategy to generate the genetic data, exposure variable, and unmeasured confounder for the outcome population,

(Equation A3)
$$S_{Fi}^{O}=1+\sum_{j=1}^{p}\gamma_{jF}\times g_{Fji}^{O}+\alpha \times U_{Fi}^{O}+\varepsilon_{Fi}^{O},i=1,\;2,\;\ldots ,N_{F}^{C}\;\text{and}$$

(Equation A4)
$$S_{Mi}^{O}=1+\sum_{j=1}^{p}\gamma_{Mj}\times g_{Mji}^{O}+\alpha \times U_{Mi}^{O}+\varepsilon_{Mi}^{O},i=1,\;2,\;\ldots ,N_{M}^{C}.$$

The superscript O is used to distinguish the exposure simulated in the outcome population from the exposure simulated in the exposure population. Based on the generated exposure variables $S_{Fi}^{O}$ and $S_{Mi}^{O}$, we then generate the outcome variable through the following linear equation:

(Equation A5)
$$C_{Fi}=1+\beta_{F}\times S_{Fi}^{O}+\phi \times U_{Fi}^{O}+\varepsilon_{Fi}^{C},i=1,\;2,\;\ldots ,N_{F}^{C}\;\text{and}$$

(Equation A6)
$$C_{Mi}=1+\beta_{M}\times S_{Mi}^{O}+\phi \times U_{Mi}^{O}+\varepsilon_{Mi}^{C},i=1,\;2,\;\ldots ,N_{M}^{C},$$

where ϕ denotes the effect sizes of the unknown confounder on the outcome, which we set to ϕ=0.1. The $\beta_{F}$ and $\beta_{M}$ are the underlying true female and male exposure-outcome causal effects, respectively.

After generating the individual-level data, we get the exposure GWAS and outcome GWAS summary statistics by fitting marginal linear regression models. The exposure data summary statistics $\left({\hat{\gamma }}_{jF},{\hat{\sigma }}_{\gamma ,jF}^{2}\right)$ and $\left({\hat{\gamma }}_{jM},{\hat{\sigma }}_{\gamma ,jM}^{2}\right)$, j=1,2,…,100 are obtained by fitting the following regression using the data generated from Equations A1 and A2:

$$E\left(S_{F}\right)={\tilde{\gamma }}_{F0}+{\tilde{\gamma }}_{jF}\times g_{Fj},j=1,\;2,\;\ldots ,100\;\text{and}$$

$$E\left(S_{M}\right)={\tilde{\gamma }}_{M0}+{\tilde{\gamma }}_{jM}\times g_{Mj},j=1,\;2,\;\ldots ,100,$$

and the same procedure is applied for the outcome population

$$E\left(C_{F}\right)=\Gamma_{F0}+\Gamma_{Fj}\times g_{Fj}^{O},j=1,\;2,\;\ldots ,100\;\text{and}$$

$$E\left(C_{M}\right)=\Gamma_{M0}+\Gamma_{Mj}\times g_{Mj}^{O},j=1,\;2,\ldots ,100$$

The outcome GWAS summary statistics are (${\hat{\Gamma }}_{jF},{\hat{\sigma }}_{\Gamma ,jF}^{2}$) and $\left({\hat{\Gamma }}_{jM},\;{\hat{\sigma }}_{\Gamma ,jM}^{2}\right)$, j=1,2,…,100.

We performed simulations in three simulation settings that differed in the way that variant-exposure effect sizes were simulated. In all settings, we considered a few models for the expo-sure-outcome effect sizes: no sex differences in exposure-outcome causal effects, with $\beta_{F}=\beta_{M}=0.1$, and sex differences in expo-sure-outcome causal effect with $\beta_{M}=0.1$, and $\beta_{F}\in \{0,\;0.05,\;0.15\}$.

### Simulation settings 1: Fixed sex differences in variant-exposure effects

Here, we simulated from settings in which the sex differences in $\gamma_{F}$ and $\gamma_{M}$, when there were such differences, were weak and fixed across variants. Male-specific variant-exposure effect sizes were $\gamma_{jM}=0.1$, j=1,2,…,100 for all variants. For the female population, some variants had the same effect size as in the male population $\left(\gamma_{jF}=0.1\right)$, and a subset of variants (sized 10, 50, or 90 out of 100) had female-specific variant-exposure effect size $\gamma_{jF}=0.05$. For the sex differences in the causal effect setting, we fixed 50% of variants to have sex differences in variant-exposure effect sizes.

### Simulation settings 2: Random sex differences in variant-exposure effects

Here, we simulated from settings in which the sex differences in variant-exposure effect sizes, when there were such differences, were strong. For each variant j=1,2,…,100, we generated a pair of female and male variant-exposure effect sizes from a bivariate normal distribution γjFγjM~N20.10.1,Σ=0.01ϕϕ0.01, where we considered different covariance value ϕ to set the correlations $\left(\frac{\phi }{0.01}\right)$ between $\left(\gamma_{F},\gamma_{M}\right)$ to equal 0.5, 0.7, or 0.9 when the exposure-outcome causal effect was the same in males and females ($\beta_{F}=\beta_{M}=0.1$). When $\beta_{M}\neq \beta_{F}$, the correlation between ($\gamma_{F},\gamma_{M}$) was set to 0.7. Finally, the variant exposure effect sizes $\gamma_{F}$, $\gamma_{M}$ were randomly generated in each simulation replicate.

### Simulation settings 3: Summary statistics from MVP OSA GWAS guide variant-exposure effect sizes

Here, we used the summary statistics from the MVP OSA GWAS (without BMI adjustment) to guide the $\gamma_{F}$ and $\gamma_{M}$ values. First, we conducted *p* value thresholding and clumping for the male-specific summary statistics. The *p* value threshold was set at 10^−5^, with a clumping window of 10,000 kb and a correlation threshold of 0.001, using the European population reference panel from 1000 Genomes. This resulted in a list of 110 variants. We randomly selected 100 variants used their male- and female-specific effect size estimates from the MVP GWAS in simulations.

### Two-sample MR methods

We applied the following two-sample MR methods, described in Table 2, over the raw and calibrated estimated variant effect sizes.
