# Supplementary material for: A semi-empirical Bayes approach for calibrating weak instrumental bias in sex-specific Mendelian randomization studies
Source: Am J Hum Genet. 2025 Sep 4;112(9):2213–31. doi: 10.1016/j.ajhg.2025.07.015 (PMC12416758; doi:10.1016/j.ajhg.2025.07.015)
Supplement: Document S2. Article plus supplemental information [file mmc5.pdf]

A semi-empirical Bayes approach for calibrating weak instrumental bias in sex-specific Mendelian randomization studies

Authors

Yu-Jyun Huang, Nuzulul Kurniansyah, Daniel F. Levey, ..., Daniel J. Gottlieb, Kenneth M. Rice, Tamar Sofer

Correspondence

tsofer@bidmc.harvard.edu

This article proposes a semi-empirical Bayesian framework that borrows information across populations to improve estimation in strata with limited sample sizes. The method is applied to sex-specific Mendelian randomization studies estimating the causal relationship between sleep phenotypes and cardiovascular-related diseases. This framework has broad applicability to multi-stratum analyses.

Huang et al., 2025, The American Journal of Human Genetics *112*, 2213–2231
September 4, 2025

© 2025 The Author(s). Published by Elsevier Inc. on behalf of American Society of Human Genetics.
https://doi.org/10.1016/j.ajhg.2025.07.015

Check for updates

CellPress

A semi-empirical Bayes approach for calibrating weak instrumental bias in sex-specific Mendelian randomization studies

Yu-Jyun Huang,[1,2] Nuzulul Kurniansyah,[3] Daniel F. Levey,[4,5] Joel Gelernter,[4,5] Jennifer E. Huffman,[6,7,8] Kelly Cho,[6,9] Peter W.F. Wilson,[10] Daniel J. Gottlieb,[3,6] Kenneth M. Rice,[11] and Tamar Sofer[1,2,3,12,*]

Summary

Strong sex differences exist in sleep phenotypes and also cardiovascular diseases (CVDs). However, sex-specific causal effects of sleep phenotypes on CVD-related outcomes have not been thoroughly examined. Mendelian randomization (MR) analysis is a useful approach for estimating the causal effect of a risk factor on an outcome of interest when interventional studies are not available. We first conducted sex-specific genome-wide association studies (GWASs) for suboptimal-sleep phenotypes (insomnia, obstructive sleep apnea [OSA], short and long sleep durations, and excessive daytime sleepiness) utilizing the Million Veteran Program (MVP) dataset. We then developed a semi-empirical Bayesian framework that (1) calibrates variant-phenotype effect estimates by leveraging information across sex groups and (2) applies shrinkage sex-specific effect estimates in MR analysis to alleviate weak instrumental bias when sex groups are analyzed in isolation. Simulation studies demonstrate that the causal effect estimates derived from our framework are substantially more efficient than those obtained through conventional methods. We estimated the causal effects of sleep phenotypes on CVD-related outcomes using sex-specific GWAS data from the MVP and All of Us. Significant sex differences in causal effects were observed, particularly between OSA and chronic kidney disease, as well as long sleep duration on several CVD-related outcomes. By applying shrinkage estimates for instrumental variable selection, we identified multiple sex-specific significant causal relationships between OSA and CVD-related phenotypes. The method is generalizable and can be used to improve power and alleviate weak instrument bias when only a small sample is available for a specific condition or group.

Introduction

Investigating sex differences in health and disease mechanisms is a leading public health research priority.[1–3] Sex differences are evident in various health conditions, including suboptimal-sleep phenotypes and cardiovascular diseases (CVDs). For example, there is a higher prevalence of insomnia in women,[4,5] whereas obstructive sleep apnea (OSA) is more common in men.[6,7] Cardiovascular-related diseases, such as myocardial infarction and hypertension (HTN), generally present with a higher incidence in male adults compared to females.[8–10] Increasing numbers of genome-wide association studies (GWASs), which, like other genomic studies, often analyze only autosomal chromosomes, have identified strong signals of sex differences.[11–13] Examples include, but are not limited to, sex differences in genetic variant effect sizes,[14–16] sex-specific genetic risk associations,[17–19] and sex-biased gene/protein expression level.[20–22] Sex-specific causal effects of modifiable risk exposures on outcomes can, under some conditions, be obtained via Mendelian randomization (MR) analysis.[23] But because of the paucity of sex-specific interventional studies, or studies with sufficient sex-stratified sample sizes, much remains unknown about how sex-specific causal effects may inform targeted disease treatments or interventions, ultimately limiting efforts to reduce sex disparities in health.[24–26]

MR analysis is widely used in genetic epidemiology because it can, using GWAS summary statistics alone,[27–29] estimate causal effects from observational data. Sex-specific MR analysis, however, is limited in comparison: not only is each sex group's sample size smaller than the total in any one study, but GWAS reporting is not always sex specific. This problem can be worse where GWAS participation is biased for structural or other reasons. For example, in the Million Veteran Program (MVP) of the US Department of Veterans Affairs (VA) healthcare system, which collected genetic data and extensive phenotypes from US veterans, only 10% of participants are female, in line with the proportion of female veterans. In sex-specific MR analysis, the small sample size of the female MVP population particularly limits the strength of instrumental variables (IVs) identified from female-specific GWASs, which may make causal effect estimates unstable due to the corresponding weak IV bias.[30,31]

[1]Department of Medicine, Harvard Medical School, Boston, MA, USA; [2]CardioVascular Institute (CVI), Beth Israel Deaconess Medical Center, Boston, MA, USA; [3]Department of Medicine, Brigham and Women's Hospital, Boston, MA, USA; [4]Division of Human Genetics, Department of Psychiatry, Yale University School of Medicine, New Haven, CT, USA; [5]Department of Psychiatry, Veterans Affairs Connecticut Healthcare Center, West Haven, CT, USA; [6]Massachusetts Veterans Epidemiology Research and Information Center, VA Healthcare System, Boston, MA, USA; [7]VA Palo Alto Health Care System, Palo Alto, CA, USA; [8]Palo Alto Veterans Institute for Research, Palo Alto, CA, USA; [9]Division of Aging, Department of Medicine, Brigham and Women's Hospital and Harvard Medical School, Boston, MA, USA; [10]Atlanta VA Healthcare System, Decatur, GA, USA; [11]Department of Biostatistics, University of Washington, Seattle, WA, USA; [12]Department of Biostatistics, Harvard T.H. Chan School of Public Health, Boston, MA, USA
*Correspondence: tsofer@bidmc.harvard.edu
https://doi.org/10.1016/j.ajhg.2025.07.015.
© 2025 The Author(s). Published by Elsevier Inc. on behalf of American Society of Human Genetics.
This is an open access article under the CC BY license (http://creativecommons.org/licenses/by/4.0/).

To address these challenges, we (1) performed sex-specific GWASs of sleep phenotypes in the MVP, (2) developed a novel statistical approach to enhance the precision of sex-specific variant-phenotype effect estimates by leveraging information across sex groups, and (3) integrated our new shrinkage estimator into MR analyses to improve causal effect estimates, particularly for the sex group with smaller sample sizes. Motivation for this approach comes from recent findings of the high correlation between the genetic components of females and males of multiple traits,[32,33] suggesting that many (though not all) variant associations are similar between sexes. Thus, focusing on the MVP, where the female population is small, our approach is to borrow information from the male population in an adaptive manner to improve female-specific variant effect estimates and, ultimately, exposure-outcome causal effect estimates.

The proposed approach, incorporating the spirit of both transfer learning and empirical Bayes, uses male-specific effect size estimates to specify prior distributions on the female-specific variant-exposure effect sizes (i.e., using information from the larger sample to improve power in the smaller). The inverse variance-weighted meta-analysis estimator and the adaptive weight (AW) estimator (proposed for analyzing secondary outcomes in case-control studies[34–36]) can both be derived as the posterior mean in the proposed framework. In simulation studies, compared to the standard use of variant-exposure summary statistics in MR analysis, our approach improves the efficiency of exposure-outcome causal effect estimates. Finally, using sex-specific data from the MVP, along with genetic association results from the All of Us (AoU) study, we applied a two-sample MR approach to estimate the causal effects of sleep phenotypes on CVD-related outcomes. Our method identified several sex-specific causal associations. Specifically, insomnia was causally associated with an increased risk of chronic kidney disease (CKD) in females, long sleep was linked to a higher risk of HTN in females, and short sleep was associated with an increased risk of coronary artery disease (CAD) in males. A statistically significant sex difference in the causal effect of OSA on CKD was also identified. In addition, using shrinkage estimates for IV selection, we detected several statistically significant causal effects of OSA on CVD-related outcomes, as well as distinct sex differences in the causal patterns of long sleep on CVD-related outcomes, with higher risks observed in females.

Methods

Overview of semi-empirical Bayesian method for calibrating genetic variant effect size estimates utilizing information across groups

While our method is general, we focus on the need to improve the estimation of variant effect sizes in the relatively small MVP female population and do so by borrowing information from the male population. Another simplification we make in the exposition is to focus on the "exposure" GWAS, even though the same framework can be applied to any trait GWAS, regardless of its role in an MR analysis. Throughout this paper, we use γ to represent "variant-exposure" effect size and Γ to represent "variant-outcome" effect size. To motivate our method, we consider a Bayesian prior on γ_{jF}, the female-specific effect size of the j-th SNP on the sleep phenotype, specifically

$$\gamma_{jF} \sim N\left(\theta_{jF}, \tau_{jF}^2\right), \qquad \text{(Equation 1)}$$

where θ_{jF} and τ_{jF} are the prior mean and standard deviation (SD), respectively. The approximate distribution of the "raw" female-specific effect size estimate (i.e., an estimate that relies on female data only) is given by

$$\widehat{\gamma}_{jF,raw} \sim N\left(\gamma_{jF}, \widehat{\sigma}^2_{\gamma,jF,raw}\right), \qquad \text{(Equation 2)}$$

where $\widehat{\sigma}_{\gamma,jF,raw}$ is the estimated standard error of $\widehat{\gamma}_{jF,raw}$. The normality assumption here is appropriate due to the large GWAS sample sizes,[30,31] regardless of the specific method used for estimation (maximum likelihood, method of moments, etc.).

Potential specification of the prior distribution of female-specific SNP effect sizes and resulting posterior estimates

An intuitive way to borrow information from the male for the female population is to specify the prior mean and variance θ_{jF}, τ_{jF}^2 in Equation 1 as the male-specific effect size estimate and its estimated variance. Formally, the prior is

$$\gamma_{jF} \sim N\left(\theta_{jF} = \widehat{\gamma}_{jM,raw}, \tau_{jF}^2 = \widehat{\sigma}^2_{\gamma,jM,raw}\right), \qquad \text{(Equation 3)}$$

leading to posterior

$$\gamma_{jF} \left| \widehat{\gamma}_{jF,raw}, \widehat{\sigma}^2_{\gamma,jF,raw}, \widehat{\gamma}_{jM,raw}, \widehat{\sigma}^2_{\gamma,jM,raw} \right. \sim N\left(R_{jF}, K_{jF}^2\right), \qquad \text{(Equation 4)}$$

where the posterior mean $R_{jF} = \frac{\widehat{\sigma}^2_{\gamma,jM,raw}}{\widehat{\sigma}^2_{\gamma,jF,raw} + \widehat{\sigma}^2_{\gamma,jM,raw}} \times \widehat{\gamma}_{jF,raw} + \frac{\widehat{\sigma}^2_{\gamma,jF,raw}}{\widehat{\sigma}^2_{\gamma,jF,raw} + \widehat{\sigma}^2_{\gamma,jM,raw}} \times \widehat{\gamma}_{jM,raw}$ and the posterior variance $K_{jF}^2 = \frac{1}{\frac{1}{\widehat{\sigma}^2_{\gamma,jF,raw}} + \frac{1}{\widehat{\sigma}^2_{\gamma,jM,raw}}}$. The posterior mean is a weighted average of the prior mean and sample mean, with the weight on the group-specific estimates being proportional to their precisions (i.e., the inverse of their variances). The posterior mean and variance are exactly identical to those from conventional fixed-effects (FE) inverse-variance meta-analysis of the sex-specific estimates ($\widehat{\gamma}_{j,meta}$, called the FE meta estimate henceforth). A more detailed illustration of the Bayesian approach to meta-analysis is given by Dominguez and Rice.[37] The FE meta estimate has been shown to be as efficient as pooling individual-level data when effects are identical across combined studies,[38] so in that setting, there is no penalty for using meta-analysis over any standard competing method.

However, the estimated strength of sex differences, i.e., $\widehat{\gamma}_{jF,raw} - \widehat{\gamma}_{jM,raw}$, is not considered in the FE meta estimate or standard competing methods, making their use unappealing when there are strong sex differences. To incorporate information on sex differences, we consider the prior with

$$\gamma_{jF} \sim N\left(\theta_{jF} = \widehat{\gamma}_{j,meta}, \tau_{jF}^2 = \left(\widehat{\gamma}_{jF,raw} - \widehat{\gamma}_{jM,raw}\right)^2 + \widehat{\sigma}^2_{\gamma,jM,raw}\right). \qquad \text{(Equation 5)}$$

Table 1. The proposed Bayesian framework for calibrating variant-trait effect size estimates

Likelihood	Prior distribution of γ_{jF}	Posterior mean of γ_{jF}	Estimator name
$\hat{\gamma}_{jF,raw} \sim N(\gamma_{jF}, \hat{\sigma}^2_{\gamma,jF,raw})$	$\gamma_{jF} \sim N(\hat{\gamma}_{jM,raw}, \hat{\sigma}^2_{\gamma,jM,raw})$	$\frac{\hat{\sigma}^2_{\gamma,jM,raw}}{\hat{\sigma}^2_{\gamma,jF,raw}+\hat{\sigma}^2_{\gamma,jM,raw}}\hat{\gamma}_{jF,raw}+\frac{\hat{\sigma}^2_{\gamma,jF,raw}}{\hat{\sigma}^2_{\gamma,jF,raw}+\hat{\sigma}^2_{\gamma,jM,raw}}\hat{\gamma}_{jM,raw}$	FE meta estimate, $\hat{\gamma}_{j,meta}$
	$\gamma_{jF} \sim N(\hat{\gamma}_{j,meta}, (\hat{\gamma}_{jF,raw}-\hat{\gamma}_{jM,raw})^2+\hat{\sigma}^2_{\gamma,jM,raw})$	$\frac{(\hat{\gamma}_{jF,raw}-\hat{\gamma}_{jM,raw})^2+\hat{\sigma}^2_{\gamma,jM,raw}}{(\hat{\gamma}_{jF,raw}-\hat{\gamma}_{jM,raw})^2+\hat{\sigma}^2_{\gamma,jM,raw}+\hat{\sigma}^2_{\gamma,jF,raw}}\hat{\gamma}_{jF,raw}+\frac{\hat{\sigma}^2_{\gamma,jF,raw}}{(\hat{\gamma}_{jF,raw}-\hat{\gamma}_{jM,raw})^2+\hat{\sigma}^2_{\gamma,jM,raw}+\hat{\sigma}^2_{\gamma,jF,raw}}\hat{\gamma}_{j,meta}$	APM estimate, $\hat{\gamma}_{jF,APM}$

The table summarizes the semi-empirical Bayesian model parameters behind the two proposed variant-trait effect size estimators. The raw effect size estimates are obtained from an analysis of a single stratum (here we focus on the female stratum). By specifying prior distributions on the variant-trait effect sizes (second column), the raw estimates are shrunk toward the prior means to become the estimates provided in the posterior mean column. The last column, estimator name, provides the name and notation of the resulting estimators (FE meta estimate and adaptive posterior mean [APM] estimate).

Here, the prior mean is the efficient FE meta estimate $\hat{\gamma}_{j,meta}$, while the strength of sex differences is incorporated in the prior variance. The posterior distribution of γ_{jF} is normal, with mean $R_{jF} = \frac{\hat{\delta}^2}{\hat{\delta}^2+\hat{\psi}^2}\hat{\gamma}_{jF,raw} + \frac{\hat{\psi}^2}{\hat{\delta}^2+\hat{\psi}^2}\hat{\gamma}_{j,meta}$, where $\hat{\delta}^2 = (\hat{\gamma}_{jF,raw} - \hat{\gamma}_{jM,raw})^2 + \hat{\sigma}^2_{\gamma,jM,raw}$ is related to both the difference in the effect size estimate *and* the variance of the male-specific estimate. Here, $\hat{\psi}^2$ is $\hat{\sigma}^2_{\gamma,jF,raw}$. The posterior variance can be written as $K^2_{jF} = \frac{1}{\frac{1}{\hat{\sigma}^2_{\gamma,jF,raw}}+\frac{1}{(\hat{\gamma}_{jF,raw}-\hat{\gamma}_{jM,raw})^2+\hat{\sigma}^2_{\gamma,jM,raw}}}$. Because the posterior mean adapts $\hat{\gamma}_{jF,raw}$ to the observed differences in sex-specific estimates, we call it the adaptive posterior mean (APM) estimator ($\hat{\gamma}_{jF,APM}$). From Equation 5, the prior mean and variance depend not only on sex-specific groups but also on sex-combined effect estimates and sex differences. This is why we refer to our framework as a "semi-empirical" Bayes approach.

The APM estimator is related to the AW estimator, initially proposed for gene-environment interactions or gene-secondary outcome associations in case-control studies.[34–36] In both of those contexts, the AW estimator was developed to provide a population-level estimate (i.e., not specific to either cases or controls) by adaptively combining information from both groups using weighting. In our approach, we instead use shrinkage estimators to obtain group-specific estimates. The APM estimator also differs from the original AW in its weighting parameters. APM incorporates the variance of group-specific effect estimates into its prior variance for γ_{jF}, to avoid underestimating posterior variance due to smaller estimated sex differences (i.e., $\hat{\gamma}_{jF,raw} - \hat{\gamma}_{jM,raw} \approx 0$). In other words, if $\hat{\sigma}^2_{\gamma,jM,raw} = 0$ in Equation 5, then the APM estimator may reduce to the AW estimator. Table 1 summarizes the derivation of the FE meta and APM estimates under the Bayesian normal-normal modeling scheme. As stated earlier, we focus on the calibration of the female effects $\hat{\gamma}_F$, but the framework is general and can be similarly applied to the male population.

Exposure-outcome causal effect estimation

Two-sample MR approaches estimate causal effects from two independent sets of summary statistics. These describe variant associations with an exposure and with the outcome phenotype, where the variants are selected to be valid IVs for the exposure of interest.[30,39–42] A causal effect β_j of exposure on outcome can then be estimated using estimated associations of variant j with exposure ($\hat{\gamma}_{j,raw}$) and outcome ($\hat{\Gamma}_{j,raw}$) via the Wald ratio estimate $\hat{\beta}_{j,raw} = \frac{\hat{\Gamma}_{j,raw}}{\hat{\gamma}_{j,raw}}$. Using multiple IVs, the causal effect β can then be estimated by aggregating estimates from all valid IVs through various weighting approaches. Here, we assume that all variants are valid IVs. Our proposed Bayesian framework, therefore, is conceptualized as a preliminary step before applying MR methods. More specifically, we first calibrate the $\hat{\gamma}_{raw}$ estimates using the proposed Bayesian models. Then, the shrinkage estimates, i.e., the posterior means and SDs in Equation 4, are used as inputs in existing MR algorithms. The causal effect is then estimated using the newly estimated $\hat{\gamma}_{meta}$ and $\hat{\gamma}_{APM}$ (with corresponding posterior SD) coupled with the (raw) variant-outcome estimated effect sizes. A schematic overview of the sex-specific MR analysis and the proposed Bayesian framework is illustrated in Figure 1. We also consider multivariable MR (MVMR), which may be used to account for potential confounders (see Appendix A).

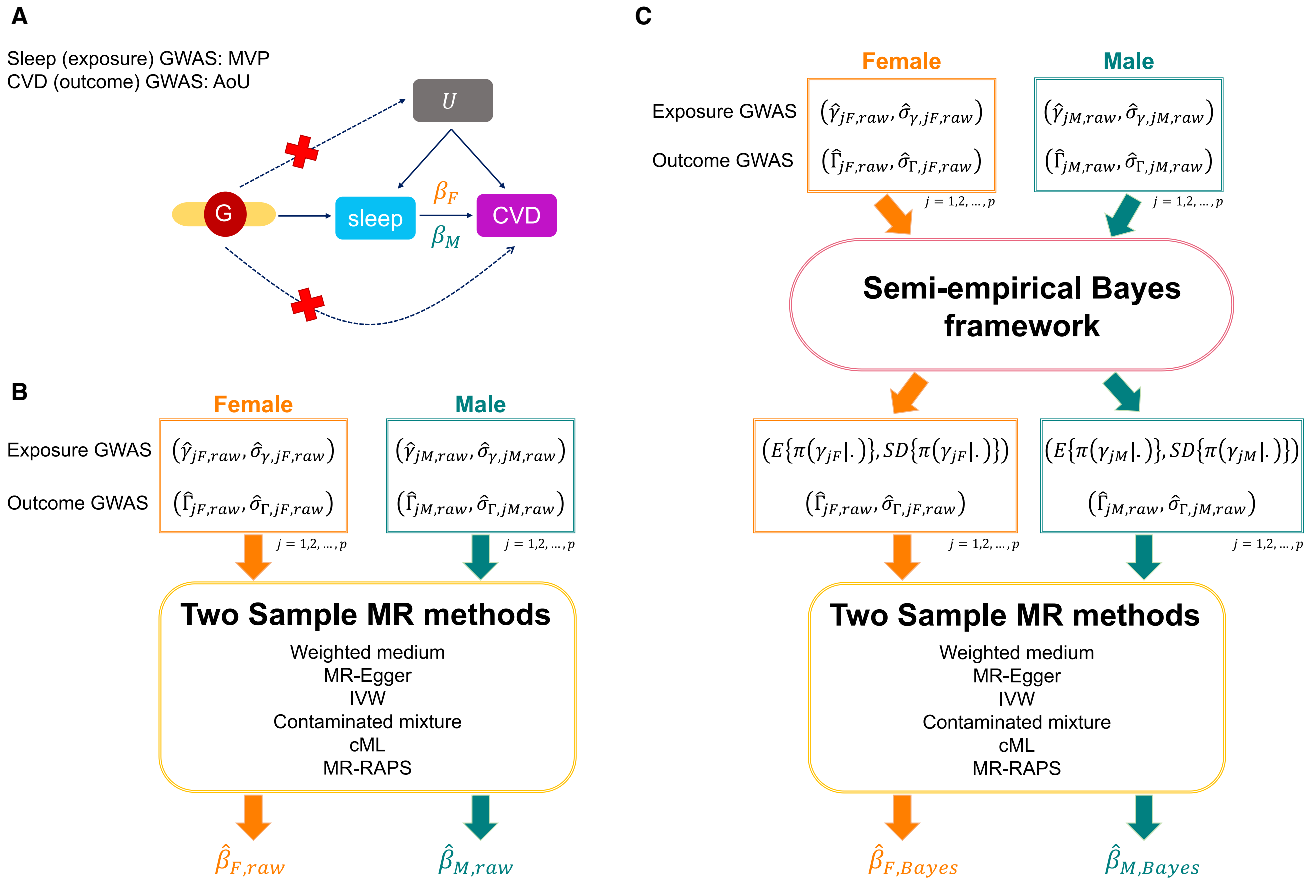

Figure 1. Schematic overview of the sex-specific MR analysis

(A) A causal diagram underlying the MR framework in this manuscript. We consider sleep-related phenotypes to be the exposure factors and cardiovascular-related measures as outcome variables. β_F denotes the underlying female-specific exposure-outcome causal effect; β_M represents the underlying male-specific exposure-outcome causal effect. In our analysis, β_F and β_M are estimated separately. The GWAS summary statistics for sleep phenotypes were derived from the MVP dataset, and the summary statistics for cardiovascular-related diseases were computed in AoU.

(B) Estimation of sex-specific causal effects using the two-sample MR approaches. The inputs for the two-sample MR methods are raw sex-specific exposure (female: $\hat{\gamma}_{jF,raw}$ and $\hat{\sigma}_{\gamma,jF,raw}$; male: $\hat{\gamma}_{jM,raw}$ and $\hat{\sigma}_{\gamma,jM,raw}$) and outcome GWAS summary statistics (female: $\hat{\Gamma}_{jF,raw}$ and $\hat{\sigma}_{\Gamma,jF,raw}$; male: $\hat{\Gamma}_{jM,raw}$ and $\hat{\sigma}_{\Gamma,jM,raw}$) from independent samples. The outputs are estimated female-specific causal effect $\hat{\beta}_{F,raw}$ and estimated male-specific causal effect $\hat{\beta}_{M,raw}$.

(C) The proposed Bayesian method. We first calibrate the raw sex-specific exposure effects by borrowing information from one sex group to use in the other or across both sex groups. Two-sample MR analysis then uses the shrinkage exposure summary statistics, i.e., the posterior mean and posterior SD of γ (female: $E\{\pi(\gamma_{jF}|.)\}$ and $SD\{\pi(\gamma_{jF}|.)\}$; male: $E\{\pi(\gamma_{jM}|.)\}$ and $SD\{\pi(\gamma_{jM}|.)\}$), with the raw outcome summary statistics to provide a more robust basis for causal effect estimation. We use $\pi(\gamma|.)$ to denote the posterior distribution of γ. The outputs are the estimated female-specific causal effect $\hat{\beta}_{F,Bayes}$ and estimated male-specific causal effect $\hat{\beta}_{M,Bayes}$; both use shrinkage exposure effect estimates in their construction.

MR, Mendelian randomization; F, female; M, male; CVD, cardiovascular disease; GWAS, genome-wide association study; MVP, Million Veteran Program; AoU, All of Us; SD, standard deviation; cML, constrained maximum likelihood.

Results

Simulation studies

We used simulations primarily to evaluate and compare the performance of exposure-outcome causal effect (β) estimation using raw ($\hat{\gamma}_{raw}$) and shrinkage ($\hat{\gamma}_{meta}$ and $\hat{\gamma}_{APM}$) variant-exposure effects with respective SD estimates, combined with each selected MR method, particularly focusing on the population with a smaller sample size (female population in our analysis). For our Bayesian methods, the posterior SDs of γ are treated as standard error estimates. We also incorporated the AW estimator ($\hat{\gamma}_{AW}$) as another approach for calibrating female-specific variant-exposure effect size estimates. The two-sample MR methods considered in the analyses are summarized in Table 2 (Appendix A).

To mimic the structure of the MVP dataset, we generated 2,000 female individuals and 20,000 male individuals for the exposure GWAS, maintaining a similar proportion of females to males as in the MVP. For the outcome GWAS, we generated balanced datasets of 10,000 individuals for both female and male populations. We generated 100 independent SNPs as IVs in all simulations, with all allele frequencies set at 0.3. We

Table 2. Two-sample MR methods used in both simulation studies and real-data analyses

MR method	Description	Software used	Reference
MR-RAPS (primary analysis)	estimation: adjusted profile likelihood estimation with down-weighting of outliers via a robust loss function<br>assumptions: InSIDE, pleiotropic effects follow normal distribution with mean zero	R package: mr.raps<br>R function: mr.raps.overdispersed.robust()	Zhao et al.[30]
IVW	estimation: aggregate multiple Wald ratio estimates using fixed effect inverse-variance weighting<br>assumptions: InSIDE, zero average pleiotropy effect	R package: MendelianRandomization<br>R function: mr_allmethods()	Burgess et al.[27]
Constrained ML	estimation: maximum likelihood estimation with a constraint on the number of invalid IVs<br>assumptions: plurality valid	R package: MendelianRandomization<br>R function: mr_cML()	Xue et al.[42]
Contamination mixture	estimation: profile likelihood estimation assuming that the ratio estimates follow two normal distributions for valid and invalid IVs, respectively<br>assumption: plurality valid	R package: MendelianRandomization<br>R function: mr_conmix()	Burgess et al.[41]
Weighted median	estimation: compute the median of the causal effect estimated from multiple IVs, weighted by the inverse of the estimate's sampling variance<br>assumption: majority valid	R package: MendelianRandomization<br>mr_allmethods()	Bowden et al.[43]
MR Egger	estimation: weighted meta-regression with an intercept term to capture the average horizontal pleiotropy effect<br>assumption: InSIDE	R package: MendelianRandomization<br>R function: mr_allmethods()	Bowden et al.[39]
Robust IVW and MR-Egger	estimation: apply robust regression to down-weight or exclude variants with heterogeneous causal estimates	R package: MendelianRandomization<br>R function: mr_allmethods()	Rees et al.[40]

Summary of the two-sample MR methods used in the simulation studies and real-data analyses. The names of the methods are listed in the first column. A brief description of the estimation approach and the underlying assumptions for each method are provided in the second column. The corresponding software for applying these methods is listed in the third column, and the references are provided in the last column. InSIDE, instrument strength independent of direct effect.

use $D_{j\gamma} = \gamma_{jF} - \gamma_{jM}, j = 1, 2, \ldots, p$ to denote the strength of sex differences in variant associations with the exposure, γ. We write $D_\gamma = \{j | D_{j\gamma} = \gamma_{jF} - \gamma_{jM} \neq 0\}$ and $|D_\gamma|$ to represent the set of variants and the number of variants with sex differences in variant-exposure effect size, respectively. We considered three simulation scenarios: (1) fixed $D_{j\gamma} = 0.05$ if variant $j \in D_\gamma$, (2) random $D_{j\gamma}$, and (3) using MVP OSA GWAS summary statistics to guide the simulated differences $D_{j\gamma}$, which consists of strong $D_{j\gamma}$ patterns with weak IVs in the female population (average F-statistic < 10). In simulations 2 and 3, all variants $j \in D_\gamma$ (i.e., $|D_\gamma| = 100$). Within each simulation, we also considered a few levels of sex differences in causal effect settings (i.e., $\beta_F \neq \beta_M$). A more detailed description of the simulation studies is provided in Appendix A, Note S1, and Tables S1 and S2. Table S3 summarizes the average F-statistic (across SNPs and across simulation repetitions), which measures the average strength of IVs, when using $\hat{\gamma}_{raw}$ and $\hat{\gamma}_{APM}$. Across simulation settings, the average F-statistics derived from $\hat{\gamma}_{APM}$ in the female stratum are more than 3-fold higher than those from $\hat{\gamma}_{raw}$, demonstrating improved IV strength.

We summarized the estimation performance of the $\hat{\beta}_F$ and $\hat{\beta}_M$ in the simulation studies using two metrics: (1) the mean-squared error (MSE) of the estimated effect and (2) the 95% confidence interval (CI)'s actual coverage of the true effect, where these 95% CIs for β under each MR method were computed using standard asymptotic normality properties. The female results from simulations with no sex differences in the causal effect are presented in Figure 2 and summarized below. The full simulation results, including the male-specific causal effect estimation and sex differences in causal effect simulations, are summarized in Note S1 and Figures S1–S3.

Results from simulation setting 1: Fixed sex differences in variant-exposure effect sizes

These results are provided in Figures 2A and 2B. The $\hat{\gamma}_{meta}$ estimator performs best when D_γ includes only 10% of the variants (i.e., $|D_\gamma| = 10$), compared to other shrinkage methods and to the raw $\hat{\gamma}_{F,raw}$. The estimators $\hat{\gamma}_{F,APM}$ and $\hat{\gamma}_{F,AW}$ had similar performance, and both gave better causal effect estimates than $\hat{\gamma}_{F,raw}$, regardless of the proportion of variants with sex differences. We found that $\hat{\gamma}_{F,APM}$ performed better than $\hat{\gamma}_{meta}$ when more than 10% of variants were being selected into D_γ. The coverage rate of β_F using $\hat{\gamma}_{F,APM}$ was lower than that of $\hat{\gamma}_{F,raw}$ when many variants were included in D_γ. However, $\hat{\gamma}_{F,APM}$ always achieved a smaller MSE for β_F compared with $\hat{\gamma}_{F,raw}$.

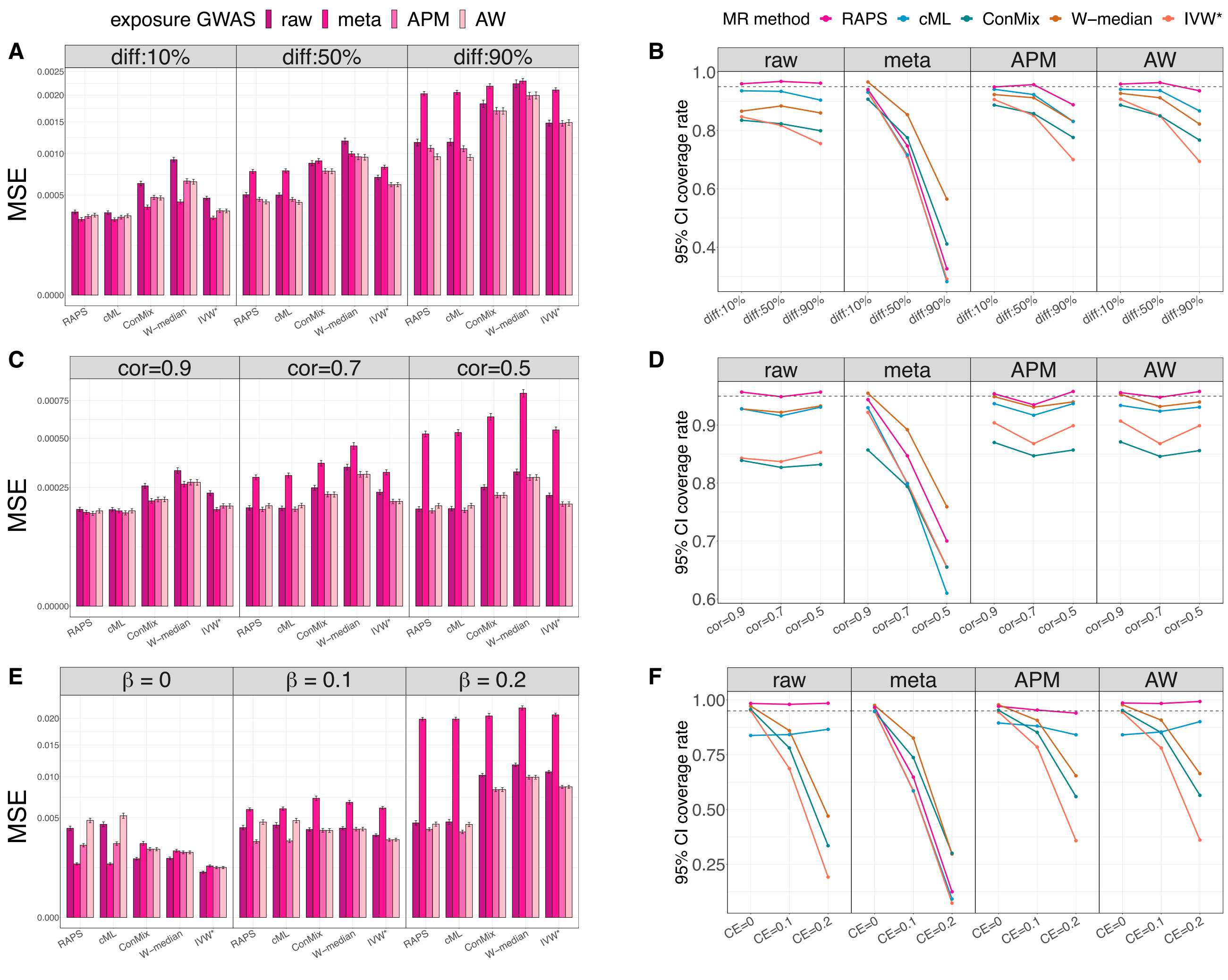

Figure 2. MSE and 95% confidence interval coverage rate for female-specific causal effect estimation

Results from estimating female-specific causal effects β_F under three simulation scenarios where there are no sex differences in causal effects (i.e., $\beta_F = \beta_M$) across sex groups.

The left image shows the MSEs of the estimated female-specific causal effects, while the right image presents the 95% confidence interval coverage rates of the true underlying causal effects. We considered five two-sample MR methods for estimating the causal effect, which are MR-RAPS (RAPS), constrained maximum likelihood (cML), contaminated mixture (ConMix), weighted median (W-median), and penalized and robust IVW (IVW*). In the MSE results, the uncalibrated approach ($\hat{\gamma}_{raw}$) is represented by the bars with darker colors. The other three shrinkage approaches ($\hat{\gamma}_{meta}$, $\hat{\gamma}_{APM}$, and $\hat{\gamma}_{AW}$) are shown in gradient colors. For the coverage rate results, the uncalibrated approach is shown in the first column, and the other three shrinkage approaches are shown in the second to fourth columns. The results of fixed sex differences in variant-exposure effect simulations are presented in (A) and (B). The results of random sex differences in variant-exposure effect simulations are shown in (C) and (D). The results of using MVP OSA GWAS summary statistics to guide sex-specific variant-exposure effect simulations are shown in (E) and (F). The underlying true causal effect is set at $\beta_F = 0.1$ in (A)–(D). The underlying true causal effects are shown on the top of (E) and the bottom of (F). MSEs were computed over 1,000 simulation replicates. Intervals around the estimated MSE correspond to the MSE ± one estimated standard error. MSE, mean square error; MR, Mendelian randomization; APM, adaptive posterior mean; AW, adaptive weight; diff, different level of sex differences in variant-exposure effects; Cor, correlation between female and male variant-exposure effect; CE, causal effect; RAPS, MR-RAPS; cML, constrained maximum likelihood; ConMix, contaminated mixture; W-median, weighted medium; IVW*, penalized and robust IVW.

Results from simulation setting 2: Random sex differences in variant-exposure effect sizes

For random sex differences in γ (Figures 2C and 2D), when every variant has a sex difference, i.e., all $j \in D_\gamma$, and most sex difference $D_{j\gamma}$ were strong, the estimate $\hat{\beta}_F$ that relies on the $\hat{\gamma}_{meta}$ had a higher MSE than the one relying on raw $\hat{\gamma}_{F,raw}$. In contrast, using $\hat{\gamma}_{F,APM}$ improved β_F estimation performance in terms of MSE and performed similarly to $\hat{\gamma}_{F,AW}$. In these simulations, the APM estimator $\hat{\gamma}_{F,APM}$ resulted in similar 95% CI coverage compared to the raw approach but achieved a smaller MSE for β_F.

Results from simulation setting 3: Using OSA GWAS summary statistics to guide the simulated variant-exposure effect sizes

Here, all variants have substantial sex differences in γ, while the selected variants are all weak IVs, meaning that the F-statistic is <10 in the female population. Using $\hat{\gamma}_{F,APM}$ and $\hat{\gamma}_{F,AW}$ improved β_F estimation as demonstrated

by the improved MSE (Figures 2E and 2F). In most cases, $\hat{\gamma}_{F,APM}$ performed better than $\hat{\gamma}_{F,AW}$. Both $\hat{\gamma}_{F,APM}$ and $\hat{\gamma}_{F,AW}$ approaches improved the 95% CI coverage rate compared to using $\hat{\gamma}_{F,raw}$. These results highlight that even though less information could be transferred from the male to the female population in these simulations (due to the strong sex differences in γ), adaptive estimates still improved causal effect estimation. Moreover, the estimates $\hat{\gamma}_{meta}$ had a larger MSE than the raw approach under most settings and performed poorly when the female causal effect β_F was non-null.

In summary, borrowing power from the stratum with the larger sample size to give to the stratum with the lower sample size using adaptive (i.e., shrinkage) variant-exposure estimates ($\hat{\gamma}_{F,APM}$ and $\hat{\gamma}_{F,AW}$) improves β_F estimates. Using estimators $\hat{\gamma}_{F,APM}$ and $\hat{\gamma}_{F,AW}$ performed well in most simulation studies, regardless of the degree of sex differences $D_{\gamma j}$. The estimator $\hat{\gamma}_{meta}$ performs best when the underlying true γ were similar in the two groups. Among the two-sample MR methods considered, MR using robust adjusted profile score (RAPS), known to perform well when weak instruments are used, demonstrated at least no worse performance than the other methods.

Secondary simulation studies

Note S2 and Figures S4–S16 provide results from secondary simulation studies. We expanded upon simulation setting 1 and further examined two scenarios: (1) estimation in the presence of pleiotropic effects (both balanced and directional) of some IVs and (2) calibration of both γ and Γ effect estimates using the proposed framework. We also considered an increased sample size of the female population in the exposure GWAS to simulate a scenario where borrowing information from the male group may be less useful. Lastly, we evaluated the performance of a test for sex differences, i.e., a test of the null hypothesis $H_0 : \beta_F = \beta_M$, using the estimated β_F and β_M based on various MR approaches (but always using the raw variant-exposure $\hat{\gamma}_{raw}$ and variant-outcome associations $\hat{\Gamma}_{raw}$).

In brief, $\hat{\gamma}_{APM}$ and $\hat{\gamma}_{AW}$ substantially improved the estimation accuracy of γ (Figures S4–S6) compared to the $\hat{\gamma}_{raw}$ in all settings and, under substantial sex differences, performed better than $\hat{\gamma}_{meta}$. In the simulations where pleiotropy was present, $\hat{\gamma}_{APM}$ still produced a lower MSE when estimating β_F compared to $\hat{\gamma}_{raw}$ (Figures S7 and S9). The penalized and robust IVW, contaminated mixture, constrained maximum likelihood (cML), and MR-RAPS performed similarly in the balanced pleiotropy analysis. In the directional pleiotropy analysis (Figures S9 and S10), where the assumptions of the MR-RAPS method are violated, MR-RAPS showed reduced power, with the 95% coverage rate most affected due to underestimation of standard error. In terms of MSE, MR-RAPS performed less well compared to the robust IVW, performed similarly to cML, and still outperformed traditional methods such as MR-Egger and IVW. Additional calibration of Γ improved the estimation of the β_F when males and females had the same causal effect but not otherwise (Figures S11 and S12). When increasing female sample sizes, the shrinkage approaches resulted in nearly the same MSE for β_F estimation as that for $\hat{\gamma}_{raw}$, indicating that no estimation efficiency is lost when using the shrinkage approaches, even though potentially less information is transferred from the male population (Figures S13 and S14). The test of sex differences in the causal effect between groups showed that MR-RAPS controls type I error rate close to the nominal level under most settings while maintaining similar power compared to other MR methods for detecting sex differences in causal effects (Figures S15 and S16).

Sex-specific causal estimates of the effect of sleep phenotypes on cardiovascular-related outcomes

We estimated sex-specific causal effects of sleep phenotypes on CVD-related outcomes. Specifically, we considered five binary sleep traits: OSA, insomnia, short sleep duration, long sleep duration, and excessive daytime sleepiness (sleepiness). The outcomes were six binary CVD-related phenotypes, with data from AoU: atrial fibrillation (AF), CAD, CKD, heart failure (HF), HTN, and type 2 diabetes mellitus (T2DM). Detailed information on the AoU analysis is provided in Note S4. A summary of the results from MVP GWASs, including sample sizes for sex-specific sleep trait GWASs, Miami plots, and Q-Q plots, is presented in Note S5. The MVP sex-specific GWASs of sleep phenotypes were conducted using the same procedure described elsewhere.[16] We only included individuals from the White harmonized race/ethnicity and genetic ancestry (HARE) group, as the large HARE group. Based on these results, we selected variants and performed variant-outcome associations in the AoU dataset, focusing on the group of White individuals. For each trait, two types of analyses were performed: one adjusting for body mass index (BMI) and the other without BMI adjustment, in both the exposure GWAS and outcome association analyses. BMI was adjusted for because it is recognized as a strong risk factor for sleep phenotypes. For example, high BMI is the strongest risk factor for OSA. This adjustment aims to estimate sleep-CVD causal effects in pathways that are independent of BMI. We only used common variants (minor-allele frequency $\geq$ 0.01) with imputation quality scores $\geq$ 0.8. More details are summarized in Appendix A.

IV selection strategies

We applied p value thresholding and clumping procedures on exposure GWASs using the "clump_data" function from the "TwoSampleMR" R package (v.0.6.8; https://mrcieu.github.io/TwoSampleMR/reference/clump_data.html). The clumping window was set to 10,000 kb, the correlation threshold was set to 0.001, and the European population reference panel was used. Due to having a limited number of variants (or even no variants) with $p < 5 \times 10^{-8}$ and $p < 10^{-7}$ in the female sleep GWASs, a p value threshold

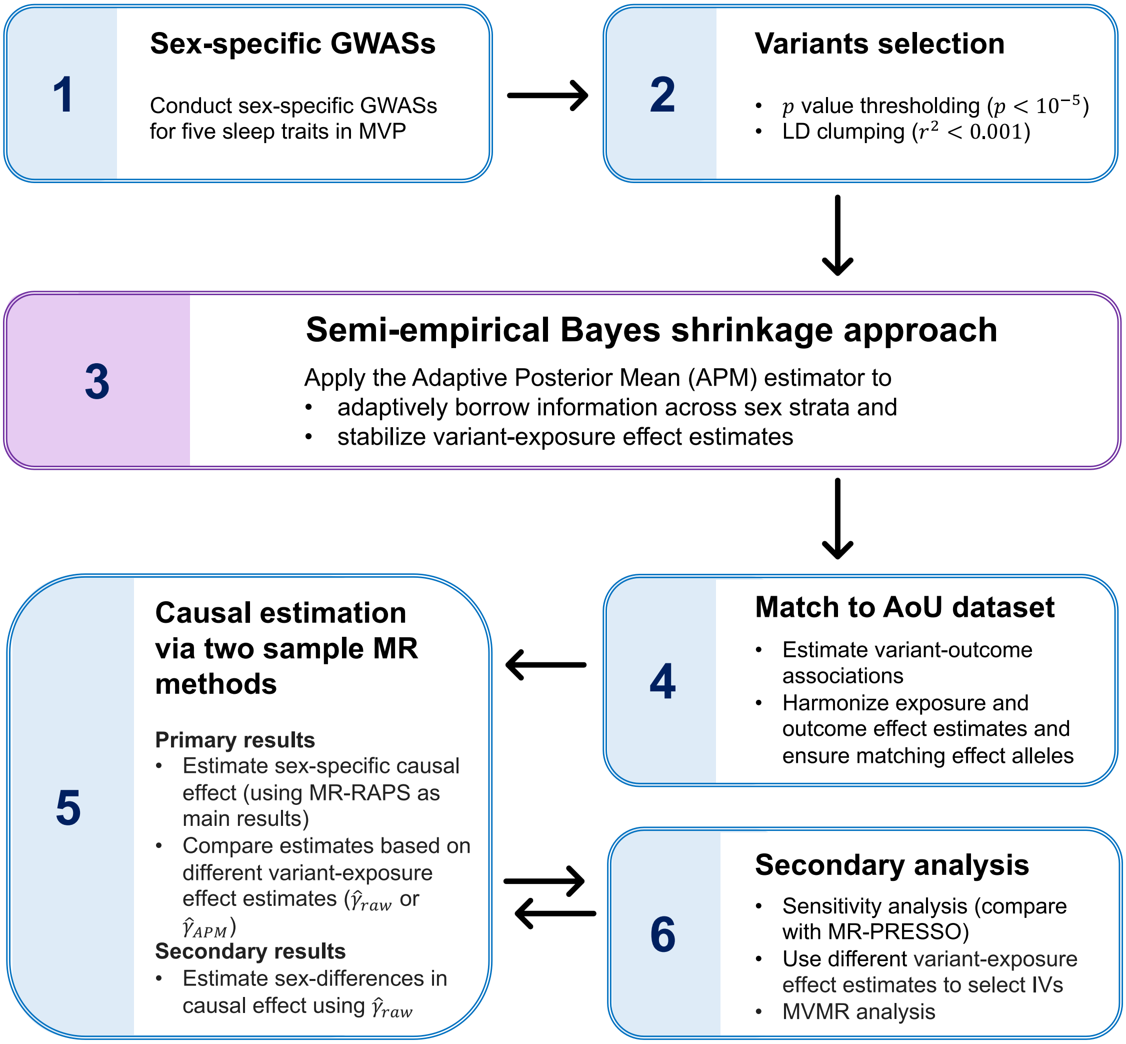

Figure 3. Overview of data analysis workflow
The main workflow consists of six key steps. In step 1, we performed sex-specific GWASs of five sleep traits using participants from MVP. Step 2 involved the selection of IVs based on the sleep GWASs. In step 3, we applied our proposed semi-empirical Bayesian approach to stabilize the variant-exposure effect estimates, particularly for the female group, which had a small sample size in MVP. Step 4 involved harmonizing the exposure and outcome datasets by aligning allele effects, where the variant-outcome associations were computed from the AoU dataset. In step 5, we conducted the primary two-sample MR analysis using the MR-RAPS method to estimate sex-specific causal effects, followed by the testing of sex differences in causal effect estimates. Finally, in step 6, we conducted secondary analyses, including sensitivity analyses comparing results from MR-RAPS and MR-PRESSO, evaluating alternative IV selection strategies based on different variant-exposure effect estimates and MVMR analysis. GWAS, genome-wide association study; MVP, Million Veteran Program; IV, instrumental variable; AoU, All of Us; MR, Mendelian randomization; MVMR, multivariable MR.

of 10^{-5} was selected for IV selection in all analyses. The number of variants remaining after p value thresholding ($p < 10^{-5}$) and clumping is given in Table S4. We then matched the list of variants selected as targeted IVs from the exposure GWAS to those available in the AoU dataset. Harmonization of the exposure and outcome datasets was performed using the "harmonise_data" function from the TwoSampleMR R package (v.0.6.8; https://mrcieu.github.io/TwoSampleMR/reference/harmonise_data.html). This function ensures that the effect estimates for each variant are aligned to the same effect allele across exposure and outcome datasets by checking both the direction of effect sizes and the allele frequency of the reported "effect allele." It also accounts for strand ambiguity and removes variants with mismatched allele frequencies or palindromic alleles that cannot be reliably aligned. Detailed instructions for the harmonization procedure can be found at https://mrcieu.github.io/TwoSampleMR/articles/harmonise.html. We employed two strategies for IV selection in the primary analyses: (1) sex-specific IVs were selected based on sex-specific GWAS results ($\hat{\gamma}_{raw}$), potentially resulting in different variants for male and female analyses, and (2) IVs were selected using APM shrinkage estimates ($\hat{\gamma}_{APM}$), where additional "potential" IVs that could not be selected using $\hat{\gamma}_{F,raw}$ estimates (due to the smaller female sample size) were included by borrowing information from the male population. In the secondary analysis, we applied the $\hat{\gamma}_{meta}$ estimate for IV selection in sex-combined analyses. Figure 3

presents a flowchart summarizing the data analysis workflow.

Sex-specific causal estimates

The primary results use the $\widehat{\gamma}_{APM}$ estimates with the MR-RAPS method, specifically developed for handling weak instruments and demonstrating superior performance in simulation studies. Figure 4 shows sex-specific causal effect estimates of sleep-related phenotypes on CVD-related outcomes, employing two different IV selection strategies: IVs selected based on sex-specific $\widehat{\gamma}_{APM}$ (primary, proposed) and sex-specific $\widehat{\gamma}_{raw}$. Three pairs of causal effect estimates showed statistically significant associations ($p < 0.05$) when IVs were selected using $\widehat{\gamma}_{raw}$: insomnia on CKD in females (odds ratio [OR]: 1.23, 95% CI: 1.01–1.49), long sleep on HTN in females (OR: 1.03, 95% CI: 1.00–1.06), and short sleep on CAD in males (OR: 1.32, 95% CI: 1.03–1.69). In contrast, when $\widehat{\gamma}_{APM}$ was used for IV selection, enabling more potential IVs to be selected by borrowing information across sex groups, additional statistically significant causal estimates were identified. For instance, in females, we found a significant effect of OSA on T2DM (OR: 1.32, 95% CI: 1.05–1.66) in BMI-unadjusted analyses and also an effect on HTN (OR: 1.14, 95% CI: 1.03–1.25) in BMI-adjusted analyses. Notably, the causal relationships between insomnia and CKD (OR: 1.38, 95% CI: 1.07–1.79) and long sleep and HTN (OR: 1.04, 95% CI: 1.00–1.09) were replicated in the APM selection analyses (both from BMI-adjusted analyses). Among males, multiple causal associations were observed between OSA and CVD-related outcomes, including OSA on CKD (BMI unadjusted, OR: 1.23 and 95% CI: 1.03–1.47; BMI adjusted, OR: 1.27 and 95% CI: 1.02–1.59) and OSA on HF in the BMI-unadjusted analysis (OR: 1.30, 95% CI: 1.04–1.63).

Sex difference tests identified a statistically significant difference in the causal effect of OSA on CKD, with a stronger effect in males (Table 3; Figure S17). Also, there were sex differences in the causal effects of long sleep on several CVD-related outcomes: long sleep increased the risk of CAD, CKD, HTN, and T2DM in females but was protective in males. These sex differences were statistically significant in the APM IV selection analysis (Table 3; Figures S19 and S20).

The key findings from sex-specific causal effect estimation and sex difference tests are summarized in Tables 3 and 4. Full results, including the causal estimation using $\widehat{\gamma}_{raw}$, $\widehat{\gamma}_{meta}$, and $\widehat{\gamma}_{AW}$, as well as the sex differences in causal estimates tests, are summarized in Figures S17–S20. Causal estimates from other considered MR methods are summarized in Data S1 ($\widehat{\gamma}_{raw}$ IV selection) and S2 ($\widehat{\gamma}_{APM}$ IV selection). The average F-statistics are summarized in Table S5. Focusing on the female population, when selecting IVs using sex-specific $\widehat{\gamma}_{raw}$, the average F-statistics are similar between $\widehat{\gamma}_{raw}$ and $\widehat{\gamma}_{APM}$, consistent with the fairly similar causal effect estimates between analyses that rely on $\widehat{\gamma}_{raw}$ and $\widehat{\gamma}_{APM}$ when selecting IVs based on $\widehat{\gamma}_{raw}$ (Figures S17 and S18). In contrast, when IVs are selected based on $\widehat{\gamma}_{APM}$, the average F-statistic is substantially higher for $\widehat{\gamma}_{APM}$ compared to $\widehat{\gamma}_{raw}$ because selecting IVs based on $\widehat{\gamma}_{APM}$ identified SNPs that had relatively weak $\widehat{\gamma}_{raw}$ associations in the female stratum before borrowing information from the male stratum. Indeed, several causal associations are statistically significant in female-specific analysis only when using $\widehat{\gamma}_{APM}$ IV selection.

Results from secondary analyses

We compared the causal effect estimates of MR-RAPS, which we used in the primary analyses, with those of MR-PRESSO,[44] a widely used approach for detecting IVs with pleiotropic effects and removing them in MR analysis. The results from the two methods are similar and are summarized in Note S3 and Figures S21 and S22.

A comparison of causal effect estimates using $\widehat{\gamma}_{raw}$ and $\widehat{\gamma}_{APM}$ for IV selection is presented in Figure S23. In the male population, more consistent results were observed across different IV selection methods. For the analysis of the OSA phenotype, the $\widehat{\gamma}_{APM}$ selection strategy identified more variants as IVs in both male and female populations. Several significant causal effects between OSA and CVD-related outcomes were only detected when $\widehat{\gamma}_{APM}$ was used for IV selection. These findings highlight an additional advantage of our proposed shrinkage estimate: it not only helps correct for weak IV bias in causal effect estimation but also enhances the IV selection process, increasing the potential for identifying novel causal effects.

We applied $\widehat{\gamma}_{meta}$ for IV selection in the sex-combined analysis. In this analysis, the variant-exposure effect estimates were based on $\widehat{\gamma}_{meta}$, and the variant-outcome effect estimates were computed from a sex-combined analysis in the AoU study. The results are shown in Figure S24. Significant causal relationships between OSA and several CVD-related outcomes were identified, consistent with the findings from the primary analysis using $\widehat{\gamma}_{APM}$ for IV selection. However, some of the significant sex-specific findings, such as the female-specific causal effect of long sleep on HTN, were not identified in sex-combined analysis, likely because the $\widehat{\gamma}_{meta}$ is closed to $\widehat{\gamma}_{M,raw}$ due to the predominantly male sample size in MVP.

Lastly, as another approach for estimation of causal effects while accounting for BMI as a common cause of sleep exposures and CVD outcomes, we applied MVMR to estimate BMI-adjusted causal effects while using summary statistics derived from the BMI-unadjusted GWAS. Specifically, BMI was incorporated as an additional exposure in the MVMR model. We applied the robust multivariable inverse-variance weighted (MV-IVW) method in the MVMR analysis. Results from the MVMR analysis are presented in Figure S25, Table S6, and Data S3, with further details provided in Note S3 and Appendix A. We compare the MVMR results to the two sets of results: (1) univariate MR using BMI-unadjusted summary statistics (as this analysis relies on exactly the same set of IVs as the univariate analysis)

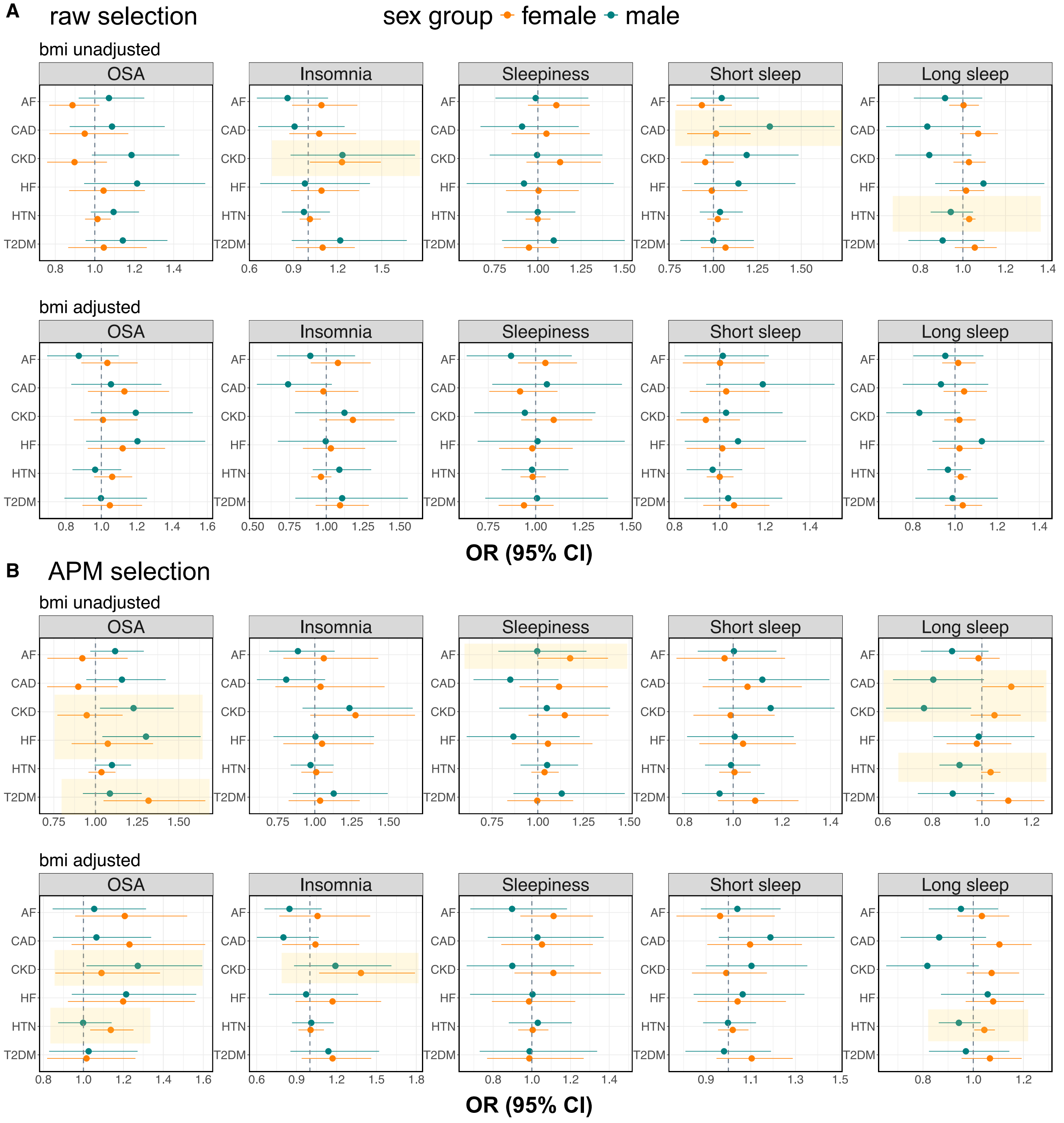

Figure 4. Results from sex-specific causal effect estimation
(A) Provides sex-specific causal effect estimates with the corresponding 95% CIs based on IVs selected by using $\hat{\gamma}_{raw}$, while (B) shows results using $\hat{\gamma}_{APM}$ for IV selection. The estimated causal effects (from MR-RAPS) with $\hat{\gamma}_{APM}$ variant-exposure effect estimates are displayed on an OR scale. In each image, variant-phenotype estimates without BMI adjustment are displayed at the top, and those with BMI adjustment are shown on the bottom. Female-specific results are indicated in orange, while male-specific results are shown in green. Vertical dashed lines indicate the null causal effects. The exposure variables are shown as the titles of the boxes, while the row names provide the outcome variables. Statistically significant results ($p < 0.05$) for either the female- or male-specific analysis are highlighted with yellow background. CI, confidence interval; APM, adaptive posterior mean; IV, instrumental variable; MR-RAPS, MR using robust adjusted profile score method; MR, Mendelian randomization; OR, odds ratio; BMI, body mass index; OSA, obstructive sleep apnea; AF, atrial fibrillation; CAD, coronary artery disease; CKD, chronic kidney disease; HF, heart failure; HTN, hypertension; T2DM, type 2 diabetes mellitus.

and (2) using BMI-adjusted summary statistics (as this analysis has the same purpose of estimating causal effects that are independent of BMI).

First, results from comparing MVMR to univariate MR using BMI-unadjusted summary statistics are shown in Figures S26 and S27. Focusing on the primary univariate

Table 3. Top findings from sex-differences test in causal effect estimation

Exposure	Outcome	$\hat{\beta}_{F,APM}$	$\hat{\beta}_{M,APM}$	$\hat{\beta}_{F,raw} - \hat{\beta}_{M,raw}$	Sex difference p value	Sex difference FDR p value
$\hat{\gamma}_{raw}$ selection BMI-unadj						
OSA	CKD	−0.108 (−0.277, 0.060)	0.171 (−0.015, 0.357)	−0.275 (−0.521, −0.029)	0.028	0.500
$\hat{\gamma}_{APM}$ selection BMI-unadj						
Long sleep	CAD	0.112 (0.001, 0.223)	−0.219 (−0.445, 0.008)	0.328 (0.078, 0.578)	0.009	0.118
Long sleep	CKD	0.049 (−0.046, 0.146)	−0.267 (−0.489, −0.044)	0.317 (0.073, 0.560)	0.011	0.118
Long sleep	HTN	0.035 (−0.003, 0.072)	−0.095 (−0.188, −0.002)	0.128 (0.028, 0.228)	0.011	0.118
Long sleep	T2DM	0.101 (−0.022, 0.225)	−0.125 (−0.299, 0.049)	0.223 (0.014, 0.433)	0.036	0.273
$\hat{\gamma}_{APM}$ selection BMI-adj						
Long sleep	CKD	0.070 (−0.027, 0.168)	−0.202 (−0.425, 0.022)	0.272 (0.028, 0.516)	0.029	0.407
Long sleep	CAD	0.099 (−0.011, 0.209)	−0.146 (−0.341, 0.049)	0.241 (0.019, 0.463)	0.033	0.407
Long sleep	HTN	0.043 (0.004, 0.082)	−0.059 (−0.148, 0.029)	0.101 (0.004, 0.197)	0.041	0.407

Selected findings from tests of sex differences in causal effects. The first column describes the IV selection procedure and whether the exposure GWAS was adjusted for BMI or not. The exposure and outcome of interest are provided in the second and third columns, respectively. Sex-specific causal estimates obtained using MR-RAPS with APM variant-exposure estimates ($\hat{\gamma}_{APM}$), along with their corresponding 95% confidence intervals, are displayed in the fourth (female-specific) and fifth (male-specific) columns. The estimated causal effects are reported on the log scale, as used in the test of sex differences. The sixth column provides the estimated sex differences in causal effects, calculated from raw variant-exposure estimates ($\hat{\gamma}_{raw}$), with 95% confidence intervals. p values and FDR-adjusted p values for the statistical tests of sex differences are shown in the seventh and eighth columns. The FDR p values were computed using the Benjamini-Hochberg procedure. All the sex difference tests are significant in the nominal threshold ($p < 0.05$). IV, instrumental variable; GWAS, genome-wide association study; BMI, body mass index; BMI-unadj, BMI unadjusted; BMI-adj, BMI adjusted; MR-RAPS, MR using robust adjusted profile score method; APM, adaptive posterior mean; OR, odds ratio; OSA, obstructive sleep apnea; CAD, coronary artery disease; CKD, chronic kidney disease; HTN, hypertension; T2DM, type 2 diabetes mellitus; FDR p value, false discovery rate-adjusted p value.

MR analysis (MR-RAPS), most exposure-outcome associations retained the same direction of effect estimates. Several associations also remained statistically significant, including the increased risk of CKD associated with insomnia in females, the increased risk of CAD associated with short sleep duration in males, and the protective effects of long sleep duration on CKD and HTN in males. However, some associations, specifically in OSA analysis, were attenuated and no longer statistically significant in the MVMR analysis. For instance, the male-specific causal effect of OSA on HF was estimated at an OR of 1.30 (95% CI: 1.04–1.63) in the univariable analysis but decreased to 1.09 (95% CI: 0.82–1.45) in MVMR; the female-specific effect of OSA on T2DM was 1.32 (95% CI: 1.05–1.66) in the univariable analysis, compared to 1.13 (95% CI: 0.85–1.49) in MVMR.

Second, we compared the BMI-adjusted univariable MR analysis to the MVMR results. This time, there were a few strong differences between the results. For example, no significant associations were identified in the BMI-adjusted GWAS using the raw selection method, whereas MVMR detected several significant findings, as described above. This is not unexpected: raw selection has lower power, especially in BMI-adjusted analysis, as estimated genetic associations with the sleep exposures are weaker compared to those from BMI-unadjusted analysis. Using APM for IV selection, the causal effect of OSA on HTN in females was significant (OR: 1.14, 95% CI: 1.03–1.25) in the univariable analysis but became non-significant in MVMR (OR: 1.03, 95% CI: 0.95–1.11), and the protective effects of long sleep duration on CKD and CAD were significant in MVMR but not in the BMI-adjusted GWAS univariate MR analysis. These differences may be attributed to differences in IV selection, as well as to the approach for BMI adjustment itself. There were a few cases of differences in the directions of the estimated associations, but all associations with flipped directions between analyses were not statistically significant.

Discussion

We performed sex-specific analysis of the causal associations of sleep-related phenotypes on CVD-related outcomes. These are biologically important relationships with immediate clinical relevance. The primary rationale in our work was to ameliorate the weak instrumental bias in MR analysis. This can be done by incorporating information from auxiliary datasets. In our case, we used a male-specific dataset to improve female-specific statistics. Acknowledging likely sex differences between male and female individuals, this led to the need for an adaptive estimator, which will intelligently utilizes information across the two sex groups. Female-specific causal estimation is limited by smaller sample sizes (relative to males), leading to large variability in γ_F estimates, also known as weak IV bias. Thus, we introduced a framework to calibrate the $\hat{\gamma}_F$ estimates by borrowing information from the male group. We first demonstrated that the FE meta estimate ($\hat{\gamma}_{meta}$) is a special case of our proposed framework.

Table 4. Top findings from sex-specific exposure-outcome causal effect estimation

Exposure	Outcome	BMI adjustment	$exp\{\widehat{\beta}_{F,APM}\}$	$exp\{\widehat{\beta}_{M,APM}\}$
$\widehat{\gamma}_{raw}$ **selection**				
Insomnia	CKD	unadjusted	**1.23 (1.01, 1.49)**	1.23 (0.88, 1.73)
Long sleep	HTN	unadjusted	**1.03 (1.00, 1.06)**	0.94 (0.85, 1.05)
Short sleep	CAD	unadjusted	1.01 (0.85, 1.21)	**1.32 (1.03, 1.69)**
$\widehat{\gamma}_{APM}$ **selection**				
OSA	CKD	unadjusted	0.95 (0.77, 1.16)	**1.23 (1.03, 1.47)**
OSA	HF	unadjusted	1.07 (0.86, 1.35)	**1.30 (1.04, 1.63)**
OSA	T2DM	unadjusted	**1.32 (1.05, 1.66)**	1.09 (0.92, 1.28)
Sleepiness	AF	unadjusted	**1.18 (1.00, 1.38)**	0.99 (0.78, 1.27)
Long sleep	CAD	unadjusted	**1.11 (1.00, 1.25)**	0.80 (0.64, 1.00)
Long sleep	CKD	unadjusted	1.05 (0.95, 1.16)	**0.77 (0.61, 0.96)**
Long sleep	HTN	unadjusted	1.04 (0.99, 1.07)	**0.91 (0.83, 0.99)**
OSA	CKD	adjusted	1.09 (0.86, 1.38)	**1.27 (1.01, 1.59)**
OSA	HTN	adjusted	**1.14 (1.03, 1.25)**	0.99 (0.87, 1.14)
Insomnia	CKD	adjusted	**1.38 (1.07, 1.79)**	1.19 (0.88, 1.61)
Long sleep	HTN	adjusted	**1.04 (1.00, 1.09)**	0.94 (0.86, 1.03)

Selected findings from sex-specific exposure-outcome causal effect estimation, where significant results were identified in at least one sex-stratum, using MR-RAPS with APM variant-exposure estimates ($\widehat{\gamma}_{APM}$). The results based on $\widehat{\gamma}_{raw}$ for IV selection are shown in the top three rows, while the remaining results are from the analyses using $\widehat{\gamma}_{APM}$ for IV selection. The exposure and outcome of interest are listed in the second and third columns, respectively. The fourth column indicates whether BMI adjustment was applied in the variant-phenotype association analysis. The estimated causal effects are given in an OR scale ($exp\{\beta\}$). The female-specific causal estimates and the corresponding 95% confidence intervals are shown in the fifth column, and the results for males are in the sixth column. Bold font indicates statistically significant results ($p < 0.05$). MR-RAPS, MR using robust adjusted profile score method; APM, adaptive posterior mean; IV, instrumental variable; OR, odds ratio; OSA, obstructive sleep apnea; AF, atrial fibrillation; CAD, coronary artery disease; CKD, chronic kidney disease; HF, heart failure; HTN, hypertension; T2DM, type 2 diabetes mellitus; BMI, body mass index.

We then proposed the APM estimate, which adaptively transfers information across sex groups by considering the strength of the sex difference in $\widehat{\gamma}$ in a data-driven manner. Simulation studies demonstrated that (1) employing the shrinkage estimates can substantially improve the efficiency of causal estimation for the population with smaller sample size, (2) using the $\widehat{\gamma}_{APM}$ estimate is less sensitive to the existence of sex differences in γ compared to the use of $\widehat{\gamma}_{meta}$, and (3) no estimation efficiency is lost by applying the $\widehat{\gamma}_{APM}$ to the population with larger sample sizes. In real-data analyses, we identified several sex-difference patterns between the causal association of sleep phenotypes and CVD-related outcomes, including OSA on CKD as well as long sleep duration on several CVD-related outcomes, offering potential implications for research in sex-specific cardiovascular medicine. The method itself has broader applicability and could be used to address sex differences for a large set of complex traits. More generally, the method's utility may extend to other types of stratified, or group-specific, analysis, particularly when some similarities are expected between groups, and beyond causal association analysis. Relevant scenarios are common in both statistical genetics and biomedical research, for example, in improving the predictive performance of polygenic risk score analysis in underrepresented populations by borrowing information from genetic associations in European ancestry individuals,[45,46] improving the power to detect genetic associations with a rare subtype of a disease, by leveraging genetic associations from a common subtype,[47] and more broadly, phenotypic or other omics-based characterizations of disease subtypes when sample sizes are limited.

We also applied the proposed framework to the male population, which had a larger sample size in our analysis. The results indicate that less information could be borrowed from the smaller female population. Although the shrinkage approaches did not improve causal effect estimation for the male population in our simulations, using them did not result in a loss of estimation efficiency compared to using the raw ($\widehat{\gamma}_M$). This supports the usefulness of incorporating shrinkage approaches (especially the adaptive approach) into MR analysis when relevant summary statistics are available, regardless of the corresponding sample size.

Due to the low number of IVs that could be used when applying a genome-wide significance threshold (5×10^{-8}) for selecting IVs, we considered a lower p value threshold ($p < 10^{-5}$), as suggested by Burgess et al.,[27] as the minimal threshold value for selecting IVs in two-sample MR analysis. However, this strategy may increase the risk of including several weak instruments in MR analysis. Therefore, we applied advanced MR methods that address

weak instrumental bias, including MR-RAPS,[30] cML,[42] the contaminated mixture model,[41] and robust MR methods,[40] in simulations and in real-data analyses. We used MR-RAPS, which demonstrated superior performance in our simulation studies, as the primary method. The consistent findings between MR-RAPS and cML, the top two methods with the best estimation performance in simulation studies, also increase confidence in the data analysis findings. Additionally, to examine the influence of violating the horizontal pleiotropy assumption due to using a lower p value threshold to select IVs, we compared the causal effect estimated by MR-RAPS with MR-PRESSO,[44] another widely used approach developed for detecting horizontal pleiotropy effects, in our primary and secondary analyses. The results from MR-RAPS and MR-PRESSO are highly consistent, increasing the expected reliability of our findings using MR-RAPS.

Sleep traits, here used as exposures, are often correlated with each other,[48] as well as with other traits. High BMI is a well-known risk factor for some sleep phenotypes, particularly OSA.[49] However, the direction of causal association is often unclear. Because high BMI is known as an "upstream" risk factor (i.e., a cause) of some of the sleep traits, we performed univariable MR analyses using both BMI-adjusted and BMI-unadjusted GWASs and compared the results as part of our primary analysis, in addition to an MVMR analysis, with BMI incorporated as an exposure, in secondary analysis. There were recent reports in the MR literature that in some cases, applying two-sample MR methodology on summary statistics from a GWAS that adjusted to heritable covariates may induce bias in the estimated causal associations,[50] depending on the specific causal structure between the exposure, outcome, the covariate, and, possibly, additional unmeasured confounders. Recent papers showed that MVMR may alleviate such bias in some, but not all, settings of causal structures.[51,52] The overall patterns of our results suggested higher similarity between BMI-unadjusted univariate MR and BMI-adjusted MVMR compared to the BMI-adjusted univariate MR and either unadjusted MR or BMI-adjusted MVMR. Associations tended to be stronger and more statistically significant in the BMI-unadjusted univariate MR and the BMI-adjusted MVMR compared to the BMI-adjusted MR. Notably, in association analyses that use sleep traits as exposure and rely on individual-level data, we often see substantial differences between BMI-adjusted and -unadjusted analyses, including in recent analyses that used the OSA polygenic score in associations with CVD outcomes.[53] Therefore, it is difficult to determine which analytic approach for BMI adjustment is more reliable, especially given that the underlying causal structure is not entirely clear. Critically, in our MVMR analysis of OSA, the conditional F-statistics after accounting for BMI were all smaller than 10 in both male and female strata. In all, it is important to use these complementary analyses to account for BMI as a common cause or a confounder (it may not be a cause of all sleep traits) of sleep phenotypes and CVD outcomes. Lastly, it is important to acknowledge the potential correlation among sleep traits and the presence of other unmeasured confounders. We chose not to perform additional MVMR analysis due to the unknown causal structure between these traits, and we recommend that readers be mindful of these limitations. Additionally, complementary results from longitudinal and interventional studies are important for making strong conclusions and for ultimately guiding clinical practice.

Motivated by the need to address low female sample size in MVP data and the use of its summary statistics as an exposure GWAS in MR analysis, our focus has been on calibrating the variant-exposure effect estimates. However, we did not apply such calibrations to the outcome GWASs, as the balanced sample sizes across sex-specific GWAS in AoU suggest that further calibrations might not substantially improve the efficiency of sex-specific summary statistics, compared to the exposure GWASs. Simultaneously incorporating the proposed Bayesian framework into exposure and outcome GWASs will be of interest if both GWASs were performed with limited sample sizes.

Some limitations of this work should be discussed. First, the lack of sex-specific sleep GWASs limits the selection of IVs using independent datasets. Instead, we directly utilized the MVP sleep GWASs for the IV selection. Various IV selection strategies were implemented, including sex-specific selection, sex-specific APM estimate selection in primary analysis, and sex-combined selection by using the FE meta estimate in secondary analysis. The results, however, are only sometimes consistent across different selection methods, indicating that the estimation of causal effects is sensitive to the IV selection process. This sensitivity could be due to the strength of the IVs and differences in the underlying genetic architecture captured by the different IVs. A related concern is the limited sample size in the MVP White HARE group (European ancestry) dataset, which likely contributes to the lack of significant findings after multiple testing correction in both sex-specific and sex difference analyses. The statistical power to detect causal effects depends on the exposure GWAS sample size, which is limited in females. Notably, even within the proposed Bayesian framework, the analysis in the female stratum is limited in that information can be borrowed from the male stratum only to the extent that genetic effects appear similar between the two sexes. Thus, null findings may reflect low power. We used the summary statistics from the MVP OSA GWAS to guide the variant-exposure effect estimates in simulations to evaluate sex differences in causal effect; however, the statistical power is low (approximately 0.3–0.4) even when the true sex-specific difference in causal effect was as large as 0.1. While the real-data analysis has a larger sample size, the multiple testing burden is higher (we used a p value threshold of 0.05 in the simulations). Further, the simulation results highlight that larger sample sizes are needed for testing

for sex differences (likely due to comparison of two estimates, so that the standard error of the test statistics is high), compared to the sample sizes that we ideally need to estimate sex-specific effects with confidence (i.e., with a low enough MSE). These findings suggest that substantially larger sample sizes are necessary to reliably identify differences in causal effects between sexes, especially after correcting for multiple testing.

The second limitation arises from the test of sex differences in exposure-outcome causal effects. Our current framework of shrinkage estimates is developed to improve power for sex-specific estimates but is currently not developed for testing for sex differences. Using these estimates in tests of sex differences may result in correlated causal effect estimates between sexes and, potentially, reduced power, given that information is borrowed between sex strata. A test of sex differences that includes a covariance correction between the shrinkage estimates[32] can be exploited. However, quantifying the covariance between causal estimates across sexes using shrinkage approaches is challenging and a topic of future work. Therefore, we included only the causal estimates from the raw estimates ($\widehat{\gamma}_{F,raw}$ and $\widehat{\gamma}_{M,raw}$) in our sex differences test, where we applied the conventional two-sample t test, performed under the assumption of independence between the two compared samples.

We used only European ancestry individuals due to the larger sample sizes available. Assessing the transferability of our findings to other populations, and potentially applying a similar shrinkage framework to improve IVs in non-European ancestry populations, is of interest for future research.

While the APM method substantially improves the statistical reliability of female-specific MR analyses by stabilizing weak instruments, it does not address the possibility that different biological pathways underlie the same disease phenotype across sexes. For example, there is emerging evidence that the pathophysiology of OSA in women differs in some respects from that of OSA in men, with a lower severity in women of the physiologic endotypes causing sleep apnea, due in part to the effect of sex hormones on ventilatory drive, resulting in fewer discrete respiratory events, although often with more prolonged "shallow" respiratory event.[6,7,54] Differences in pathophysiology may be related to differences in causal effects. Thus, interpretation of causal effects in general and of those estimated via the APM framework should carefully consider potential differences in the pathophysiology of exposure traits and the potential implication of applying a Bayesian shrinkage framework when pathophysiological sex differences may be strong. Differences in pathophysiology by sex can also lead to differences in causal effect estimates when sex differences in prevalence of an outcome are small. Interpretation of such sex differences should acknowledge the potential overlap between sleep measures and other confounding factors.

Data and code availability

Summary statistics from sex-specific sleep trait GWASs will become available on the dbGaP repository "Veterans Administration (VA) MVP Summary Results from Omics Studies," study accession phs001672. The R code used to implement the proposed semi-empirical Bayes framework, two-sample MR approaches, and simulation studies is available on the GitHub repository: https://github.com/Gene-Huang/sex-specific-MR. The harmonized summary statistics for both exposure and outcome GWASs used in our data analysis are also provided in the GitHub repository.

Acknowledgments

We are grateful to the Million Veteran Program participants and staff. This research is based on data from the Million Veteran Program, Office of Research and Development, Veterans Health Administration, and was supported by the Million Veteran Program, MVP000 and BX004821. This publication does not represent the views of the Department of Veteran Affairs or the United States government. The Million Veteran Program core acknowledgments for publications are stated in Note S6. We gratefully acknowledge All of Us participants for their contributions and thank the National Institutes of Health's All of Us Research Program for making available the participant data examined in this study. The All of Us Research Program would not be possible without the partnership of its participants. The All of Us core acknowledgments for publications are stated in Note S4. This research was further supported by National Institute on Aging grant R01AG080598 and by the National Heart, Lung, and Blood Institute grant R01HL161012.

Author contributions

Conceptualization, T.S.; methodology, Y.-J.H. and T.S.; formal analysis, Y.-J.H. and N.K.; resources, project administration, and funding acquisition, K.C. and P.W.F.W.; data curation, K.C. and J.E.H.; writing – original draft, Y.-J.H. and T.S.; writing – review & editing, N.K., D.F.L., J.G., J.E.H., K.C., P.W.F.W., D.J.G., and K.M. R.; visualization, Y.-J.H.; supervision, T.S.

Declaration of interests

The authors declare no competing interests.

Supplemental information

Supplemental information can be found online at https://doi.org/10.1016/j.ajhg.2025.07.015.

Web resources

- AoU, https://www.researchallofus.org/register/
- srWGS genomic data, gs://fc-aou-datasets-controlled/v7/wgs/short_read/

Appendix A

F-statistic for IV strength quantification

We computed the *F*-statistic in both simulation studies and real-data analysis to quantify IV strength. For each variant, the *F*-

statistic was computed as $F = \frac{\widehat{\gamma}^2}{\sigma^2_{\widehat{\gamma}}}$. The average F-statistics across all selected variants was then computed as $\overline{F} = \frac{1}{p}\sum_{j=1}^{p}\frac{\widehat{\gamma}_j^2}{\sigma^2_{\widehat{\gamma},j}}$, where p denotes the total number of IVs. We also compute the F-statistic using $\widehat{\gamma}_{APM}$ and its corresponding standard error estimates for comparison. The average F-statistics from the simulation studies is presented in Table S3. For the real-data analyses, Table S5 reports the average F-statistics for each sleep exposure. Variant-level F-statistic for each exposure-outcome analysis was also computed and is available in our publicly accessible repository: https://github.com/Gene-Huang/sex-specific-MR/tree/main/harmonized_exposure_outcome_sumstat/.

Sex-specific sleep GWASs in MVP

We performed sex-specific GWASs for four sleep phenotypes (insomnia, long sleep duration, short sleep duration, and excessive daytime sleepiness) and used GWAS summary statistics from previously published GWASs of OSA[16] using MVP participants. Sleep phenotypes are described below. We only used the European (White) harmonized race/ethnicity and genetic ancestry (HARE) group to match the genetic ancestry of the available outcome sex-stratified GWASs from the AoU. We removed related individuals based on kinship coefficients $\geq$ 0.0884, where the kinship coefficients were estimated using KING v.2.0,[55] so that all analyses were conducted using an unrelated set of individuals (including participants from across the female and male strata that were unrelated). Sex chromosome checks confirmed biological sex. Variants were filtered based on imputation quality, requiring an INFO score of at least $R^2 \geq 0.6$ and a minor-allele frequency $\geq$ 0.01. Across analyses, sample sizes ranged from ~15,000 to 30,000 (female stratum) and from ~204,000 to 380,000 (male stratum). All analyses were adjusted for age and the first 10 principal components (PCs) of genetic data. For each trait, two GWASs were performed: one with and one without BMI adjustment. BMI-adjusted models had BMI as a covariate using both linear and squared terms. Analyses were performed separately using PLINK v.2.00a3LM[56] in each sex stratum. The summary of MVP sleep GWAS results is shown in Note S5.

Sleep phenotypes

Sleep phenotypes were defined as previously reported.[16] In detail, OSA was defined based on the VA electronic health record using a multimodal automated phenotyping (MAP) algorithm.[57] The MAP algorithm was applied to predict general sleep apnea and OSA separately. For each trait, it resulted in a score, roughly mapping to a likelihood of having that condition. The final OSA definition combined both scores. Insomnia status was inferred using a MAP algorithm applied to relevant ICD codes and elements extracted using natural language processing. Insomnia status was inferred at the same age as was used in the OSA analysis, i.e., a participant was considered to have insomnia if their age of first insomnia ICD code was the same or earlier than their age in the OSA analysis. All other sleep phenotypes were self-reported based on the baseline questionnaire administered to program participants. Long sleep was defined as sleep durations >9 h, and short sleep as sleep durations <6 h, where the sleep duration was defined based on the response to the question about hours of sleep in a typical day, with responses ranging from "5 or less" to "10 or more," with increments of 0.5. The excessive daytime sleepiness phenotype was based on the questionnaire item "feeling excessively sleepy during the day (does not include regular naps)," with yes or no answers. All five sleep phenotypes are binary.

MVP ethics statement

MVP received ethical/study protocol approval from the VA Central Institutional Review Board, and written informed consent was obtained for all participants.

Association analysis with CVD-related outcomes in AoU

We used short-read whole-genome sequencing (srWGS) data (version 7) from the AoU study to conduct association analysis with CVD-related outcomes. To reduce memory storage requirements, we focused on genomics data pre-filtered by the following criteria: a population-specific allele frequency $\geq$ 1% or a population-specific allele count > 100 (data are accessed via the AoU online platform using gs://fc-aou-datasets-controlled/v7/wgs/short_read/snpindel/acaf_threshold_v7.1). To align with summary statistics computed from the White HARE group in the MVP, we included only White individuals (as determined by self-reported race and ethnicity) in the AoU analysis. Related individuals were excluded based on information available via the online platform in gs://fc-aou-datasets-controlled/v7/wgs/short_read/snpindel/aux/relatedness/relatedness_flagged_samples.tsv. We also restricted the analysis to adults aged 18 to 95, with BMI values ranging from 17 to 55. Following data preprocessing, approximately 114,000 White individuals were included, consisting of 67,600 females and 46,400 males. The exact sample size varied slightly depending on the phenotype analyzed. Six binary CVD-related phenotypes were considered: atrial fibrillation (AF), coronary artery disease (CAD), cardiovascular disease (CVD), heart failure (HF), hypertension (HTN), and type 2 diabetes mellitus (T2DM). Detailed selection criteria for these clinical outcomes and the corresponding SNOMED codes and OMOP Concept IDs in the AoU study are provided in Table S7. The characteristics of AoU participants in the variant-phenotype association analysis are summarized in Table S8. More details are summarized in Note S4.

After matching the variants identified as IVs according to the exposure dataset (MVP), we conducted single-variant association analyses with the six binary outcomes. We used logistic regression, adjusting for age and 16 genetic PCs as covariates in the BMI-unadjusted analyses. For the BMI-adjusted analyses, the models were further adjusted for BMI, including both linear and quadratic terms. The effect sizes of the variants, i.e., log (OR) with the corresponding SD, were then extracted for use as summary statistics in the outcome GWAS.

MVMR analysis

We performed MVMR to estimate the direct effect of each sleep phenotype on CVD outcomes while adjusting for BMI as a secondary exposure. For both the sleep and CVD traits, we used effect estimates derived from BMI-unadjusted variant-trait associations. BMI summary statistics were downloaded from a large-scale meta-analysis of approximately 700,000 individuals of European ancestry, combining data from the GIANT consortium and the UK Biobank.[58] As sleep phenotypes were considered the primary exposures of interest, we retained the same list of IVs used in the primary analysis and extracted the corresponding BMI effect estimates for the same set of variants. For the data harmonization, we first aligned effect estimates for sleep phenotypes and BMI using the harmonise_data() function from the TwoSampleMR R package (v.0.6.8), ensuring consistency in the effect allele across exposures. Next, we harmonized both exposures with the CVD outcome using the mv_harmonise_data() function from the

same package to align all effect estimates to the same effect allele. MVMR was conducted using the robust multivariable inverse-variance weighted (MV-IVW) method,[59] and the average conditional F-statistics were computed using the strength_mvmr() function from the MVMR R package (v.0.4.1[60]).

Simulation studies

We performed simulation studies to evaluate the performance of exposure-outcome causal effect (β_F and β_M) estimation, using shrinkage variant-exposure effect estimates ($\widehat{\gamma}_{meta}$ and $\widehat{\gamma}_{APM}$) as input to existing two-sample MR approaches. We then compared these to the use of uncalibrated (raw) effect estimates ($\widehat{\gamma}_{raw}$). We employed three settings to simulate sex-specific variant-exposure effect sizes (γ_F and γ_M) and considered different levels of sex difference in causal effect within each setting.

Simulation data generation

We simulated data via the structural model presented in Figure 1A, where we use S (for sleep phenotype) to represent the exposure variable and C (for CVD phenotype) to represent the outcome variable. We first generated individual-level genetic data for two datasets, corresponding to two populations used in an exposure GWAS (one population) and an outcome GWAS (second population). In each of the datasets, the data-generating process was carried out independently in the females and males. We set the female sample size of the exposure dataset to $N_F^S = 2,000$ and the sample size of the male stratum to $N_M^S = 20,000$, matching the sex sample size proportions in the MVP. In the outcome dataset, the female and male sample sizes were set to be the same: $N_F^C = N_M^C = 10,000$. For each genetic variant, allele counts were generated independently from $Binomial(2, 0.3)$ distribution, assuming that all variants are in linkage equilibrium with allele frequencies of 0.3.

In the exposure dataset, we generated the exposure variable according to the following linear model:

$$S_{Fi} = 1 + \sum_{j=1}^{p} \gamma_{jF} \times g_{Fji} + \alpha \times U_{Fi} + \varepsilon_{Fi}, i = 1, 2, \ldots, N_F^S \text{ and}$$

(Equation A1)

$$S_{Mi} = 1 + \sum_{j=1}^{p} \gamma_{Mj} \times g_{Mji} + \alpha \times U_{Mi} + \varepsilon_{Mi}, i = 1, 2, \ldots, N_M^S,$$

(Equation A2)

with $p = 100$ independent variants. U represents the unknown confounder, which we generated from normal distribution with mean 0 but different variances in female and male populations: $U_{Fi} \sim N(0, 1)$ and $U_{Mi} \sim N(0, 0.5)$. The random errors ε_{Fi} and ε_{Mi} were generated from standard normal distribution for both the female and male populations. The γ_{jF} and γ_{Mj} are the underlying sex-specific variant-exposure effect sizes. The effect size of the unknown confounder to the exposure association was set as $\alpha = 0.1$ for both the female and male populations.

Next, we use the same strategy to generate the genetic data, exposure variable, and unmeasured confounder for the outcome population,

$$S_{Fi}^O = 1 + \sum_{j=1}^{p} \gamma_{jF} \times g_{Fji}^O + \alpha \times U_{Fi}^O + \varepsilon_{Fi}^O, i = 1, 2, \ldots, N_F^C \text{ and}$$

(Equation A3)

$$S_{Mi}^O = 1 + \sum_{j=1}^{p} \gamma_{Mj} \times g_{Mji}^O + \alpha \times U_{Mi}^O + \varepsilon_{Mi}^O, i = 1, 2, \ldots, N_M^C.$$

(Equation A4)

The superscript O is used to distinguish the exposure simulated in the outcome population from the exposure simulated in the exposure population. Based on the generated exposure variables S_{Fi}^O and S_{Mi}^O, we then generate the outcome variable through the following linear equation:

$$C_{Fi} = 1 + \beta_F \times S_{Fi}^O + \phi \times U_{Fi}^O + \varepsilon_{Fi}^C, i = 1, 2, \ldots, N_F^C \text{ and}$$

(Equation A5)

$$C_{Mi} = 1 + \beta_M \times S_{Mi}^O + \phi \times U_{Mi}^O + \varepsilon_{Mi}^C, i = 1, 2, \ldots, N_M^C,$$

(Equation A6)

where ϕ denotes the effect sizes of the unknown confounder on the outcome, which we set to $\phi = 0.1$. The β_F and β_M are the underlying true female and male exposure-outcome causal effects, respectively.

After generating the individual-level data, we get the exposure GWAS and outcome GWAS summary statistics by fitting marginal linear regression models. The exposure data summary statistics $(\widehat{\gamma}_{jF}, \widehat{\sigma}^2_{\gamma,jF})$ and $(\widehat{\gamma}_{jM}, \widehat{\sigma}^2_{\gamma,jM}), j = 1, 2, \ldots, 100$ are obtained by fitting the following regression using the data generated from Equations A1 and A2:

$$E(S_F) = \tilde{\gamma}_{F0} + \tilde{\gamma}_{jF} \times g_{Fj}, j = 1, 2, \ldots, 100 \quad \text{and}$$

$$E(S_M) = \tilde{\gamma}_{M0} + \tilde{\gamma}_{jM} \times g_{Mj}, j = 1, 2, \ldots, 100,$$

and the same procedure is applied for the outcome population

$$E(C_F) = \Gamma_{F0} + \Gamma_{Fj} \times g_{Fj}^O, j = 1, 2, \ldots, 100 \quad \text{and}$$

$$E(C_M) = \Gamma_{M0} + \Gamma_{Mj} \times g_{Mj}^O, j = 1, 2, \ldots, 100.$$

The outcome GWAS summary statistics are $(\widehat{\Gamma}_{jF}, \widehat{\sigma}^2_{\Gamma,jF})$ and $(\widehat{\Gamma}_{jM}, \widehat{\sigma}^2_{\Gamma,jM}), j = 1, 2, \ldots, 100$.

We performed simulations in three simulation settings that differed in the way that variant-exposure effect sizes were simulated. In all settings, we considered a few models for the exposure-outcome effect sizes: no sex differences in exposure-outcome causal effects, with $\beta_F = \beta_M = 0.1$, and sex differences in exposure-outcome causal effect with $\beta_M = 0.1$, and $\beta_F \in \{0, 0.05, 0.15\}$.

Simulation settings 1: Fixed sex differences in variant-exposure effects

Here, we simulated from settings in which the sex differences in γ_F and γ_M, when there were such differences, were weak and fixed across variants. Male-specific variant-exposure effect sizes were $\gamma_{jM} = 0.1, j = 1, 2, \ldots, 100$ for all variants. For the female population, some variants had the same effect size as in the male population ($\gamma_{jF} = 0.1$), and a subset of variants (sized 10, 50, or 90 out of 100) had female-specific variant-exposure effect size $\gamma_{jF} = 0.05$. For the sex differences in the causal effect setting, we fixed 50% of variants to have sex differences in variant-exposure effect sizes.

Simulation settings 2: Random sex differences in variant-exposure effects

Here, we simulated from settings in which the sex differences in variant-exposure effect sizes, when there were such differences, were strong. For each variant $j = 1, 2, \ldots, 100$, we generated a pair of female and male variant-exposure effect sizes from a bivariate normal distribution $\begin{pmatrix} \gamma_{jF} \\ \gamma_{jM} \end{pmatrix} \sim N_2\left(\begin{pmatrix} 0.1 \\ 0.1 \end{pmatrix}, \quad \Sigma = \begin{bmatrix} 0.01 & \phi \\ \phi & 0.01 \end{bmatrix}\right)$, where we considered different covariance value ϕ to set the correlations ($\frac{\phi}{0.01}$) between (γ_F, γ_M) to equal 0.5, 0.7, or 0.9 when the exposure-outcome causal effect was the same in males and females ($\beta_F = \beta_M = 0.1$). When $\beta_M \neq \beta_F$, the correlation between (γ_F, γ_M) was set to 0.7. Finally, the variant exposure effect sizes γ_F, γ_M were randomly generated in each simulation replicate.

Simulation settings 3: Summary statistics from MVP OSA GWAS guide variant-exposure effect sizes

Here, we used the summary statistics from the MVP OSA GWAS (without BMI adjustment) to guide the γ_F and γ_M values. First, we conducted p value thresholding and clumping for the male-specific summary statistics. The p value threshold was set at 10^{-5}, with a clumping window of 10,000 kb and a correlation threshold of 0.001, using the European population reference panel from 1000 Genomes. This resulted in a list of 110 variants. We randomly selected 100 variants used their male- and female-specific effect size estimates from the MVP GWAS in simulations.

Two-sample MR methods

We applied the following two-sample MR methods, described in Table 2, over the raw and calibrated estimated variant effect sizes.

Received: June 16, 2025
Accepted: July 25, 2025
Published: September 4, 2025

References

1. Khramtsova, E.A., Davis, L.K., and Stranger, B.E. (2019). The role of sex in the genomics of human complex traits. Nat. Rev. Genet. *20*, 173–190. https://doi.org/10.1038/s41576-018-0083-1.
2. Mauvais-Jarvis, F., Bairey Merz, N., Barnes, P.J., Brinton, R.D., Carrero, J.-J., DeMeo, D.L., De Vries, G.J., Epperson, C.N., Govindan, R., Klein, S.L., et al. (2020). Sex and gender: Modifiers of health, disease, and medicine. Lancet *396*, 565–582. https://doi.org/10.1016/S0140-6736(20)31561-0.
3. Ji, H., Niiranen, T.J., Rader, F., Henglin, M., Kim, A., Ebinger, J.E., Claggett, B., Merz, C.N.B., and Cheng, S. (2021). Sex differences in blood pressure associations with cardiovascular outcomes. Circulation *143*, 761–763. https://doi.org/10.1161/CIRCULATIONAHA.120.049360.
4. Zeng, L.-N., Zong, Q.-Q., Yang, Y., Zhang, L., Xiang, Y.-F., Ng, C.H., Chen, L.-G., and Xiang, Y.-T. (2020). Gender difference in the prevalence of insomnia: A meta-analysis of observational studies. Front. Psychiatry *11*, 577429. https://doi.org/10.3389/fpsyt.2020.577429.
5. Dib, R., Gervais, N.J., and Mongrain, V. (2021). A review of the current state of knowledge on sex differences in sleep and circadian phenotypes in rodents. Neurobiol. Sleep Circadian Rhythms *11*, 100068. https://doi.org/10.1016/j.nbscr.2021.100068.
6. Bonsignore, M.R., Saaresranta, T., and Riha, R.L. (2019). Sex differences in obstructive sleep apnoea. Eur. Respir. Rev. *28*, 190030. https://doi.org/10.1183/16000617.0030-2019.
7. Won, C.H.J., Reid, M., Sofer, T., Azarbarzin, A., Purcell, S., White, D., Wellman, A., Sands, S., and Redline, S. (2020). Sex differences in obstructive sleep apnea phenotypes, the multi-ethnic study of atherosclerosis. Sleep *43*, zsz274. https://doi.org/10.1093/sleep/zsz274.
8. Millett, E.R.C., Peters, S.A.E., and Woodward, M. (2018). Sex differences in risk factors for myocardial infarction: Cohort study of UK Biobank participants. BMJ *363*, k4247. https://doi.org/10.1136/bmj.k4247.
9. Connelly, P.J., Currie, G., and Delles, C. (2022). Sex differences in the prevalence, outcomes and management of hypertension. Curr. Hypertens. Rep. *24*, 185–192. https://doi.org/10.1007/s11906-022-01183-8.
10. Schulte, K.J., and Mayrovitz, H.N. (2023). Myocardial Infarction Signs and Symptoms: Females vs. Males. Cureus *15*, e37522. https://doi.org/10.7759/cureus.37522.
11. Kasimatis, K.R., Abraham, A., Ralph, P.L., Kern, A.D., Capra, J. A., and Phillips, P.C. (2021). Evaluating human autosomal loci for sexually antagonistic viability selection in two large biobanks. Genetics *217*, 1–10. https://doi.org/10.1093/genetics/iyaa015.
12. Pirastu, N., Cordioli, M., Nandakumar, P., Mignogna, G., Abdellaoui, A., Hollis, B., Kanai, M., Rajagopal, V.M., Parolo, P. D.B., Baya, N., et al. (2021). Genetic analyses identify widespread sex-differential participation bias. Nat. Genet. *53*, 663–671. https://doi.org/10.1038/s41588-021-00846-7.
13. Huang, Y., Shan, Y., Zhang, W., Lee, A.M., Li, F., Stranger, B. E., and Huang, R.S. (2023). Deciphering genetic causes for sex differences in human health through drug metabolism and transporter genes. Nat. Commun. *14*, 175. https://doi.org/10.1038/s41467-023-35808-6.
14. Pulit, S.L., Stoneman, C., Morris, A.P., Wood, A.R., Glastonbury, C.A., Tyrrell, J., Yengo, L., Ferreira, T., Marouli, E., Ji, Y., et al. (2019). Meta-analysis of genome-wide association studies for body fat distribution in 694649 individuals of European ancestry. Hum. Mol. Genet. *28*, 166–174. https://doi.org/10.1093/hmg/ddy327.
15. Huang, Y., Hui, Q., Gwinn, M., Hu, Y.-J., Quyyumi, A.A., Vaccarino, V., and Sun, Y.V. (2021). Sexual differences in genetic predisposition of coronary artery disease. Circ. Genom. Precis. Med. *14*, e003147. https://doi.org/10.1161/CIRCGEN.120.003147.
16. Sofer, T., Kurniansyah, N., Murray, M., Ho, Y.-L., Abner, E., Esko, T., Estonian Biobank Research Team, Huffman, J.E., Cho, K., Mägi, R., and et al.. (2023). Genome-wide association study of obstructive sleep apnoea in the Million Veteran Program uncovers genetic heterogeneity by sex. EBioMedicine *90*, 104536. https://doi.org/10.1016/j.ebiom.2023.104536.
17. Koch, E., Nyberg, L., Lundquist, A., Pudas, S., Adolfsson, R., and Kauppi, K. (2021). Sex-specific effects of polygenic risk for schizophrenia on lifespan cognitive functioning in healthy individuals. Transl. Psychiatry *11*, 520. https://doi.org/10.1038/s41398-021-01649-4.
18. Gui, Y., Zhou, X., Wang, Z., Zhang, Y., Wang, Z., Zhou, G., Zhao, Y., Liu, M., Lu, H., and Zhao, H. (2022). Sex-specific genetic association between psychiatric disorders and

cognition, behavior and brain imaging in children and adults. Transl. Psychiatry *12*, 347. https://doi.org/10.1038/s41398-022-02041-6.
19. Zhang, C., Ye, Y., and Zhao, H. (2022). Comparison of methods utilizing sex-specific PRSs derived from GWAS summary statistics. Front. Genet. *13*, 892950. https://doi.org/10.3389/fgene.2022.892950.
20. Mayne, B.T., Bianco-Miotto, T., Buckberry, S., Breen, J., Clifton, V., Shoubridge, C., and Roberts, C.T. (2016). Large scale gene expression meta-analysis reveals tissue-specific, sex-biased gene expression in humans. Front. Genet. *7*, 183. https://doi.org/10.3389/fgene.2016.00183.
21. Oliva, M., Muñoz-Aguirre, M., Kim-Hellmuth, S., Wucher, V., Gewirtz, A.D.H., Cotter, D.J., Parsana, P., Kasela, S., Balliu, B., Viñuela, A., et al. (2020). The impact of sex on gene expression across human tissues. Science *369*, eaba3066. https://doi.org/10.1126/science.aba3066.
22. Wingo, A.P., Liu, Y., Gerasimov, E.S., Vattathil, S.M., Liu, J., Cutler, D.J., Epstein, M.P., Blokland, G.A.M., Thambisetty, M., Troncoso, J.C., et al. (2023). Sex differences in brain protein expression and disease. Nat. Med. *29*, 2224–2232. https://doi.org/10.1038/s41591-023-02509-y.
23. Smith, G.D., and Ebrahim, S. (2003). 'Mendelian randomization': Can genetic epidemiology contribute to understanding environmental determinants of disease? Int. J. Epidemiol. *32*, 1–22. https://doi.org/10.1093/ije/dyg070.
24. Zhao, J.V., Luo, S., and Schooling, C.M. (2019). Sex-specific Mendelian randomization study of genetically predicted insulin and cardiovascular events in the UK Biobank. Commun. Biol. *2*, 332. https://doi.org/10.1038/s42003-019-0579-z.
25. Mielke, M.M., and Miller, V.M. (2021). Improving clinical outcomes through attention to sex and hormones in research. Nat. Rev. Endocrinol. *17*, 625–635. https://doi.org/10.1038/s41574-021-00531-z.
26. Ardissino, M., Slob, E.A.W., Carter, P., Rogne, T., Girling, J., Burgess, S., and Ng, F.S. (2023). Sex-specific reproductive factors augment cardiovascular disease risk in women: a mendelian randomization study. J. Am. Heart Assoc. *12*, e027933. https://doi.org/10.1161/JAHA.122.027933.
27. Burgess, S., Butterworth, A., and Thompson, S.G. (2013). Mendelian randomization analysis with multiple genetic variants using summarized data. Genet. Epidemiol. *37*, 658–665. https://doi.org/10.1002/gepi.21758.
28. Holmes, M.V., Ala-Korpela, M., and Smith, G.D. (2017). Mendelian randomization in cardiometabolic disease: Challenges in evaluating causality. Nat. Rev. Cardiol. *14*, 577–590. https://doi.org/10.1038/nrcardio.2017.78.
29. Slob, E.A.W., and Burgess, S. (2020). A comparison of robust Mendelian randomization methods using summary data. Genet. Epidemiol. *44*, 313–329. https://doi.org/10.1002/gepi.22295.
30. Zhao, Q., Wang, J., Hemani, G., Bowden, J., and Small, D.S. (2020). Statistical inference in two-sample summary-data Mendelian randomization using robust adjusted profile score. Ann. Statist. *48*. https://doi.org/10.1214/19-AOS1866.
31. Ye, T., Shao, J., and Kang, H. (2021). Debiased inverse-variance weighted estimator in two-sample summary-data Mendelian randomization. Ann. Statist. *49*, 2079–2100. https://doi.org/10.1214/20-AOS2027.
32. Bernabeu, E., Canela-Xandri, O., Rawlik, K., Talenti, A., Prendergast, J., and Tenesa, A. (2021). Sex differences in genetic architecture in the UK biobank. Nat. Genet. *53*, 1283–1289. https://doi.org/10.1038/s41588-021-00912-0.
33. Zhu, C., Ming, M.J., Cole, J.M., Edge, M.D., Kirkpatrick, M., and Harpak, A. (2023). Amplification is the primary mode of gene-by-sex interaction in complex human traits. Cell Genom. *3*, 100297. https://doi.org/10.1016/j.xgen.2023.100297.
34. Mukherjee, B., and Chatterjee, N. (2008). Exploiting gene-environment independence for analysis of case–control studies: An empirical bayes-type shrinkage estimator to trade-off between bias and efficiency. Biometrics *64*, 685–694. https://doi.org/10.1111/j.1541-0420.2007.00953.x.
35. Li, H., Gail, M.H., Berndt, S., and Chatterjee, N. (2010). Using cases to strengthen inference on the association between single nucleotide polymorphisms and a secondary phenotype in genome-wide association studies. Genet. Epidemiol. *34*, 427–433. https://doi.org/10.1002/gepi.20495.
36. Li, H., and Gail, M.H. (2012). Efficient adaptively weighted analysis of secondary phenotypes in case-control genome-wide association studies. Hum. Hered. *73*, 159–173. https://doi.org/10.1159/000338943.
37. Domínguez Islas, C., and Rice, K.M. (2022). Bayesian approaches to fixed effects META-ANALYSIS. Res. Synth. Methods *13*, 520–532. https://doi.org/10.1002/jrsm.1566.
38. Lin, D.Y., and Zeng, D. (2010). Meta-analysis of genome-wide association studies: No efficiency gain in using individual participant data. Genet. Epidemiol. *34*, 60–66. https://doi.org/10.1002/gepi.20435.
39. Bowden, J., Davey Smith, G., and Burgess, S. (2015). Mendelian randomization with invalid instruments: Effect estimation and bias detection through Egger regression. Int. J. Epidemiol. *44*, 512–525. https://doi.org/10.1093/ije/dyv080.
40. Rees, J.M.B., Wood, A.M., Dudbridge, F., and Burgess, S. (2019). Robust methods in Mendelian randomization via penalization of heterogeneous causal estimates. PLoS One *14*, e0222362. https://doi.org/10.1371/journal.pone.0222362.
41. Burgess, S., Foley, C.N., Allara, E., Staley, J.R., and Howson, J.M.M. (2020). A robust and efficient method for Mendelian randomization with hundreds of genetic variants. Nat. Commun. *11*, 376. https://doi.org/10.1038/s41467-019-14156-4.
42. Xue, H., Shen, X., and Pan, W. (2021). Constrained maximum likelihood-based Mendelian randomization robust to both correlated and uncorrelated pleiotropic effects. Am. J. Hum. Genet. *108*, 1251–1269. https://doi.org/10.1016/j.ajhg.2021.05.014.
43. Bowden, J., Davey Smith, G., Haycock, P.C., and Burgess, S. (2016). Consistent estimation in mendelian randomization with some invalid instruments using a weighted median estimator. Genet. Epidemiol. *40*, 304–314. https://doi.org/10.1002/gepi.21965.
44. Verbanck, M., Chen, C.-Y., Neale, B., and Do, R. (2018). Detection of widespread horizontal pleiotropy in causal relationships inferred from Mendelian randomization between complex traits and diseases. Nat. Genet. *50*, 693–698. https://doi.org/10.1038/s41588-018-0099-7.
45. Hoggart, C.J., Choi, S.W., García-González, J., Souaiaia, T., Preuss, M., and O'Reilly, P.F. (2024). BridgePRS leverages shared genetic effects across ancestries to increase polygenic risk score portability. Nat. Genet. *56*, 180–186. https://doi.org/10.1038/s41588-023-01583-9.
46. Irvin, M.R., Ge, T., Patki, A., Srinivasasainagendra, V., Armstrong, N.D., Davis, B., Jones, A.C., Perez, E., Stalbow, L., Lebo, M., et al. (2024). Polygenic risk for type 2 diabetes in

African Americans. Diabetes *73*, 993–1001. https://doi.org/10.2337/db23-0232.
47. Henry, A., Mo, X., Finan, C., Chaffin, M.D., Speed, D., Issa, H., Denaxas, S., Ware, J.S., Zheng, S.L., Malarstig, A., et al. (2025). Genome-wide association study meta-analysis provides insights into the etiology of heart failure and its subtypes. Nat. Genet. *57*, 815–828. https://doi.org/10.1038/s41588-024-02064-3.
48. Chung, J., Goodman, M., Huang, T., Bertisch, S., and Redline, S. (2021). Multidimensional sleep health in a diverse, aging adult cohort: Concepts, advances, and implications for research and intervention. Sleep Health *7*, 699–707. https://doi.org/10.1016/j.sleh.2021.08.005.
49. Punjabi, N.M. (2008). The epidemiology of adult obstructive sleep apnea. Proc. Am. Thorac. Soc. *5*, 136–143. https://doi.org/10.1513/pats.200709-155MG.
50. Hartwig, F.P., Tilling, K., Davey Smith, G., Lawlor, D.A., and Borges, M.C. (2021). Bias in two-sample Mendelian randomization when using heritable covariable-adjusted summary associations. Int. J. Epidemiol. *50*, 1639–1650. https://doi.org/10.1093/ije/dyaa266.
51. Gilbody, J., Borges, M.C., Davey Smith, G., and Sanderson, E. (2025). Multivariable MR can mitigate bias in two-sample MR using covariable-adjusted summary associations. Genet. Epidemiol. *49*, e22606. https://doi.org/10.1002/gepi.22606.
52. Wang, P., Lin, Z., and Pan, W. (2025). Unbiased causal inference with Mendelian randomization and covariate-adjusted GWAS data. HGG Adv. *6*, 100412. https://doi.org/10.1016/j.xhgg.2025.100412.
53. Kurniansyah, N., Strausz, S.J., Chittoor, G., Gupta, S., Justice, A.E., Hrytsenko, Y., Keenan, B.T., Cade, B.E., Spitzer, B.W., Wang, H., et al. (2025). Polygenic scores for obstructive sleep apnoea reveal pathways contributing to cardiovascular disease. EBioMedicine *117*, 105790. https://doi.org/10.1016/j.ebiom.2025.105790.
54. Borker, P.V., Reid, M., Sofer, T., Butler, M.P., Azarbarzin, A., Wang, H., Wellman, A., Sands, S.A., and Redline, S. (2021). Non-REM apnea and hypopnea duration varies across population groups and physiologic traits. Am. J. Respir. Crit. Care Med. *203*, 1173–1182. https://doi.org/10.1164/rccm.202005-1808OC.
55. Manichaikul, A., Mychaleckyj, J.C., Rich, S.S., Daly, K., Sale, M., and Chen, W.-M. (2010). Robust relationship inference in genome-wide association studies. Bioinformatics *26*, 2867–2873. https://doi.org/10.1093/bioinformatics/btq559.
56. Chang, C.C., Chow, C.C., Tellier, L.C., Vattikuti, S., Purcell, S. M., and Lee, J.J. (2015). Second-generation PLINK: Rising to the challenge of larger and richer datasets. GigaScience *4*, 7. https://doi.org/10.1186/s13742-015-0047-8.
57. Liao, K.P., Sun, J., Cai, T.A., Link, N., Hong, C., Huang, J., Huffman, J.E., Gronsbell, J., Zhang, Y., Ho, Y.-L., et al. (2019). High-throughput multimodal automated phenotyping (MAP) with application to PheWAS. J. Am. Med. Inform. Assoc. *26*, 1255–1262. https://doi.org/10.1093/jamia/ocz066.
58. Yengo, L., Sidorenko, J., Kemper, K.E., Zheng, Z., Wood, A.R., Weedon, M.N., Frayling, T.M., Hirschhorn, J., Yang, J., Visscher, P.M.; and GIANT Consortium (2018). Meta-analysis of genome-wide association studies for height and body mass index in ∼700000 individuals of European ancestry. Hum. Mol. Genet. *27*, 3641–3649. https://doi.org/10.1093/hmg/ddy271.
59. Grant, A.J., and Burgess, S. (2021). Pleiotropy robust methods for multivariable Mendelian randomization. Stat. Med. *40*, 5813–5830. https://doi.org/10.1002/sim.9156.
60. Sanderson, E., Spiller, W., and Bowden, J. (2021). Testing and correcting for weak and pleiotropic instruments in two-sample multivariable Mendelian randomization. Stat. Med. *40*, 5434–5452. https://doi.org/10.1002/sim.9133.

The American Journal of Human Genetics, Volume *112*

Supplemental information

A semi-empirical Bayes approach

for calibrating weak instrumental bias

in sex-specific Mendelian randomization studies

Yu-Jyun Huang, Nuzulul Kurniansyah, Daniel F. Levey, Joel Gelernter, Jennifer E. Huffman, Kelly Cho, Peter W.F. Wilson, Daniel J. Gottlieb, Kenneth M. Rice, and Tamar Sofer

Note S1: Primary simulation studies

Overview of simulation settings and summary of primary results

To mimic the female-to-male sample size proportions in the Million Veteran Program (MVP), where we used the corresponding GWAS summary statistics as the exposure GWAS in two-sample Mendelian randomization (MR) analyses, we set the female exposure population sample size to 2,000 and the male exposure population to 20,000. For simplicity, we balanced the sample sizes for the outcome phenotype population between females and males, setting both to 10,000. In these simulation studies, the smaller sample size for females may introduce weak instrumental variable (IV) bias, potentially affecting the accuracy of female-specific exposure-outcome causal effect estimates ($\hat{\beta}_F$). As in the main manuscript, in what follows, we use γ to represent "variant-exposure" effect size and Γ to represent "variant-outcome" effect size. We evaluated using the shrinkage variant-exposure effect estimates ($\hat{\gamma}_{meta}, \hat{\gamma}_{APM}, \hat{\gamma}_{AW}$) for estimating the causal effect of interest (β_F), and compared it to the use of uncalibrated, raw, estimates ($\hat{\gamma}_{raw}$). In all the simulations, we considered 100 independent variants as IVs, all with allele frequency equal to 0.3. The results from simulation studies are summarized over 1000 simulation replicates.

We conducted simulations in a few settings. First, we compared sex differences in the exposure-outcome causal effect settings: no sex differences ($\beta_F = \beta_M$) and sex differences ($\beta_F \neq \beta_M$). For the sex difference setting, we fixed $\beta_M = 0.1$ and varied the strength of β_F ($\beta_F = 0, 0.05, 0.15$). In settings with no sex differences in the exposure-outcome causal effects, we set $\beta_F = \beta_M = 0.1$, and considered different numbers of variants having sex differences in γ (notation $|D_\gamma|$ in

main manuscript), as well as different strengths of sex differences effect in γ. The details of the simulation settings are summarized in Tables S1 and S2. The average *F*-statistic computed using $\hat{\gamma}_{raw}$ and $\hat{\gamma}_{APM}$ are shown in Table S3. The full results, including both female- and male-specific estimation performance, are presented in Figures S1 (MSE of causal effect estimate) and S2 (95% confidence interval coverage rate).

Table S1: Summary of primary simulation results

Causal effect estimation	Sex differences setting	MSE results	95% CI coverage results
Estimation of β_F	No sex differences in causal effect settings	• $\hat{\gamma}_{meta}$ performs the best when (i): there are few variants with sex differences in γ ($\lvert D_\gamma \rvert = 10$); (ii) the correlation between γ_F and γ_M is strong (cor = 0.9)<br>• $\hat{\gamma}_{APM}$ outperforms $\hat{\gamma}_{raw}$ in most of the settings<br>• MR-RAPS and cML have smaller MSEs compared to other MR methods in most settings	• $\hat{\gamma}_{APM}$ has a similar coverage rate compared to $\hat{\gamma}_{raw}$<br>• $\hat{\gamma}_{APM}$ improves the coverage rate in settings with large causal effect settings ($\beta_M = \beta_F = 0.2$)<br>• The coverage rate of MR-RAPS is closest to the nominal level (95%) among all MR methods
	Sex differences in causal effect settings	• $\hat{\gamma}_{APM}$ improves the estimation performance over γ_{raw} in the non-null causal effect setting ($\beta_F \neq 0$), and performs similarly to γ_{raw} in the null causal effect setting ($\beta_F = 0$)<br>• MR-RAPS and cML have smaller MSEs compared to other MR methods in most settings	• $\hat{\gamma}_{APM}$ improved the coverage rate in the settings where the causal effect is higher in females than males ($\beta_F = 0.15;\ \beta_M = 0.1$)<br>• The coverage rate of MR-RAPS is closest to the nominal level (95%) among all MR methods
Estimation of β_M	No sex differences in causal effect setting	• $\hat{\gamma}_{APM}$, $\hat{\gamma}_{AW}$, and $\hat{\gamma}_{meta}$ have similar performance compared to the $\hat{\gamma}_{raw}$<br>• MR-RAPS and cML perform slightly better than other MR methods	• $\hat{\gamma}_{APM}$, $\hat{\gamma}_{AW}$, and $\hat{\gamma}_{meta}$ have a similar coverage rate compared to $\hat{\gamma}_{raw}$<br>• The coverage rate of MR-RAPS is closest to the nominal level (95%) among all MR methods
	Sex differences in causal effect setting	• $\hat{\gamma}_{APM}$, $\hat{\gamma}_{AW}$, and $\hat{\gamma}_{meta}$ have similar performance compared to $\hat{\gamma}_{raw}$<br>• MR-RAPS and cML perform slightly better than other MR methods	• $\hat{\gamma}_{APM}$, $\hat{\gamma}_{AW}$, and $\hat{\gamma}_{meta}$ have a similar coverage rate compared to $\hat{\gamma}_{raw}$<br>• The coverage rate of MR-RAPS is closest to the nominal level (95%) among all MR methods

D_γ denotes the set containing variants with sex differences in variant-exposure effect sizes.
Abbreviation: MSE: mean square error; CI: confidence interval; cor: correlation; MR: Mendelian randomization; APM: adaptive posterior mean.

Table S2: Summary of primary simulation settings

**Simulation (i): fixed sex differences in variant-exposure effect sizes**						
	No sex differences in causal effect setting			Sex differences in causal effect setting		
exposure-outcome causal effect	$\beta_F = \beta_M = 0.1$			$\beta_F = 0$<br>$\beta_M = 0.1$	$\beta_F = 0.05$<br>$\beta_M = 0.1$	$\beta_F = 0.15$<br>$\beta_M = 0.1$
variant-exposure effect sizes	$\gamma_{jM} = 0.1, j = 1,2, \ldots, 100$<br>$\gamma_{jF} = \begin{cases} 0.05, j \in D_\gamma \\ 0.1, j \notin D_\gamma \end{cases}$			$\gamma_{jM} = 0.1, j = 1,2, \ldots, 100$<br>$\gamma_{jF} = \begin{cases} 0.05, j \in D_\gamma \\ 0.1, j \notin D_\gamma \end{cases}$		
Number of variants having sex differences in variant-exposure effect sizes	$\lvert D_\gamma \rvert = 10$	$\lvert D_\gamma \rvert = 50$	$\lvert D_\gamma \rvert = 90$	$\lvert D_\gamma \rvert = 50$		
**Simulation (ii): random sex differences in variant-exposure effect sizes**						
	No sex differences in causal effect setting			Sex differences in causal effect setting		
exposure-outcome causal effect	$\beta_F = \beta_M = 0.1$			$\beta_F = 0$<br>$\beta_M = 0.1$	$\beta_F = 0.05$<br>$\beta_M = 0.1$	$\beta_F = 0.15$<br>$\beta_M = 0.1$
variant-exposure effect sizes	$\begin{pmatrix} \gamma_{jF} \\ \gamma_{jM} \end{pmatrix} \sim N_2\left(\begin{pmatrix} 0.1 \\ 0.1 \end{pmatrix}, \Sigma^2\right), j = 1,2, \ldots 100$			$\begin{pmatrix} \gamma_{jF} \\ \gamma_{jM} \end{pmatrix} \sim N_2\left(\begin{pmatrix} 0.1 \\ 0.1 \end{pmatrix}, \Sigma^2\right), j = 1,2, \ldots 100$		
Correlation of $(\gamma_{jF}, \gamma_{jM})$	0.5	0.7	0.9	0.7		
**Simulation (iii): Use MVP OSA GWAS summary statistics to guide variant-exposure effect sizes**						
	No sex differences in causal effect setting			Sex differences in causal effect setting		
exposure-outcome causal effect	$\beta_F = \beta_M = 0$	$\beta_F = \beta_M = 0.1$	$\beta_F = \beta_M = 0.2$	$\beta_F = 0$<br>$\beta_M = 0.1$	$\beta_F = 0.05$<br>$\beta_M = 0.1$	$\beta_F = 0.15$<br>$\beta_M = 0.1$
variant-exposure effect sizes	From MVP OSA bmi-unadjusted GWAS			From MVP OSA bmi-unadjusted GWAS		

D_γ denotes the set containing variants defined as sex differences in variant-exposure effect sizes. For simulation (ii), the sex-specific variant-exposure effect sizes were simulated from a bivariate normal distribution with the mean value set at 0.1 and the variance set at 0.01 for both sex groups

Table S3: Average *F*-statistic from primary simulation settings

	$\hat{\gamma}_{F,raw}$	$\hat{\gamma}_{F,APM}$	$\hat{\gamma}_{M,raw}$	$\hat{\gamma}_{M,APM}$
Simulation (i)				
$\lvert D_\gamma \rvert = 10$	6.6 (0.5)	23.8 (1.9)	60.3 (1.5)	64.1 (1.6)
$\lvert D_\gamma \rvert = 50$	5.1 (0.4)	19.1 (1.8)	60.3 (1.4)	63.8 (1.5)
$\lvert D_\gamma \rvert = 90$	3.4 (0.3)	13.4 (1.6)	60.2 (1.5)	63.3 (1.6)
Simulation (ii)				
$cor = 0.5$	10.2 (0.8)	22.2 (3.5)	92.8 (6.3)	96.0 (6.6)
$cor = 0.7$	10.1 (0.8)	25.3 (4.1)	92.5 (6.4)	96.4 (6.8)
$cor = 0.9$	10.1 (0.8)	31.5 (4.9)	92.9 (6.3)	97.9 (6.7)
Simulation (iii)	1.9 (0.2)	4.3 (0.6)	16.7 (0.8)	17.4 (0.8)

Note: These results represent the average *F*-statistic computed across variants (100 variants) and across simulation repetitions (1000 repetitions). The standard deviation of average *F*-statistic across simulation repetitions is shown in the parentheses.

Figure S1: MSE of sex-specific causal effect estimates from primary simulation studies

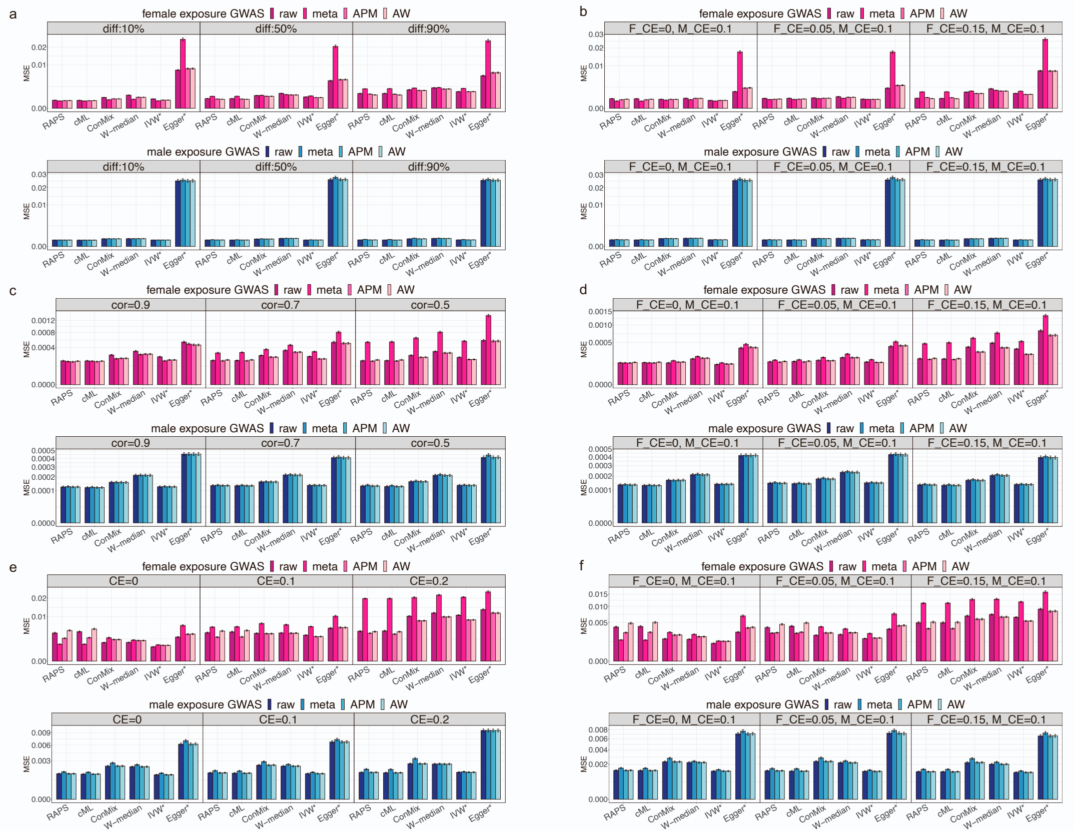

This figure shows the MSE of estimated β_F and β_M. Female results are in pink, and male results are in blue. Two-sample MR methods are annotated as x-axis labels. Shade of bars correspond to the type of variant-exposure effect estimate (raw, meta, APM, AW), as labeled. Panels a and b provide results from simulation settings with fixed sex differences in γ, panels c and d correspond to settings with random sex differences in γ, and panels e and f correspond to settings with MVP OSA GWAS-guided sex-specific γ effect sizes. Left panels (a, c, e) corresponds to settings with $\beta_F = \beta_M$ (CE), while the right panels (b, d, f) correspond to settings with $\beta_F \neq \beta_M$, with values denoted by F_CE and M_CE for females and males, respectively. MSEs were computed over 1000 simulation replicates. Intervals around the estimated MSE correspond to the MSE +/- one estimated standard error.

Abbreviations: MSE: mean square error; MR: Mendelian randomization; APM: adaptive posterior mean; AW: adaptive weight; diff: different level of sex differences in variant-exposure effects; Cor: correlation between female and male variant-exposure effect; CE: causal effect; W-median: weighted median; IVW*: penalized and robust IVW; Egger*: penalized and robust MR-Egger; ConMix: contaminated mixture; cML: constrained maximum likelihood; RAPS: MR-RAPS.

Figure S2: 95% CI coverage rates of the true causal effect from primary simulation studies

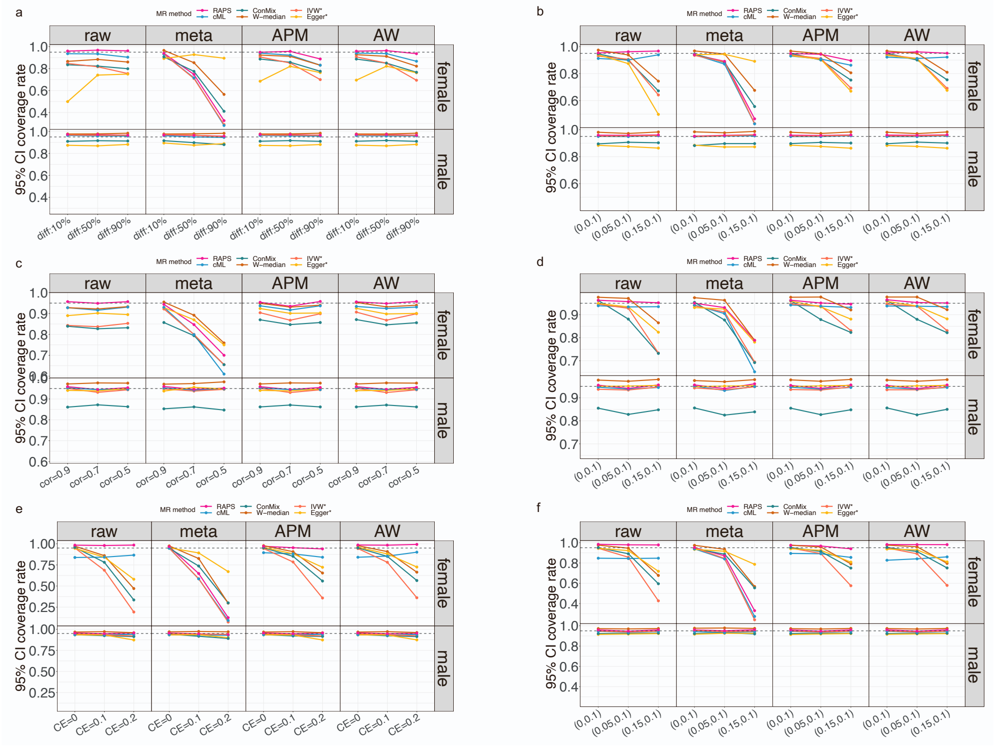

Coverage rates for the simulated true β_F and β_M values are shown, using 95% CI computed by each MR method across 1000 simulation replicates. In each result grid, female and male results are indicated by row labels, and methods used to estimate the variant-exposure effect sizes are indicated by column labels. Two-sample MR methods used are indicated by colors as labeled. Panels a and b provide results from simulations setting with fixed sex differences in γ, panels c and d correspond to settings with random sex differences in γ, and panels e and f correspond to settings with MVP OSA GWAS summary guided γ effect sizes. Left panels (a, c, e) corresponds to settings with $\beta_F = \beta_M$ (CE), while the right panels (b, d, f) correspond to settings with $\beta_F \neq \beta_M$. In panel a and c, we set $\beta_F = \beta_M = 0.1$. In panel e, the underlying causal effect is shown at the bottom of the figure. Results from tests of sex differences in causal effect are provided in the right panels (b, d, f), with the underlying sex-specific causal effect shown in parentheses below (x-axis labels) in the form (β_F, β_M).

Abbreviations: CI: confidence interval; MR: Mendelian randomization; APM: adaptive posterior mean; AW: adaptive weight; diff: different level of sex differences in exposure effects; Cor: correlation between female and male exposure effect; CE: causal effect; W-median: weighted median; IVW*: penalized and robust IVW; Egger*: penalized and robust MR-Egger; ConMix: contaminated mixture; cML: constrained maximum likelihood; RAPS: MR-RAPS.

Summary of female-specific exposure-outcome causal effect estimation

We used boxplot to display the distribution of $\hat{\beta}_F$ using either shrinkage estimates ($\hat{\gamma}_{meta}, \hat{\gamma}_{APM}$, $\hat{\gamma}_{AW}$) or $\hat{\gamma}_{raw}$ as input for MR analysis. The results are presented in Figure S3. The boxplot illustrates $\hat{\beta}_F$ across 1000 simulation replicates, with the horizontal dashed red line indicating the underlying β_F value.

In the simulation with fixed strength sex differences in γ (Figure S3, panel a and b), most MR methods achieved unbiased estimation of β_F, except the MR-Egger approach. The shrinkage estimates resulted in smaller variance in causal effect estimates than the use of $\hat{\gamma}_{raw}$. In the settings where 90% of the variants exhibit sex differences in γ, only the cML and MR-RAPS methods resulted in unbiased estimates using $\hat{\gamma}_{raw}$. In contrast, while cML and MR-RAPS resulted in slightly lower average causal effect estimates when using $\hat{\gamma}_{APM}$, they achieved smaller variance in $\hat{\beta}_F$ compared to using $\hat{\gamma}_{raw}$. Therefore, the overall MSE of the causal effect estimate was smaller than that of using $\hat{\gamma}_{raw}$. (Figure 2a in the main manuscript).

In simulations with random strengths of sex differences in γ (Figure S3, panel c and d), the $\hat{\gamma}_{meta}$ approach produced biased estimates when the correlation between γ_F and γ_M was low or moderate (correlation = 0.5 and 0.7). However, the $\hat{\gamma}_{APM}$ and $\hat{\gamma}_{AW}$ approaches still produced unbiased or nearly unbiased estimates in these settings. The $\hat{\gamma}_{meta}$ approach produced unbiased estimates for all MR methods in high-correlation setting. The improvement from using the $\hat{\gamma}_{APM}$ approach was more significant in the weighted median and IVW these two methods, which produced estimates closer to the underlying true causal effect on average compared to

using $\hat{\gamma}_{raw}$. The cML and MR-RAPS methods produced unbiased estimates in all settings, regardless of whether the shrinkage or raw estimate was used.

Of the simulation using MVP OSA GWAS to guide γ effect size (Figure S3, panel e and f), all methods provided unbiased estimates in the null causal effect setting ($\beta_F = 0$), regardless of β_M. As the true causal effect increased, the bias also increased with the $\hat{\gamma}_{meta}$ approach, but this had less influence on the methods using the $\hat{\gamma}_{APM}$ or $\hat{\gamma}_{AW}$ approaches. The $\hat{\gamma}_{APM}$ approach significantly reduced the variance of causal effect estimates compared to the use of $\hat{\gamma}_{raw}$, especially in the cML and MR-RAPS methods, which typically showed smaller MSE in all settings.

Figure S3: Boxplots of female-specific exposure-outcome causal effect estimates from primary simulation studies

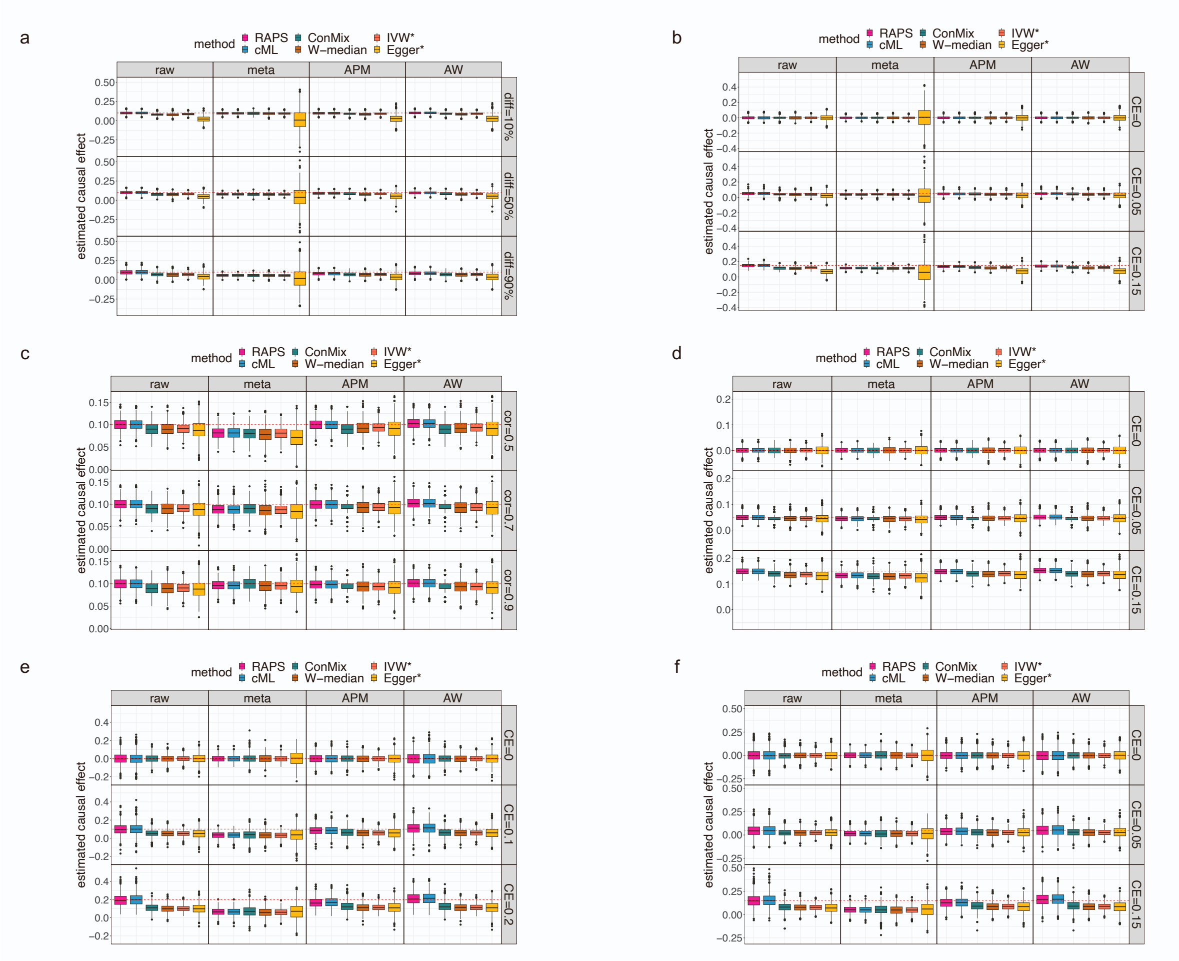

This figure shows the female-specific exposure-outcome causal effect estimates using boxplots from each MR method. Each boxplot represents β_F estimates across 1,000 simulation replicates. We evaluated six two-sample MR methods for estimating the causal effect: W-median, IVW*, Egger*, ConMix, cML, and RAPS. In each panel, columns are labeled according to the type of variant-exposure effect estimate used. Panels a and b illustrate results from simulation settings with fixed sex differences in γ. Panels c and d show results from settings with random sex differences in γ. Panels e and f display the results from settings using MVP OSA GWAS summary statistics to guide sex-specific γ effect sizes. The left panels (a, c, e) present the results of no sex differences in causal effect settings ($\beta_F = \beta_M$), while the right panels (b, d, f) show results from settings with $\beta_F \neq \beta_M$. In the latter settings, $\beta_M = 0.1$, and simulated β_F values are indicated in the legend on the right-hand side of each panel (CE). The horizontal dashed redline highlight the simulated, true, β_F.

Abbreviations: MR: Mendelian randomization; APM: adaptive posterior mean; AW: adaptive weight; diff: different level of sex differences in variant-exposure effects; Cor: correlation between female and male variant-exposure effects; CE: causal effect; W-median: weighted median; IVW*: penalized and robust IVW; Egger*: penalized and robust MR-Egger; ConMix: contaminated mixture; cML: constrained maximum likelihood; RAPS: MR-RAPS.

Note S2: Secondary simulation studies

We consider additional simulation studies to examine the usefulness of the proposed shrinkage estimate framework in (i) improving the estimation of γ and (ii) comparing the performance of β estimation using both shrinkage and raw estimates as input in MR analysis. We first investigated whether the shrinkage estimate achieves better estimation performance for γ, mainly focusing on populations with smaller sample sizes (female population). Next, we use the same settings as in simulation 1 (fixed sex differences strength in γ) to conduct two additional analyses. Specifically, we first examined the situation where some variants directly affect the outcome (i.e., a horizontal pleiotropy effect), which results in some variants being invalid IVs for the analysis. Second, we compared the performance of β estimation between two strategies: (i) applying the APM shrinkage approach only to γ or (ii) applying it to both γ and Γ estimates.

We performed simulations with increasing sample sizes for the female population so that balanced sample sizes between sex groups in the exposure GWAS was also considered. This analysis aims to evaluate the performance of the shrinkage approaches in a setting where less information can be transferred between sex groups. Finally, we conducted a test of differences in the sex-specific causal effects obtained from the two-sample MR approaches (i.e., $H_0: \beta_F = \beta_M$), assessing the type 1 error rate and power of each MR method.

Secondary simulation study 1: Examining variant-exposure effect estimation performance

Given that the accuracy of the $\hat{\gamma}$ might highly impacts the precision of the β estimation, especially in the presence of weak IVs in the population with smaller sample sizes, we first

evaluated the estimation performance of $\hat{\gamma}$ when using shrinkage approaches ($\hat{\gamma}_{meta}, \hat{\gamma}_{APM}, \hat{\gamma}_{AW}$) and $\hat{\gamma}_{raw}$. Our rationale was that if shrinkage estimates demonstrate better performance, then more accurate and stable $\hat{\beta}$ using these shrinkage estimates can be anticipated. The results are summarized in Figures S4, S5, and S6. When fewer variants have sex differences in γ or when these differences are minor, the $\hat{\gamma}_{meta}$ estimate has lower MSE compared to other estimates. The $\hat{\gamma}_{APM}$ and $\hat{\gamma}_{AW}$ estimates consistently outperform the $\hat{\gamma}_{raw}$ across all simulations. When more variants having sex differences in γ ($|D_{\gamma}| = 50$ and $|D_{\gamma}| = 90$ settings) or the correlation between γ_F and γ_M is not strong (correlation = 0.5 setting), $\hat{\gamma}_{APM}$ and $\hat{\gamma}_{AW}$ perform better than $\hat{\gamma}_{meta}$. All methods performed similarly in the male population, with the $\hat{\gamma}_{meta}$ showing slightly higher MSE than the others.

Figure S4: Boxplots for MSE of variant-exposure effect estimates in primary simulation studies 1

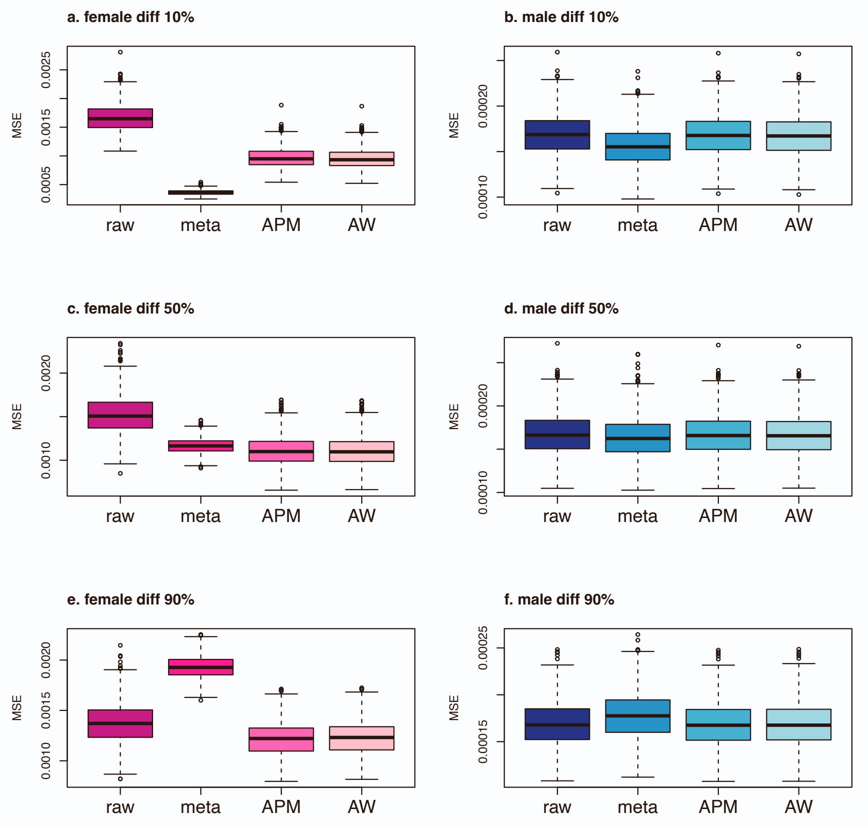

The figure shows the estimation performance of the variant-exposure (γ) effect estimate using shrinkage approaches (meta, APM, and AW) and the uncalibrated (raw) estimates from secondary simulation studies 1. For each simulation replicate, MSE values were computed averaging all 100 variants, while the boxplots use the resulting MSEs across the 1000 simulation replicates. Results of the female-specific and male-specific variant-exposure estimate are shown on the left and right panels, respectively.

Abbreviations: MSE: mean square error; APM: adaptive posterior mean; AW: adaptive weight; diff: different level of sex differences in variant-exposure effects.

Figure S5: Boxplots of MSE of variant-exposure effect estimates in primary simulation study 2

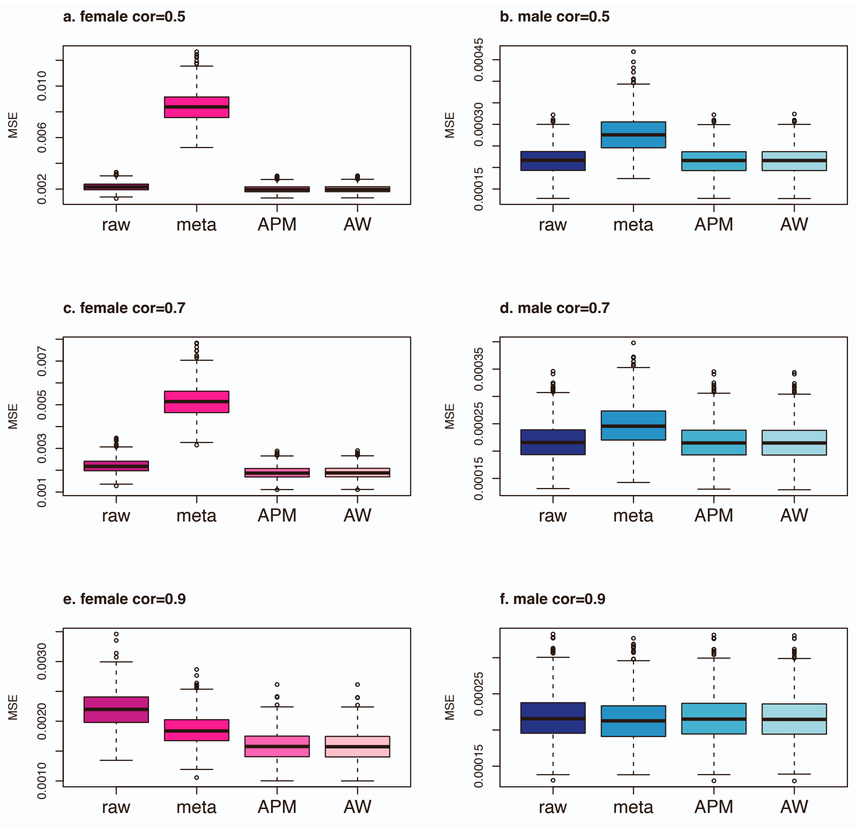

This figure shows the estimation performance of the variant-exposure effects using shrinkage approaches (meta, APM, and AW) and the uncalibrated (raw) estimates in secondary simulation studies 2. The correlation between γ_F and γ_M are shown in the subtitle of each panel (e.g., cor = 0.5, 0.7, 0.9). For each simulation replicate, MSE values were computed averaging all 100 variants, while the boxplots use the resulting MSEs across the 1000 simulation replicates. Results of the female-specific and male-specific variant-exposure estimate are shown on the left and right panels, respectively.

Abbreviations: MSE: mean square error; APM: adaptive posterior mean; AW: adaptive weight; cor: correlation between γ_F and γ_M.

Figure S6: Boxplot for MSE of variant-exposure effect estimates in primary simulation study 3

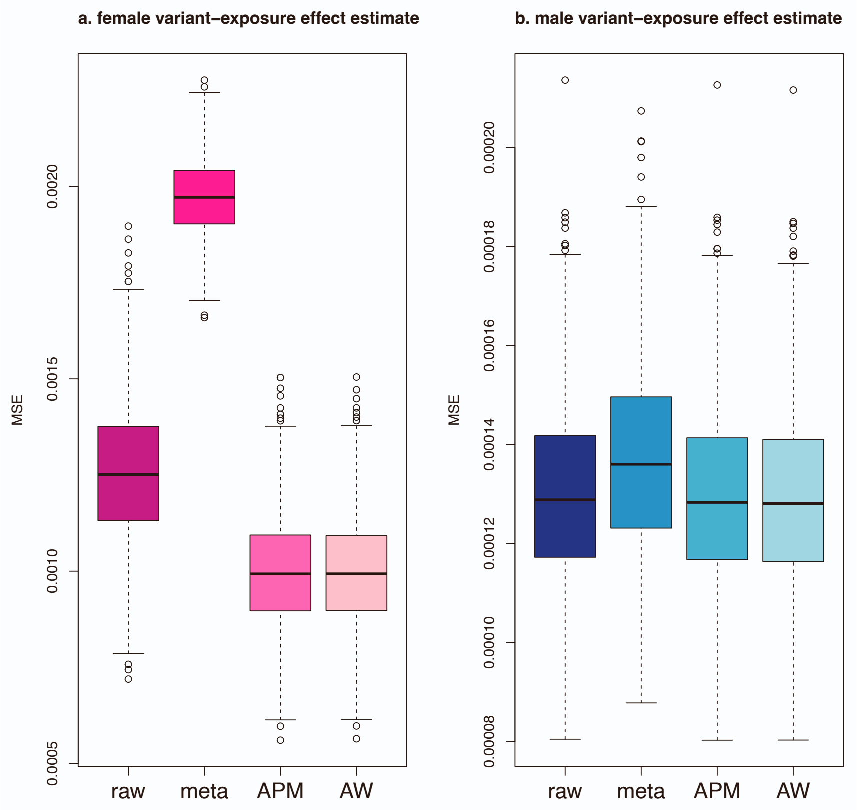

MSE of variant-exposure effect estimate using shrinkage approaches (meta, APM, and AW) and the uncalibrated (raw) estimates from secondary simulations 3. For each simulation replicate, MSE values were computed averaging all 100 variants, while the boxplots use the resulting MSEs across the 1000 simulation replicates. The results of the female-specific variant-exposure estimate are shown on the left panel, and the male-specific variant-exposure estimate results are shown on the right panel.

Abbreviations: MSE: mean square error; APM: adaptive posterior mean; AW: adaptive weight.

Secondary simulation study 2: Some IVs are associated with the outcome (pleiotropy)
We consider the settings with fixed sex differences in γ, while randomly selecting some variants as being pleiotropic. Specifically, we randomly selected 20 variants to have a direct effect (not mediated by the exposure) on the outcome. We generated the outcome variable by the following model:

$$O_i = 1 + \sum_{j \in K} \lambda_j \times g_{ij} + \beta \times E_i + \alpha \times U_i + \varepsilon_i$$

where K denotes the set of pleiotropic variants, and λ_j represents their direct effect sizes on the outcome (not mediated via the exposure).

Balanced pleiotropy simulations. First, the direct effects λ_j were independently generated from a normal distribution $\lambda_j \sim N(0,0.1)$, corresponding to a balanced pleiotropy assumption. We used the same procedure described in the Method section in the main manuscript to generate genetic variants (g_{ij}), exposure (E_i), unknown confounder (U_i), and random error term (ε_i). The variants with pleiotropic effects were the same in the two sex groups, and the direct effects were generated from the normal distribution. Both settings with and without sex differences in the exposure-outcome causal effect were considered. The female and male exposure GWAS sample sizes were 2,000 and 20,000, respectively. Results were summarized across 1000 simulation replicates.

Simulation results are displayed in Figures S7 and S8. The conclusions align with the findings from the primary simulation studies. Overall, the shrinkage approaches have smaller MSE than estimates using $\hat{\gamma}_{raw}$. Estimates using $\hat{\gamma}_{meta}$ usually have the smallest MSE when the sex

differences in γ are not weak. Both the APM and AW methods have more robust performance in terms of MSE and 95% confidence interval coverage rate, regardless of the magnitude of sex differences in γ. When having sex differences in causal effect (Figures S7 b and S8 b), using $\hat{\gamma}_{APM}$ for MR analysis significantly improves causal effect estimation performance and the 95% confidence interval coverage rate when the female population has a higher underlying causal effect. When comparing robust methods to traditional versions (robust IVW vs. IVW and robust MR-Egger vs. MR-Egger), the robust versions have much lower MSEs, demonstrating the advantages of using robust approaches when invalid IVs are present. For the male population, with less information transferable from females, the shrinkage approaches perform similarly to the use of $\hat{\gamma}_{raw}$.

Directional pleiotropy simulations. Here, the direct effects λ_j were independently generated from a normal distribution $\lambda_j \sim N(0.1,0.1)$, i.e., with a non-zero mean. The results are presented in Figures S9 and S10. Under this simulation setting, all methods performed worse than in the balanced pleiotropy setting, with IVW showing the greatest decline in performance. For MR-RAPS, the violation of model assumptions under directional pleiotropy led to suboptimal performance compared to other methods, although the differences were relatively small. The most notable impact of the violation of directional pleiotropy effect on MR-RAPS was observed in the 95% confidence interval coverage, which was significantly reduced in this analysis. However, incorporating $\hat{\gamma}_{APM}$ into MR-RAPS significantly improved the performance compared to the use of $\hat{\gamma}_{raw}$, with lower MSE and higher 95% confidence interval coverage rate

across all simulation settings. Overall, in terms of MSE, robust IVW has the smallest MSE among all MR methods, followed by MR-RAPS and cML.

Figure S7: MSE of sex-specific causal effect estimates from simulations with balanced pleiotropic variants

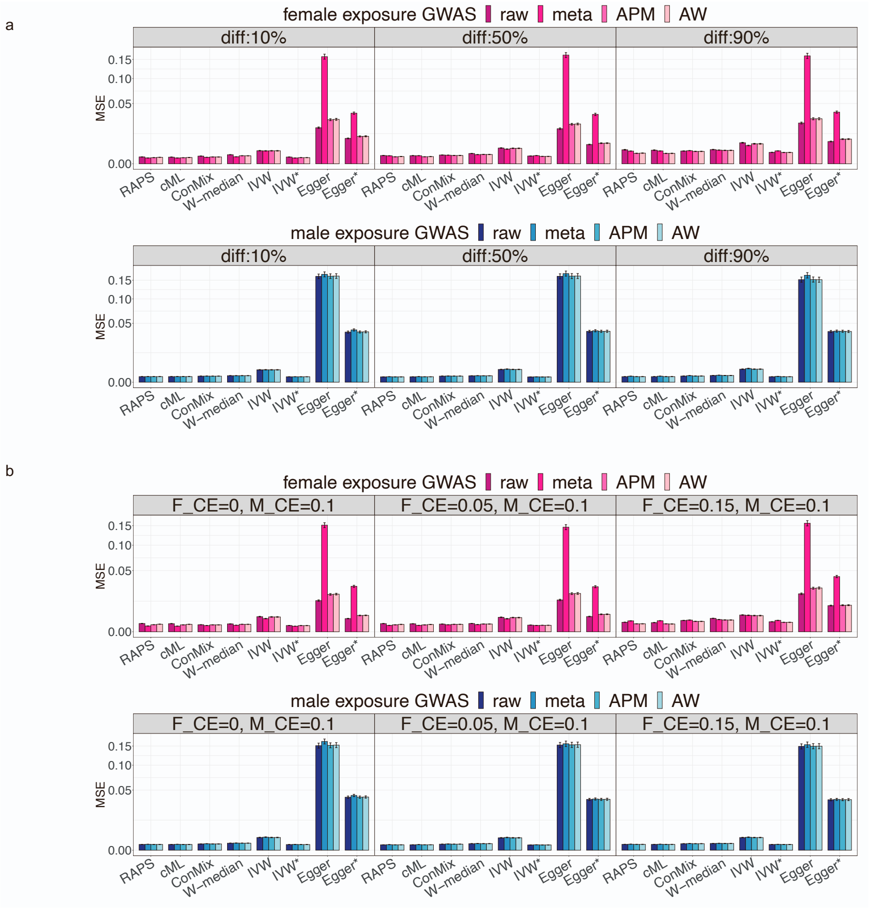

This figure shows the MSE of sex-specific causal effect estimates. Female results are in color pink, and male results are in color blue. We considered eight two-sample MR methods to estimate the causal effect: W-median, IVW, IVW*, MR-Egger, Egger*, ConMix, cML, and RAPS. The color level of the bars corresponds to type of variant-exposure estimate used. The results from simulation settings with no sex differences in causal effect ($\beta_F = \beta_M = 0.1$) are shown in panel a, and MSEs from settings with sex differences in causal effect are shown in panel b. in panel b, the β_F and β_M values are labeled as F_CE and M_CE, representing the causal effects for females and males, respectively. MSEs were computed over 1000 simulation replicates. Intervals around the estimated MSE correspond to the MSE +/- one estimated standard error.

Abbreviations: MSE: mean square error; MR: Mendelian randomization; APM: adaptive posterior mean; AW: adaptive weight; diff: different level of sex differences in exposure effects; CE: causal effect; W-median: weighted median; IVW: inverse-variance weighted; IVW*: penalized and robust IVW; Egger: MR-Egger; Egger*: penalized and robust MR-Egger; ConMix: contaminated mixture; cML: constrained maximum likelihood; RAPS: MR-RAPS.

Figure S8: Confidence interval coverage rate of the true causal effect from balanced pleiotropy effect simulation studies

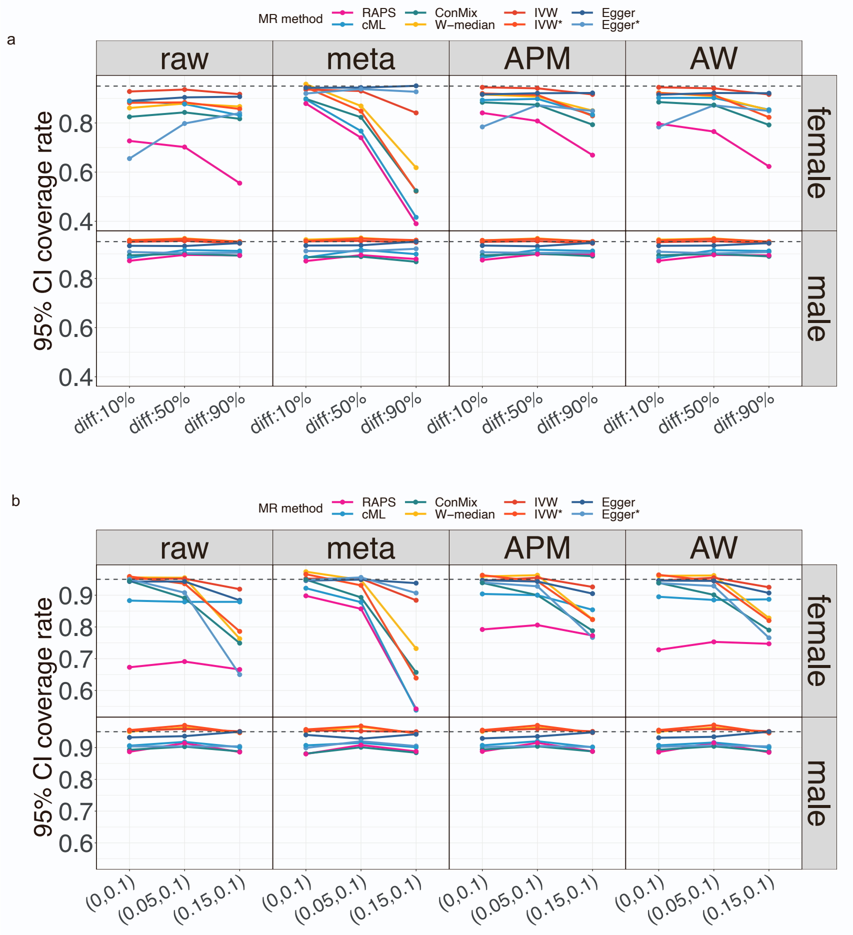

Coverage rates of the underlying true sex-specific causal effect using 95% CI computed by each MR method across 1000 simulation replicates. We considered eight two-sample MR methods to estimate the causal effect: W-median, IVW, IVW*, MR-Egger, Egger*, ConMix, cML, and RAPS. Column titles describe which variant-exposure effect estimate approach was used. Results from simulation settings with $\beta_F = \beta_M = 0.1$ are illustrated in panel a. Panel b provides results from simulations with $\beta_F \neq \beta_M$, with the underlying sex-specific causal effect shown in the parentheses below (x-axis) in the form (β_F, β_M).

Abbreviations: CI: confidence interval; MR: Mendelian randomization; APM: adaptive posterior mean; AW: adaptive weight; diff: different level of sex differences in exposure effects; W-median: weighted median; IVW: inverse-variance weighted; IVW*: penalized and robust IVW; Egger: MR-Egger; Egger*: penalized and robust MR-Egger; ConMix: contaminated mixture; cML: constrained maximum likelihood; RAPS: MR-RAPS.

Figure S9: MSE of sex-specific causal effect estimates from simulations with directional pleiotropic variants

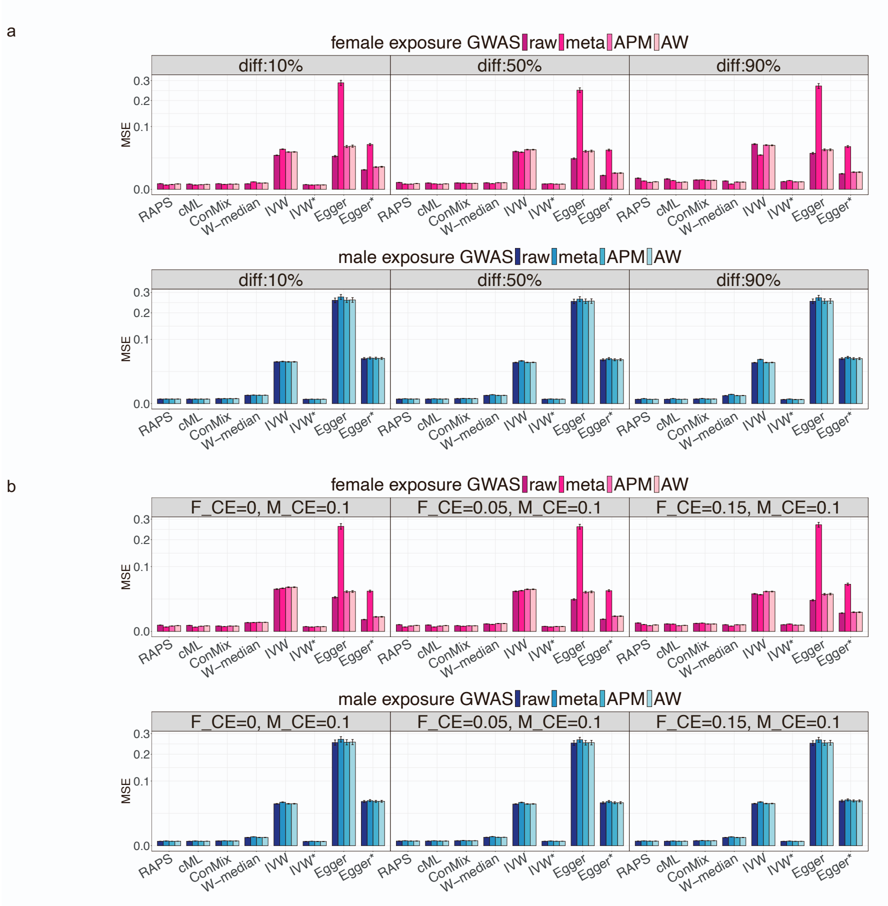

This figure shows the MSE of sex-specific causal effect estimates. Female results are in color pink, and male results are in color blue. We considered eight two-sample MR methods to estimate the causal effect: W-median, IVW, IVW*, MR-Egger, Egger*, ConMix, cML, and RAPS. The color level of the bars corresponds to type of variant-exposure estimate used. The results from simulation settings with no sex differences in causal effect ($\beta_F = \beta_M = 0.1$) are shown in panel a, and MSEs from settings with sex differences in causal effect are shown in panel b. in panel b, the β_F and β_M values are labeled as F_CE and M_CE, representing the causal effects for females and males, respectively. MSEs were computed over 1000 simulation replicates. Intervals around the estimated MSE correspond to the MSE +/- one estimated standard error.

Abbreviations: MSE: mean square error; MR: Mendelian randomization; APM: adaptive posterior mean; AW: adaptive weight; diff: different level of sex differences in exposure effects; CE: causal effect; W-median: weighted median; IVW: inverse-variance weighted; IVW*: penalized and robust IVW; Egger: MR-Egger; Egger*: penalized and robust MR-Egger; ConMix: contaminated mixture; cML: constrained maximum likelihood; RAPS: MR-RAPS.

Figure S10: Confidence interval coverage rate of the true causal effect from directional pleiotropy effect simulation studies

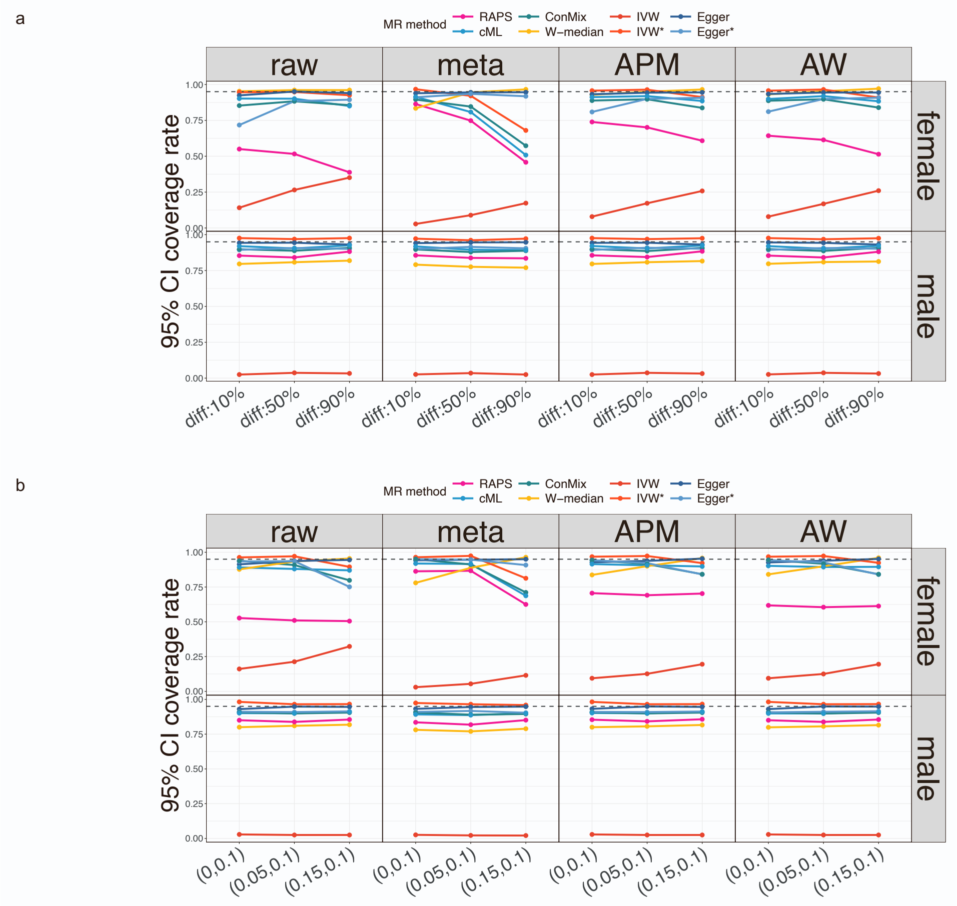

Coverage rates of the underlying true sex-specific causal effect using 95% CI computed by each MR method across 1000 simulation replicates. We considered eight two-sample MR methods to estimate the causal effect: W-median, IVW, IVW*, MR-Egger, Egger*, ConMix, cML, and RAPS. Column titles describe which variant-exposure effect estimate approach was used. Results from simulation settings with $\beta_F = \beta_M = 0.1$ are illustrated in panel a. Panel b provides results from simulations with $\beta_F \neq \beta_M$, with the underlying sex-specific causal effect shown in the parentheses below (x-axis) in the form (β_F, β_M).

Abbreviations: CI: confidence interval; MR: Mendelian randomization; APM: adaptive posterior mean; AW: adaptive weight; diff: different level of sex differences in exposure effects; W-median: weighted median; IVW: inverse-variance weighted; IVW*: penalized and robust IVW; Egger: MR-Egger; Egger*: penalized and robust MR-Egger; ConMix: contaminated mixture; cML: constrained maximum likelihood; RAPS: MR-RAPS.

Secondary simulation study 3: Calibrating both variant-exposure and variant-outcome effect estimates

In the primary simulation studies, we only applied the proposed semi-empirical Bayes framework for calibrating $\hat{\gamma}$. There are two main reasons for this: (i) the motivating example, the MVP sleep phenotype, serves as the exposure variable in our real data MR analysis; (ii) unstable $\hat{\gamma}$ have more severe impact on causal effect estimation than variant-outcome effect estimates ($\hat{\Gamma}$). However, the proposed framework is generalizable and can be applied to any trait's role in an MR analysis. Therefore, we examined the performance of calibrating both $\hat{\gamma}$ and $\hat{\Gamma}$ in a two-sample MR analysis. We used the fixed sex differences in γ simulation settings to examine the results. To simplify the comparison, we focus on the raw and APM approached in variant-exposure and variant-outcome effect estimate, which already form four different combinations.

Figures S11 and S12 summarize the results of this simulation study. For the settings with no sex differences in β (Figures S11 a and S12 a), calibrating both $\hat{\gamma}$ and $\hat{\Gamma}$ performs the best (smaller MSE) for all MR methods except the MR-Egger approach. A slight improvement in causal effect estimation performance was also observed in the male population. However, calibrating both estimates did not improve the causal effect estimation in some of the sex difference causal effect settings (Figures S11 b and S12 b). Specifically, calibrating $\hat{\Gamma}$ may result in higher MSE for causal effect estimation in both female and male populations in the null female-specific causal effect setting ($\beta_F = 0, \beta_M \neq 0$). In this setting, the 95% coverage rate is much lower than the raw or only calibrating for $\hat{\gamma}$ in both sex groups. One possible reason could be that the shrinkage estimates significantly reduce the standard error estimate for both $\hat{\gamma}$ and $\hat{\Gamma}$, which may lead to an underestimation of the variance of causal effect estimate. In brief, although the

proposed framework can generally be applied to calibrate any variant-phenotype association effect estimate in MR analysis, simulation results suggest its practical use is particularly evident in calibrating $\hat{\gamma}$ to address weak instrument bias issue.

Figure S11: MSE of sex-specific causal effect estimates from simulations calibrating both variant-exposure and outcome analyses

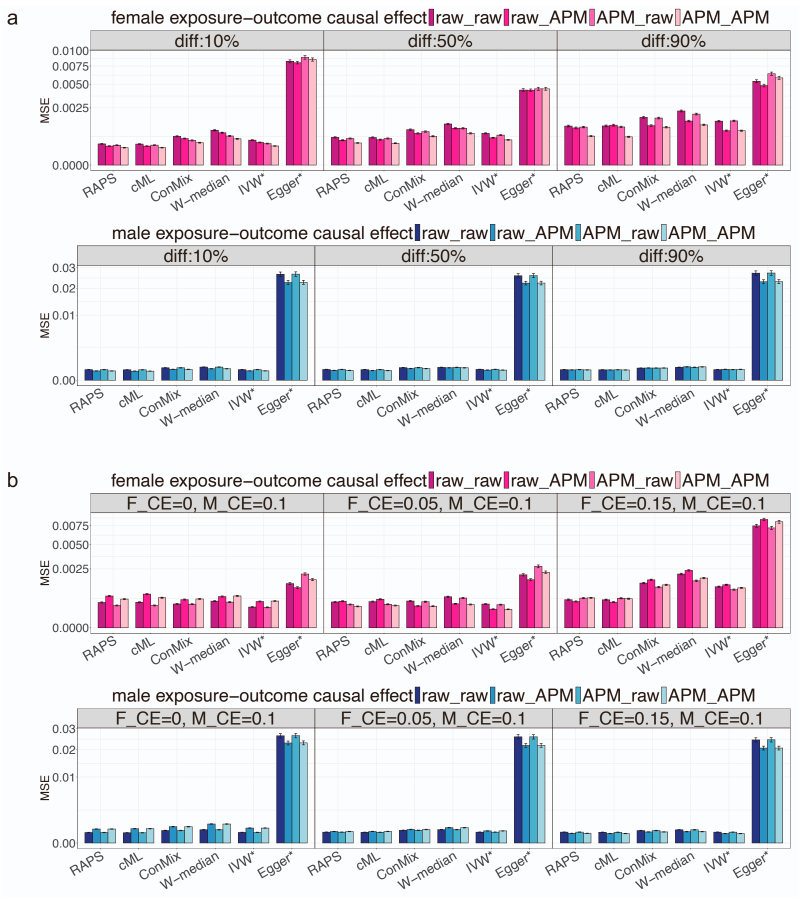

This figure shows the MSE of sex-specific causal effect estimates in secondary simulation study 3. The fixed sex differences in γ setting is applied in this analysis. Female results are in color pink, and male results are in color blue. We considered six two-sample MR methods to estimate the causal effect: W-median, IVW*, Egger*, ConMix, cML, and RAPS. Four combinations of variant-phenotype effect estimates are considered: no calibration for both variant-exposure and variant-outcome effect estimate (raw_raw), calibration only for variant-outcome effect estimate using APM (raw_APM), calibration only for variant-exposure effect estimate using APM (APM_raw), and calibration for both variant-exposure and variant-outcome effect estimate (APM_APM). The results of no sex differences in causal effect settings ($\beta_F = \beta_M = 0.1$) are shown in panel a, and MSEs of sex differences in causal effect settings are shown in panel b, with β_F and β_M values denoted by F_CE and M_CE for females and males, respectively. MSEs were computed over 1000 simulation replicates. Intervals around the estimated MSE correspond to the MSE +/- one estimated standard error.

Abbreviations: MSE: mean square error; MR: Mendelian randomization; APM: adaptive posterior mean; AW: adaptive weight; diff: different level of sex differences in exposure effects; CE: causal effect; W-median: weighted median; IVW: inverse-variance weighted; IVW*: penalized and robust IVW; Egger: MR-Egger; Egger*: penalized and robust MR-Egger; ConMix: contaminated mixture; cML: constrained maximum likelihood; RAPS: MR-RAPS.

Figure S12: Confidence interval coverage rate of the true causal effect in simulations calibrating both variant-exposure and outcome analyses

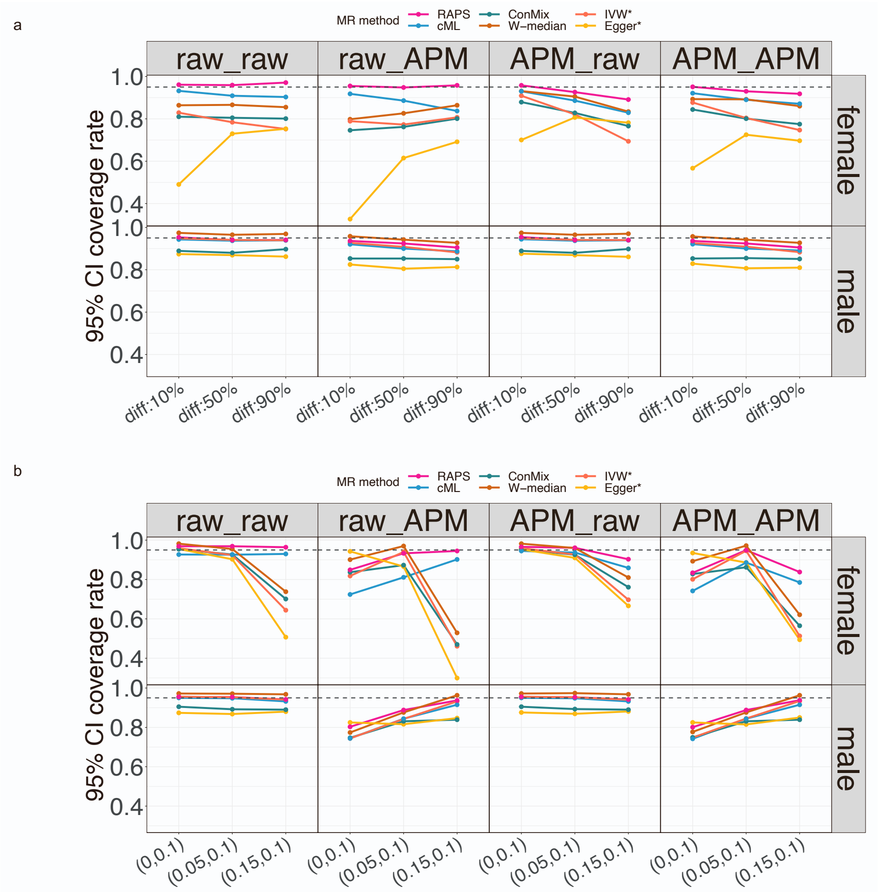

The figure provides coverage rates of the underlying true sex-specific causal effect using 95% CI computed by each MR method across 1000 simulation replicates. Female results are shown at the top, and male results are shown at the bottom. We considered six two-sample MR methods to estimate causal effect: W-median, IVW*, Egger*, ConMix, cML, and RAPS. Four combinations of variant-phenotype effect estimates are considered: no calibration for both variant-exposure and variant-outcome effect estimate (raw_raw), calibration only for variant-outcome effect estimate using APM (raw_APM), calibration only for variant-exposure effect estimate using APM (APM_raw), and calibration for both variant-exposure and variant-outcome effect estimate (APM_APM). The results of the same causal effect settings are illustrated in panel a, in which the causal effects are set as 0.1 for both females and males. Panel b provides results from simulations with $\beta_F \neq \beta_M$, with the underlying sex-specific causal effect shown in parentheses below (x-axis) in the form (β_F, β_M).

Abbreviations: CI: confidence interval; MR: Mendelian randomization; APM: adaptive posterior mean; AW: adaptive weight; diff: different level of sex differences in exposure effects; W-median: weighted median; IVW: inverse-variance weighted; IVW*: penalized and robust IVW; Egger: MR-Egger; Egger*: penalized and robust MR-Egger; ConMix: contaminated mixture; cML: constrained maximum likelihood; RAPS: MR-RAPS.

Secondary simulation study 4: Balanced sex groups sample sizes in exposure GWAS

In this simulation study, we perform all three simulation settings in primary analysis again but increase the sample size of the female exposure GWAS population from 2,000 to 20,000, matching the male population sample size. Therefore, in these simulations, in all calibrated variant-exposure estimates we expect the weight of the female-specific estimates to increase relative to the male-specific weight, and, consequently, we expect less improvement in β_F estimation from shrinkage approaches compared to the raw approach.

The results from this simulation study are summarized in Figures S13 and S14. Considering MSE, estimation of β_F while relying on γ_{APM} performs nearly the same as the use of $\hat{\gamma}_{raw}$ in both female and male analyses. When strong sex differences in γ exist, particularly in simulation 3, $\hat{\gamma}_{meta}$ had the highest MSE

Figure S13: MSE of sex-specific causal effect estimates from secondary simulation 4 (equivalent female-male sample size)

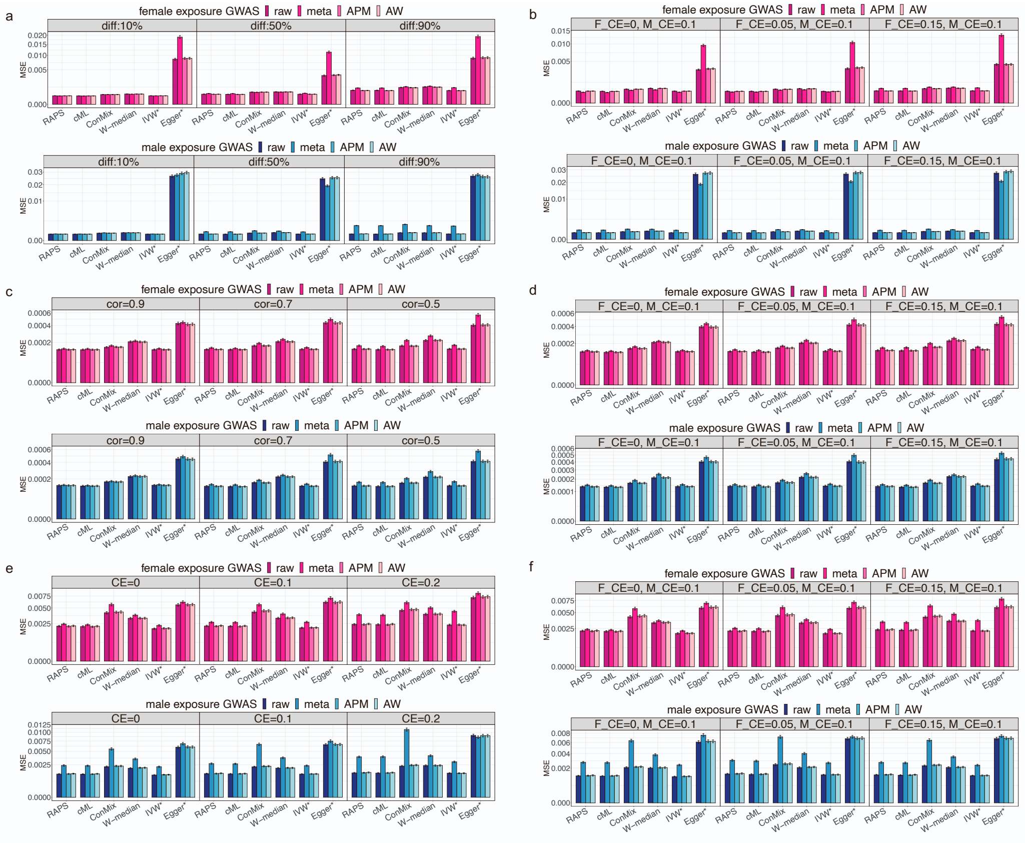

This figure shows the MSE of estimated β_F and β_M in secondary simulation 4. Female results are in pink, and male results are in blue. Two-sample MR methods are annotated as x-axis labels. Shade of bars correspond to the type of variant-exposure effect estimate (raw, meta, APM, AW), as labeled. Panels a and b provide results from simulation settings with fixed sex differences in γ, panels c and d correspond to settings with random sex differences in γ, and panels e and f correspond to settings with MVP OSA GWAS-guided sex-specific γ effect sizes. Left panels (a, c, e) corresponds to settings with $\beta_F = \beta_M$ (CE), while the right panels (b, d, f) correspond to settings with $\beta_F \neq \beta_M$, with values denoted by F_CE and M_CE for females and males, respectively. MSEs were computed over 1000 simulation replicates. Intervals around the estimated MSE correspond to the MSE +/- one estimated standard error.

Abbreviations: MSE: mean square error; MR: Mendelian randomization; APM: adaptive posterior mean; AW: adaptive weight; diff: different level of sex differences in variant-exposure effects; Cor: correlation between female and male variant-exposure effect; CE: causal effect; W-median: weighted median; IVW*: penalized and robust IVW; Egger*: penalized and robust MR-Egger; ConMix: contaminated mixture; cML: constrained maximum likelihood; RAPS: MR-RAPS.

Figure S14: Confidence interval coverage rate of the true causal effect from secondary simulation 4 (equivalent female-male sample size)

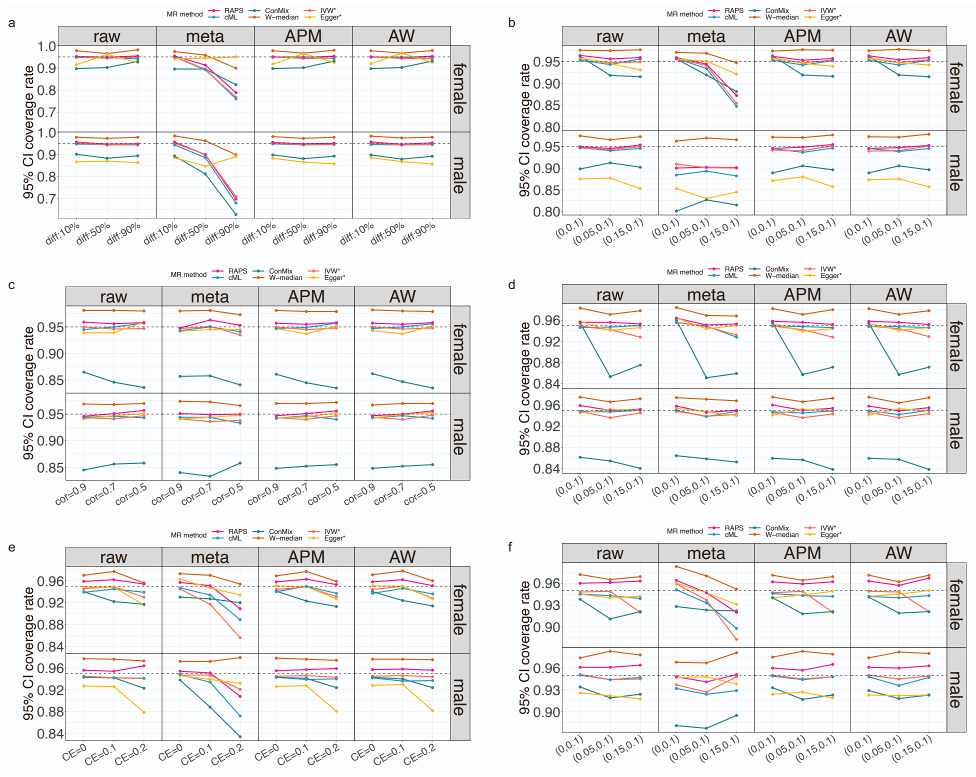

Coverage rates for the simulated true β_F and β_M values are shown, using 95% CI computed by each MR method across 1000 simulation replicates. In each result grid, female and male results are indicated by row labels, and methods used to estimate the variant-exposure effect sizes are indicated by column labels. Two-sample MR methods used are indicated by colors as labeled. Panels a and b provide results from simulations setting with fixed sex differences in γ, panels c and d correspond to settings with random sex differences in γ, and panels e and f correspond to settings with MVP OSA GWAS summary guided γ effect sizes. Left panels (a, c, e) corresponds to settings with $\beta_F = \beta_M$ (CE), while the right panels (b, d, f) correspond to settings with $\beta_F \neq \beta_M$. In panel a and c, we set $\beta_F = \beta_M = 0.1$. In panel e, the underlying causal effect is shown at the bottom of the figure. Results from tests of sex differences in causal effect are provided in the right panels (b, d, f), with the underlying sex-specific causal effect shown in parentheses below (x-axis labels) in the form (β_F, β_M).

Abbreviations: CI: confidence interval; MR: Mendelian randomization; APM: adaptive posterior mean; AW: adaptive weight; diff: different level of sex differences in exposure effects; Cor: correlation between female and male exposure effect; CE: causal effect; W-median: weighted median; IVW*: penalized and robust IVW; Egger*: penalized and robust MR-Egger; ConMix: contaminated mixture; cML: constrained maximum likelihood; RAPS: MR-RAPS.

Secondary simulation study 5: Sex-differences test

We evaluated the type 1 error and power of the test for sex-differences in causal effect across considered MR methods. The following test statistics is used

$$t_{\beta_F,\beta_M} = \frac{\hat{\beta}_F - \hat{\beta}_M}{\sqrt{\hat{\sigma}^2_{\beta_F} + \hat{\sigma}^2_{\beta_M}}} \qquad \text{(Equation 1)}$$

where $\hat{\beta}_F$ and $\hat{\beta}_M$ are the sex-specific causal estimates from a given MR method, and $\hat{\sigma}_{\beta_F}$, $\hat{\sigma}_{\beta_M}$ are their corresponding estimated standard errors. The test statistic in Equation 1 assumes that $\hat{\beta}_F$ and $\hat{\beta}_M$ are independent, so we only include the uncalibrated approach ($\hat{\gamma}_{raw}$) in this analysis. The p-value is computed through $\chi^2_{(1)}$ distribution, and the rejection rate is calculated as the proportion of p-value < 0.05 across 1000 simulation replicates. In settings with no sex differences in causal effects ($\beta_F = \beta_M$), the rejection rate is the type I error rate; while the rejection rate estimates power when $\beta_F \neq \beta_M$.

Simulation results are shown in Figure S15. Overall, the penalized and robust IVW (IVW* in the figure) has an inflated type I error rate, so a higher power of detecting sex differences is expected. In contrast, the weighted median and MR-RAPS are conservative, having type I error rates lower than 0.05. Still, MR-RAPS often has comparable or even higher power, compared to other methods, in our simulations.

Figure S16 presents results evaluating the sex-differences test in the secondary simulation studies 4 (equivalent female-male sample size, and overall higher sample size). Overall, most of

the MR methods controlled the type I error rate around the nominal threshold for the sex-differences test. The robust and penalized IVW and cML yielded a slightly inflated type I error rate, while MR-RAPs usually produced more conservative results. In terms of power, the robust and penalized IVW, cML, and MR-RAPs have higher power to detect sex differences in the causal effect compared to weighted median and penalized and robust MR-Egger.

Figure S15: Rejection rates of sex-differences test from primary simulation studies

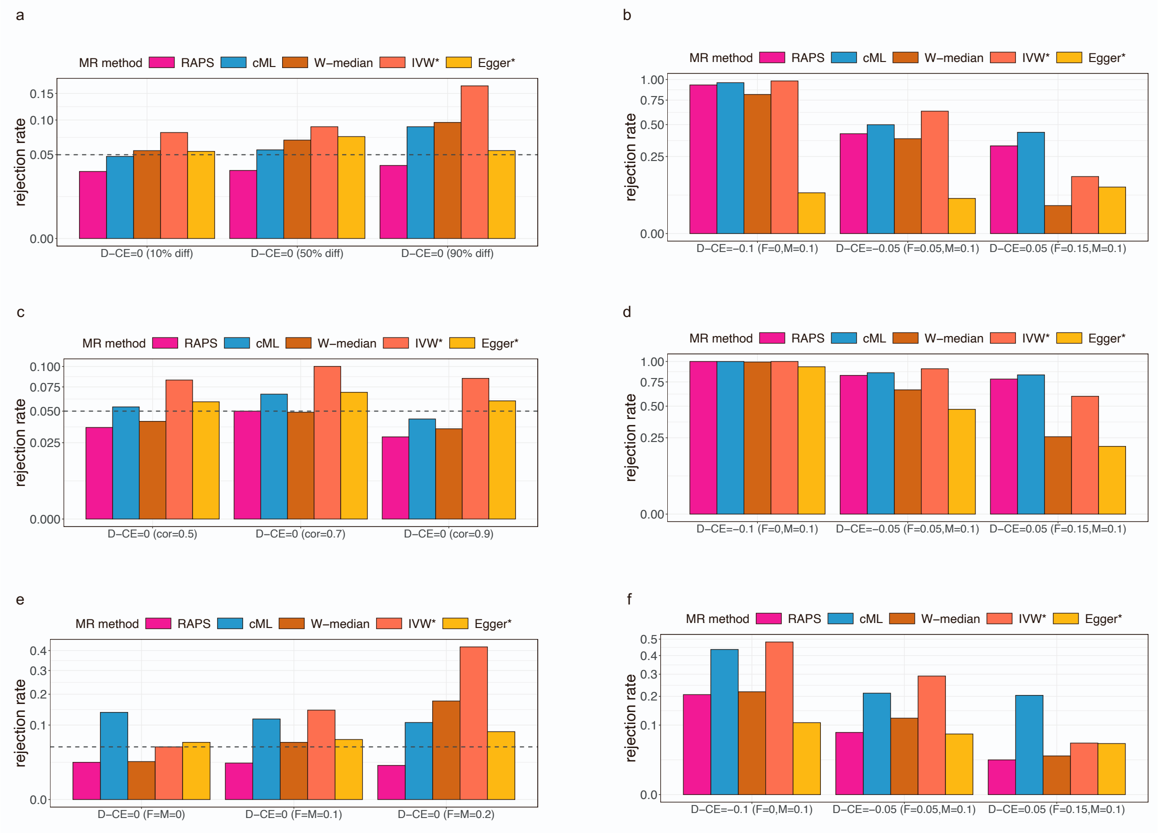

The figure shows the rejection rates of the sex-differences test computed by each MR method across 1000 simulation replicates in primary simulation studies. The left panels (a, c, e) provide results from settings with no sex differences in the causal effect (i.e., measuring type 1 error), while the panels on the right (b, d, f) provide results from settings with sex differences in the causal effects (i.e., measuring power). Both female and male causal effects are set as 0.1 in panel a and c. In panel e, the underlying causal effect is shown in parentheses below (x-axis labels) in the form (F (β_F) = M (β_M) = value). The values of female and male-specific causal effects in sex-differences settings are shown at the bottom of each sub-panel (sub-panel b, d, and f) and are specified as D-CE=value. The horizontal dashed line at 0.05 in sub-panel a, c, and e indicates the desired type 1 error rate. We used five two-sample MR methods to estimate sex-specific causal effect: W-median, IVW*, Egger*, cML, and RAPS. The contaminated mixture approach was not included in this analysis because the algorithm does output the standard error of the estimated causal effect. All methods used the raw variant-exposure effect estimate to conduct sex differences tests.

Abbreviations: D-CE: differences in causal effect; CE: causal effect; MR: Mendelian randomization; diff: different level of sex differences in exposure effects; Cor: correlation between female and male exposure effect; W-median: weighted median; IVW*: penalized and robust IVW; Egger*: penalized and robust MR-Egger; cML: constrained maximum likelihood; RAPS: MR-RAPS; F: female; M: Male.

Figure S16: Rejection rates of sex-differences test from secondary simulation 4 (equivalent female-male sample size)

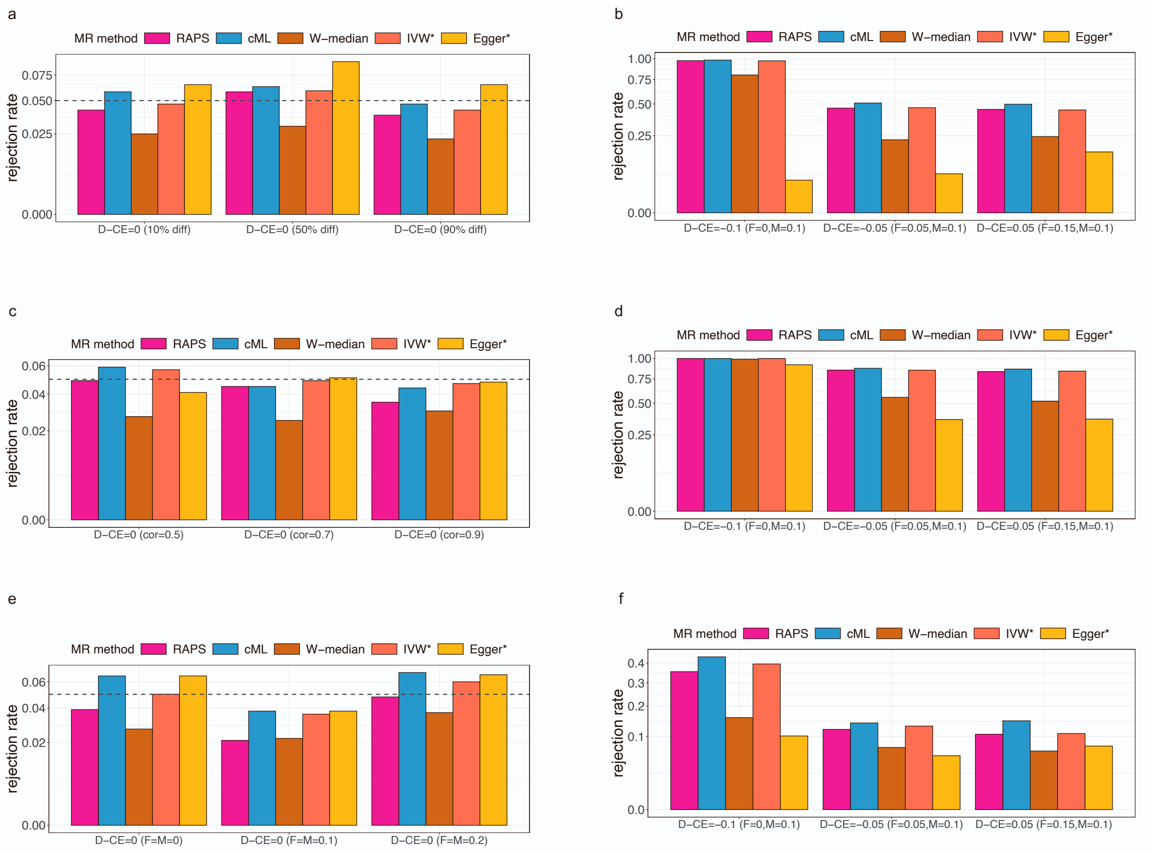

The figure shows the rejection rates of the sex-differences test computed by each MR method across 1000 simulation replicates in increasing female sample size settings. The left panels (a, c, e) provide results from settings with no sex differences in the causal effect (i.e., measuring type 1 error), while the panels on the right (b, d, f) provide results from settings with sex differences in the causal effects (i.e., measuring power). Both female and male causal effects are set as 0.1 in panel a and c. In panel e, the underlying causal effect is shown in parentheses below (x-axis labels) in the form (F (β_F) = M (β_M) = value). The values of female and male-specific causal effects in sex-differences settings are shown at the bottom of each sub-panel (sub-panel b, d, and f) and are specified as D-CE=value. The horizontal dashed line at 0.05 in sub-panel a, c, and e indicates the desired type 1 error rate. We used five two-sample MR methods to estimate sex-specific causal effect: W-median, IVW*, Egger*, cML, and RAPS. The contaminated mixture approach was not included in this analysis because the algorithm does output the standard error of the estimated causal effect. All methods used the raw variant-exposure effect estimate to conduct sex differences tests.

Abbreviations: D-CE: differences in causal effect; CE: causal effect; MR: Mendelian randomization; diff: different level of sex differences in exposure effects; Cor: correlation between female and male exposure effect; W-median: weighted median; IVW*: penalized and robust IVW; Egger*: penalized and robust MR-Egger; cML: constrained maximum likelihood; RAPS: MR-RAPS; F: female; M: Male.

Note S3: Secondary data analyses

The sex-specific causal effect estimates using $\hat{\gamma}_{raw}$, $\hat{\gamma}_{AW}$, and $\hat{\gamma}_{meta}$, as well as comparisons between male and female-specific causal effect, are shown in Figures S17-20. Results based on $\hat{\gamma}_{raw}$ for IV selection are shown in Figures S17 and 18, while those using $\hat{\gamma}_{APM}$ for IV selection are provided in Figure S19 and S20. The number of selected IVs are listed in Table S4. The comparisons of instrumental variable strength, measured by the average *F*-statistic, using both $\hat{\gamma}_{raw}$ and $\hat{\gamma}_{APM}$ for IV selection are summarized in Table S5.

Table S4: The number of variants passing p-value thresholding and clumping procedure in each MVP sleep GWAS

	male selection	female selection	APM male selection	APM female selection	FE meta selection
OSA bmi-unadjusted	122	17	155	51	134
OSA bmi-adjusted	70	14	86	19	73
Insomnia bmi-unadjusted	23	15	30	12	22
Insomnia bmi-adjusted	21	15	29	13	22
Sleepiness bmi-unadjusted	31	12	42	19	29
Sleepiness bmi-adjusted	26	13	34	15	20
Short sleep bmi-unadjusted	41	12	55	16	47
Short sleep bmi-adjusted	41	13	47	18	43
Long sleep bmi-unadjusted	25	18	34	15	27
Long sleep bmi-adjusted	25	19	36	14	29

Note: The p-value threshold was 10^{-5}.

Table S5: Average *F*-statistic for variants selected by raw and APM estimates in MVP sleep GWASs

	BMI-unadjusted		BMI-adjusted	
	raw	APM	raw	APM
**Female raw selection**				
OSA	20.6	19.8	20.9	20.1
Insomnia	20.9	20.2	20.9	20.3
Long Sleep	20.7	19.9	20.6	19.8
Short Sleep	21.9	21.2	21.4	20.7
Sleepiness	20.7	19.9	20.7	19.9
**Male raw selection**				
OSA	22.3	23.0	21.0	21.8
Insomnia	20.9	21.7	21.0	21.9
Long Sleep	21.7	22.3	21.8	22.3
Short Sleep	21.0	21.9	20.9	21.7
Sleepiness	21.1	21.9	21.2	22.1
**Female APM selection**				
OSA	4.9	22.7	11.0	20.9
Insomnia	15.9	22.3	16.6	22.2
Long Sleep	17.6	20.7	17.4	20.8
Short Sleep	14.8	21.4	13.1	21.1
Sleepiness	12.2	20.9	13.7	21.2
**Male APM selection**				
OSA	21.3	22.1	20.3	21.1
Insomnia	19.9	20.8	20.1	21.1
Long Sleep	20.6	21.3	20.8	21.5
Short Sleep	20.1	21.0	20.3	21.2
Sleepiness	20.2	21.1	20.4	21.3

Note: These results represent the average *F*-statistic computed across all selected variants in each analysis. The number of variants used in each analysis is listed in Table S4.

Comparing results between MR-RAPS and MR-PRESSO

In all analyses, we set 10^{-5} as the p-value threshold to select IVs. This decision is due to the fact that no variants were available in the female population when a stricter p-value threshold was considered. This lower p-value threshold, however, may increase the risk of including weak IVs as well as the possibility of variants having horizontal pleiotropy effect due to the larger number of variants used as IVs. Therefore, we compared the findings from primary analyses, the causal estimation from MR-RAPS with $\hat{\gamma}_{APM}$, to MR-PRESSO, which is designed to detect the violation of horizontal pleiotropy assumption in MR analysis. The comparisons are shown in Figures S21 and S22. Overall, the causal effect estimates are consistent between two methods, especially for

those statistically significant findings identified from MR-RAPS. These results increase the reliability of our findings in primary analyses.

Comparing results between raw and APM-derived IV selection strategies

We compared estimated causal effects when using IV selected based on $\hat{\gamma}_{raw}$ and $\hat{\gamma}_{APM}$, as shown in Figure S23. As expected, the resulting estimates were generally consistent with each other in the male population, as it has substantially larger sample size than the female population, so that $\hat{\gamma}_{M,APM}$ tend to be similar to $\hat{\gamma}_{M,raw}$. In contrast, using $\hat{\gamma}_{F,APM}$ identifies several additional IVs, compared to $\hat{\gamma}_{F,raw}$. Therefore, some causal effects were statistically significant only when using $\hat{\gamma}_{F,APM}$ for IV selection.

Results of sex-combined causal effect estimation

We estimated sex-combined causal effects in secondary analysis, using $\hat{\gamma}_{meta}$ estimates. In this case, we also used $\hat{\gamma}_{meta}$ for IV selection. For the outcome phenotype, we conduct sex-combined association analysis for both BMI adjustment and un-adjustment analysis, but further adjust sex variable in the analysis. The results are shown in Figure S24. In this analysis, several causal effects between OSA and CVD-related outcomes, such as CKD, HF, and T2DM, were identified as statistically significant associations. The causal effect of OSA on CKD and on HF were also identified in male population in sex-specific analysis (primary analysis). However, for example, the significant female-specific causal effect of OSA on HTN were not identified in sex-combined analysis. This may be due to the fact that $\hat{\gamma}_{meta}$ is closer to $\hat{\gamma}_{M}$ estimates, caused by the predominance male sample size in MVP.

Figure S17: Results from sex-specific causal effect estimation using BMI-unadjusted sleep GWASs when IVs were selected based on raw associations

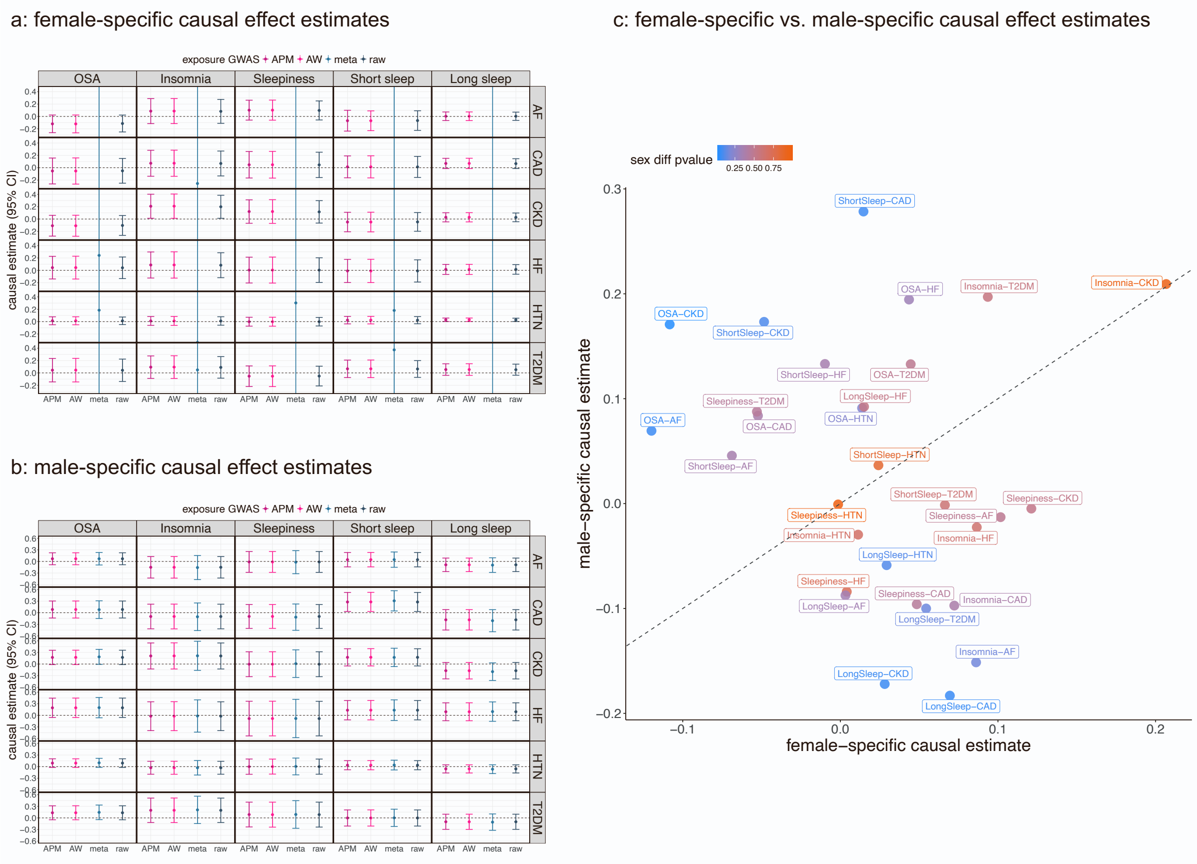

Panels a (female-specific) and b (male-specific) provide sex-specific causal effect (β_F, β_M) estimates with the corresponding 95% CIs. The causal effects were estimated using MR-RAPS and are displayed on the log(OR) scale. Horizontal dashed lines indicate the null value. These analyses used summary statistics from BMI-unadjusted sleep GWASs. IVs were selected based on raw estimates ($\hat{\gamma}_{raw}$). The columns represent the exposure variables, while the rows show the outcome variables. Due to the large standard errors of the $\hat{\beta}_{F,meta}$ estimates (estimates that used $\hat{\gamma}_{meta}$), the corresponding CIs were truncated. Panel c compares estimated β_F (x-axis) and β_M (y-axis) obtained when using APM estimates ($\hat{\gamma}_{APM}$). The color of each point in panel c is proportional to the p-value of the sex-differences test.

Abbreviations: CI: confidence interval; IV: instrumental variable; OR: odds ratio; MR: Mendelian randomization; MR-RAPS: MR using robust adjusted profile score method; APM: adaptive posterior mean; AW: adaptive weight; meta: fixed-effect meta estimates; sex diff p-value: p-value of sex differences test; OSA: obstructive sleep apnea; sleepiness: excessive daytime sleepiness; AF: atrial fibrillation; CAD: coronary artery disease; CKD: chronic kidney disease; HF: heart failure; HTN: hypertension; T2DM: type 2 diabetes mellitus.

Figure S18: Results from sex-specific causal effect estimation using BMI-adjusted sleep GWASs when IVs were selected based on raw associations

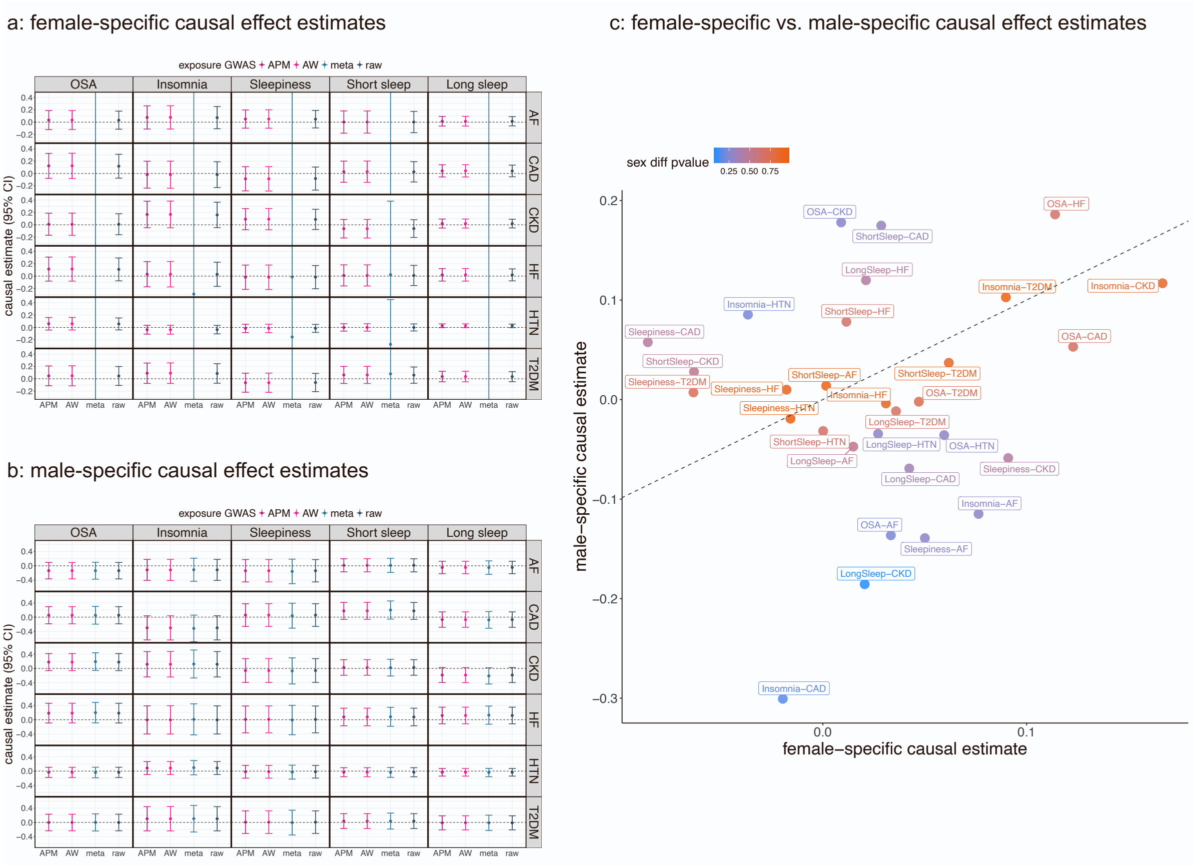

Panels a (female-specific) and b (male-specific) provide sex-specific causal effect (β_F, β_M) estimates with the corresponding 95% CIs. The causal effects were estimated using MR-RAPS and are displayed on the log(OR) scale. Horizontal dashed lines indicate the null value. These analyses used summary statistics from BMI-adjusted sleep GWASs. IVs were selected based on raw estimates ($\hat{\gamma}_{raw}$). The columns represent the exposure variables, while the rows show the outcome variables. Due to the large standard errors of the $\hat{\beta}_{F,meta}$ estimates (estimates that used $\hat{\gamma}_{meta}$), the corresponding CIs were truncated. Panel c compares estimated β_F (x-axis) and β_M (y-axis) obtained when using APM estimates ($\hat{\gamma}_{APM}$). The color of each point in panel c is proportional to the p-value of the sex-differences test.

Abbreviations: CI: confidence interval; IV: instrumental variable; OR: odds ratio; MR: Mendelian randomization; MR-RAPS: MR using robust adjusted profile score method; APM: adaptive posterior mean; AW: adaptive weight; meta: fixed-effect meta estimates; sex diff p-value: p-value of sex differences test; OSA: obstructive sleep apnea; sleepiness: excessive daytime sleepiness; AF: atrial fibrillation; CAD: coronary artery disease; CKD: chronic kidney disease; HF: heart failure; HTN: hypertension; T2DM: type 2 diabetes mellitus.

Figure S19: Results from sex-specific causal effect estimation using variants selected by APM estimates (BMI-unadjusted sleep GWASs)

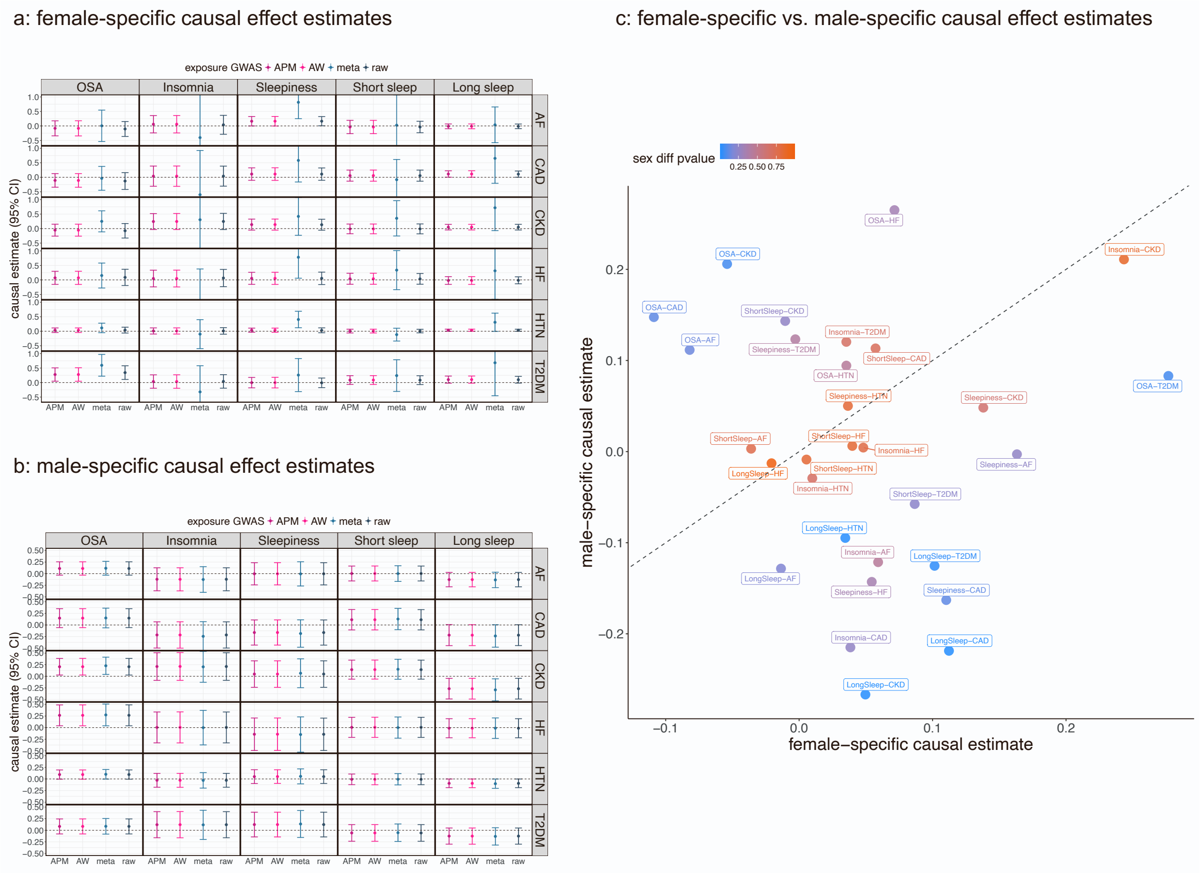

Panels a (female-specific) and b (male-specific) provide sex-specific causal effect (β_F, β_M) estimates with the corresponding 95% CIs. The causal effects were estimated using MR-RAPS and are displayed on the log(OR) scale. Horizontal dashed lines indicate the null value. These analyses used summary statistics from BMI-unadjusted sleep GWASs. IVs were selected based on APM estimates ($\hat{\gamma}_{APM}$). The columns represent the exposure variables, while the rows show the outcome variables. Due to the large standard errors of the $\hat{\beta}_{F,meta}$ estimates (estimates that used $\hat{\gamma}_{meta}$), the corresponding CIs were truncated. Panel c compares estimated β_F (x-axis) and β_M (y-axis) obtained when using APM estimates ($\hat{\gamma}_{APM}$). The color of each point in panel c is proportional to the p-value of the sex differences test.

Abbreviations: CI: confidence interval; IV: instrumental variable; OR: odds ratio; MR: Mendelian randomization; MR-RAPS: MR using robust adjusted profile score method; APM: adaptive posterior mean; AW: adaptive weight; meta: fixed-effect meta estimates; sex diff p-value: p-value of sex differences test; OSA: obstructive sleep apnea; sleepiness: excessive daytime sleepiness; AF: atrial fibrillation; CAD: coronary artery disease; CKD: chronic kidney disease; HF: heart failure; HTN: hypertension; T2DM: type 2 diabetes mellitus.

Figure S20: Results from sex-specific causal effect estimation using variants selected by APM estimates (BMI-adjusted sleep GWASs)

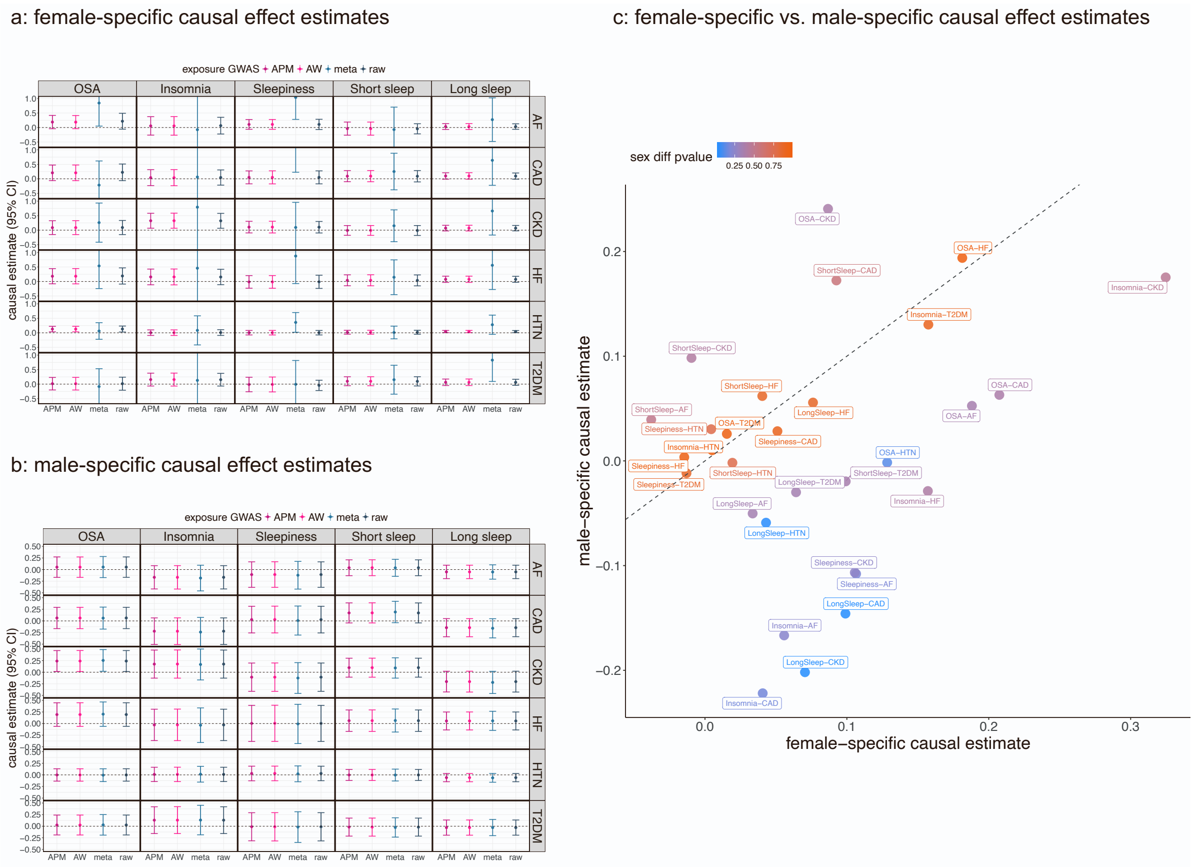

Panels a (female-specific) and b (male-specific) provide sex-specific causal effect (β_F, β_M) estimates with the corresponding 95% CIs. The causal effects were estimated using MR-RAPS and are displayed on the log(OR) scale. Horizontal dashed lines indicate the null value. These analyses used summary statistics from BMI-adjusted sleep GWASs. IVs were selected based on APM estimates ($\hat{\gamma}_{APM}$). The columns represent the exposure variables, while the rows show the outcome variables. Due to the large standard errors of the $\hat{\beta}_{F,meta}$ estimates (estimates that used $\hat{\gamma}_{meta}$), the corresponding CIs were truncated. Panel c compares estimated β_F (x-axis) and β_M (y-axis) obtained when using APM estimates ($\hat{\gamma}_{APM}$). The color of each point in panel c is proportional to the p-value of the sex differences test.

Abbreviations: CI: confidence interval; IV: instrumental variable; OR: odds ratio; MR: Mendelian randomization; MR-RAPS: MR using robust adjusted profile score method; APM: adaptive posterior mean; AW: adaptive weight; meta: fixed-effect meta estimates; sex diff p-value: p-value of sex differences test; OSA: obstructive sleep apnea; sleepiness: excessive daytime sleepiness; AF: atrial fibrillation; CAD: coronary artery disease; CKD: chronic kidney disease; HF: heart failure; HTN: hypertension; T2DM: type 2 diabetes mellitus.

Figure S21: Comparison of causal effect estimates from MR-RAPS and MR-PRESSO using IVs selected based on raw associations

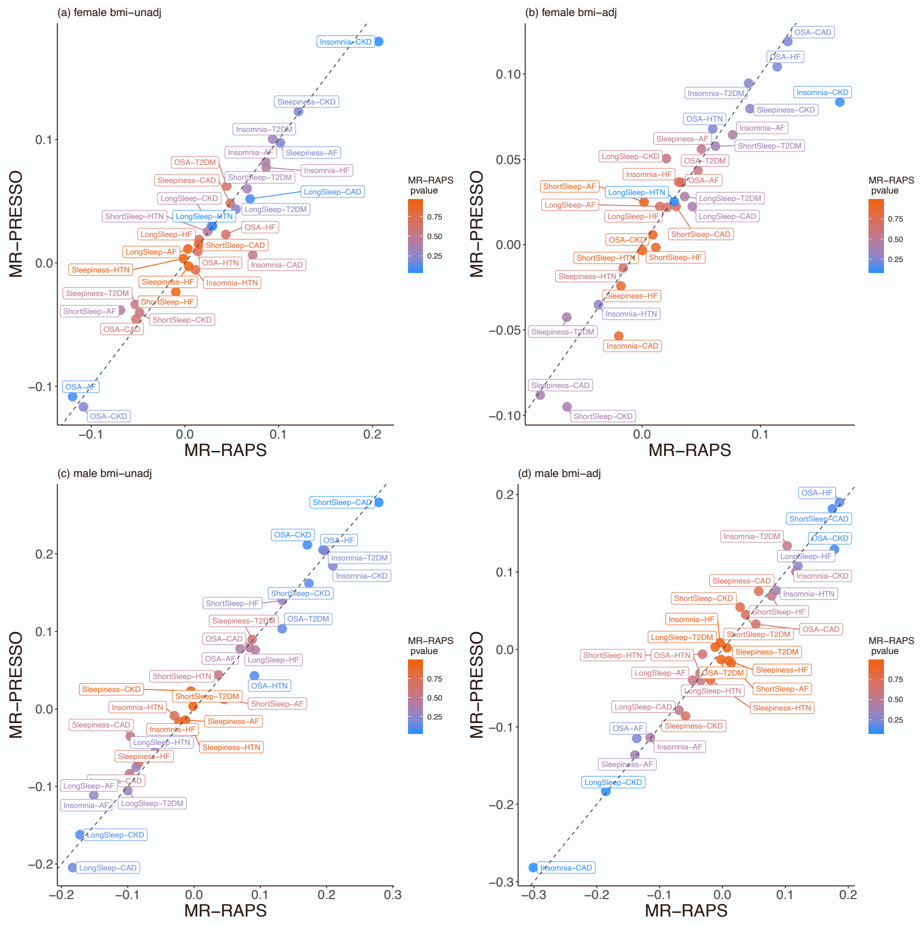

The figure compares of exposure-outcome causal effect (β_F, β_M) estimates between MR-RAPS (x-axis) and MR-PRESSO (y-axis). The IVs were selected based on raw sex-specific variant-exposure estimates ($\hat{\gamma}_{raw}$). The color of each point is proportional to the p-value of the causal effect estimate from MR-RAPS. Panels (a) and (b) show the female-specific results using summary statistics from MVP sleep GWASs without adjustment (panel a) and with adjustment for BMI (panel b), while panels (c) and (d) present the male-specific results without (panel c) and with (panel d) BMI adjustment.

Abbreviation: MR: Mendelian randomization; MR-RAPS: MR using robust adjusted profile score method; MR-PRESSO: Mendelian randomization pleiotropy residual sum and outlier; IV: instrumental variable; OR: odds ratio; MVP: Million Veteran Program; GWAS: Genome-wide association study; bmi-unadj: BMI unadjusted; bmi-adj: BMI adjusted; OSA: obstructive sleep apnea; sleepiness: excessive daytime sleepiness; AF: atrial fibrillation; CAD: coronary artery disease; CKD: chronic kidney disease; HF: heart failure; HTN: hypertension; T2DM: type 2 diabetes mellitus.

Figure S22: Comparison of causal effect estimates from MR-RAPS and MR-PRESSO when using IVs selected by APM estimates

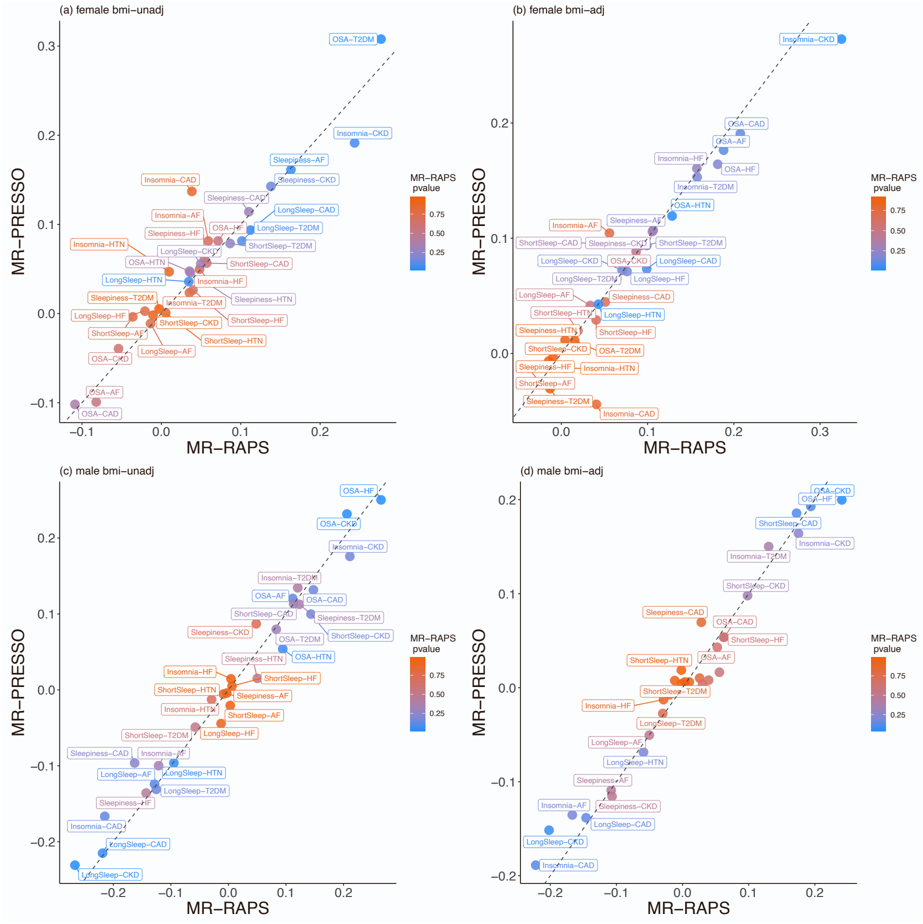

The figure compares exposure-outcome causal effect (β_F, β_M) estimates between MR-RAPS (x-axis) and MR-PRESSO (y-axis). The IVs were selected based on sex-specific APM estimates ($\hat{\gamma}_{APM}$). Estimated effects are displayed on the log(OR) scale. The color of each point is proportional to the p-value of the causal effect estimate from MR-RAPS. Panels (a) and (b) show the female-specific results using summary statistics from MVP sleep GWASs without adjustment (panel a) and with adjustment for BMI (panel b), while panels (c) and (d) present the male-specific results without (panel c) and with (panel d) BMI adjustment.

Abbreviations: MR: Mendelian randomization; MR-RAPS: MR using robust adjusted profile score method; MR-PRESSO: Mendelian randomization pleiotropy residual sum and outlier; IV: instrumental variable; APM: adaptive posterior mean; OR: odds ratio; MVP: Million Veteran Program; GWAS: Genome-wide association study; bmi-unadj: BMI unadjusted; bmi-adj: BMI adjusted; OSA: obstructive sleep apnea; sleepiness: excessive daytime sleepiness; AF: atrial fibrillation; CAD: coronary artery disease; CKD: chronic kidney disease; HF: heart failure; HTN: hypertension; T2DM: type 2 diabetes mellitus.

Figure S23: Comparison of causal effect estimates using raw and APM-derived IV selection strategies

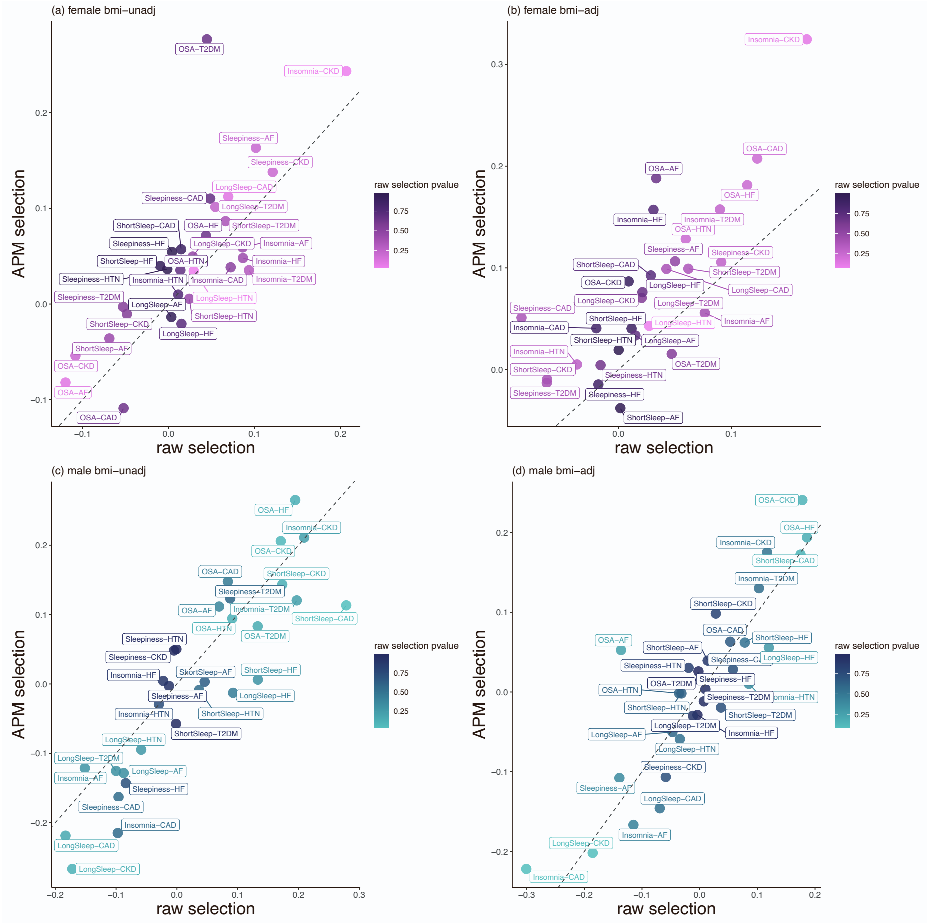

The figure compares exposure-outcome causal effect (β_F, β_M) estimates from using (i) raw estimate ($\hat{\gamma}_{raw}$) and (ii) APM ($\hat{\gamma}_{APM}$) estimate for IV selection. Causal effects were estimated using MR-RAPS with $\hat{\gamma}_{APM}$ and are displayed on a log(OR) scale. The x-axis shows causal effect estimates based on IVs selected using $\hat{\gamma}_{raw}$, while y-axis shows the results for IV selected by $\hat{\gamma}_{APM}$ estimates. Panels (a) and (b) show the female-specific results. Panel (a) used the summary statistics from BMI-unadjusted sleep GWASs, while panel (b) used the summary statistics from BMI-adjusted sleep GWASs. Panels (c) and (d) display the male-specific results, with panel (c) using the summary statistics from BMI-unadjusted sleep GWASs and panel (d) using the summary statistics from BMI-adjusted sleep GWASs. The color of each point is proportional to the p-value of the causal effect estimate from MR-RAPS in primary analysis.

Abbreviations: APM: adaptive posterior mean; IV: instrumental variable; MR-RAPS: MR using robust adjusted profile score method; OR: odds ratio; GWAS: Genome-wide association study; bmi-unadj: BMI unadjusted; bmi-adj: BMI adjusted; OSA: obstructive sleep apnea; sleepiness: excessive daytime sleepiness; AF: atrial fibrillation; CAD: coronary artery disease; CKD: chronic kidney disease; HF: heart failure; HTN: hypertension; T2DM: type 2 diabetes mellitus.

Figure S24: Results from sex-combined causal effect estimation using variants selected by fixed-effect meta estimates

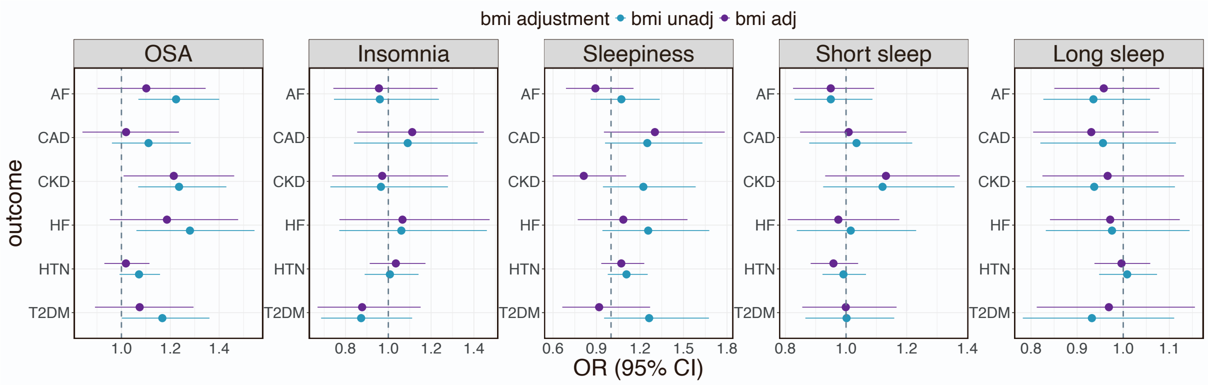

This figure presents sex-combined causal effect estimates with corresponding 95% CIs, based on IVs selected using $\hat{\gamma}_{meta}$. Variant-outcome association effect sizes were computed from a sex-combined dataset in the AoU study. The estimated causal effects, obtained via MR-RAPS using $\hat{\gamma}_{meta}$ variant-exposure effect estimates, are displayed on an OR scale. In each figure, variant-phenotype estimates without BMI adjustment are shown in blue, while those with BMI adjustment are shown in purple. Vertical dashed lines represent the null causal effect. Exposure variables are labeled at the top of each figure, with outcome variables displayed across the rows.

Abbreviations: CI: confidence interval; IV: instrumental variable; AoU: All of Us; MR-RAPS: MR using robust adjusted profile score method; MR: Mendelian randomization; OR: odds ratio; BMI: body mass index; bmi unadj: BMI-unadjusted; bmi adj: BMI-adjusted; OSA: obstructive sleep apnea; AF: atrial fibrillation; CAD: coronary artery disease; CKD: chronic kidney disease; HF: heart failure; HTN: hypertension; T2DM: type 2 diabetes mellitus.

Multivariable MR analysis

We conducted multivariable MR (MVMR) as a sensitivity analysis to estimate the direct causal effects of sleep phenotypes on CVD outcomes, adjusting for BMI as a secondary exposure. BMI was included due to its strong correlation with sleep traits and its established role as a potential confounder (in both sleep and CVD analysis). We used BMI-unadjusted variant-trait effect estimates for both the sleep exposures and CVD outcomes in this analysis. BMI summary statistics were downloaded from a meta-analysis of individuals of European ancestry in the GIANT consortium and UK Biobank (https://zenodo.org/records/1251813#.XCLJ7vZKhE4).

We used the same set of genetic variants as IVs from the primary BMI-unadjusted analysis, as we see the sleep traits as the primary exposures of interest. We then extracted the corresponding variant-exposure associations from BMI summary statistics. Since the summary statistics for sleep traits and BMI were derived from different studies, we harmonized the effect alleles using the harmonise_data() function in the TwoSampleMR R package. After that, we then harmonized the exposures and outcome using the mv_harmonise_data() function. MVMR analysis was then performed using the robust multivariable inverse-variance weighted (MV-IVW) method. The results from both robust MV-IVW and the MV-IVW were reported in Supplemental Data 3. We implemented two IV selection strategies based on either the $\hat{\gamma}_{raw}$ or the $\hat{\gamma}_{APM}$. When IVs were selected using $\hat{\gamma}_{raw}$, all variant-exposure associations, including both sleep traits and BMI, were estimated using the “un-calibrated” variant-exposure effect estimates. When IVs were selected based on $\hat{\gamma}_{APM}$, variant-exposure associations for sleep traits were based on $\hat{\gamma}_{APM}$, while BMI continued to use $\hat{\gamma}_{raw}$. This choice is due to the point that we

are focusing on improving inference for the primary exposure (sleep traits), while treating BMI as a secondary exposure and maintaining analytical simplicity.

The results are presented in Figure S25, and Table S6 summarizes the significant findings from the MVMR analysis. Compared with the primary analysis, several significant associations were consistent with those observed in the BMI-unadjusted GWAS analysis. For example, we observed an increased causal effect of insomnia on CKD in females, an increased risk of short sleep associated with CAD in males, and a protective male-specific effect of long sleep duration on both CKD and HTN. Some significant findings were identified only in either the univariable or the MVMR analyses. For instance, the male-specific causal effect of OSA on HTN and the female-specific causal effect of OSA on T2DM were detected only in the univariable analysis. In contrast, the male-specific causal effect of OSA on CAD was identified only in the MVMR analysis. Overall, the direction of the estimated causal effects was consistent between the MVMR and BMI-unadjusted univariable MR analyses, regardless of statistical significance.

Less consistent findings were found in the comparison between MVMR with the univariable BMI-adjusted analysis. When IVs were selected based on raw association ($\hat{\gamma}_{raw}$), no significant findings were observed in univariable BMI-adjusted analysis, however, MVMR identified several sex-specific effects (e.g., in males: OSA on CAD, short sleep on CAD, long sleep on CKD; in females: insomnia on CKD). In contrast, the significant female-specific causal effects of OSA on HTN and long sleep on HTN were only identified in the BMI-adjusted univariable analysis but

not in MVMR. The differences of causal effect estimation may possibly come from the choice of IVs in each analysis, as the IVs used in MVMR were derived from BMI-unadjusted sleep GWASs.

Figure S26 presents a comparison between the robust MV-IVW method and the MR-RAPS method (from primary analysis), using IVs selected based on raw associations ($\hat{\gamma}_{raw}$). Results based on IVs selected using APM estimates ($\hat{\gamma}_{APM}$) are shown in Figure S27. For the MVMR analyses, IVs were used from BMI-unadjusted sleep GWASs, and the results were compared with those from primary analyses (MR-RAPS) using both BMI-unadjusted and BMI-adjusted sleep GWAS.s The robust MV-IVW estimates were more closely aligned with the BMI-unadjusted results and showed less concordance with the BMI-adjusted results, in both female and male analyses.

Table S6: Top findings from sex-specific MVMR exposure-outcome causal effect estimation

	Exposure	Outcome	$exp\{\hat{\beta}_F\}$	$exp\{\hat{\beta}_M\}$
$\hat{\gamma}_{raw}$ selection				
	Insomnia	CKD	**1.22 (1.03, 1.43)**	**1.32 (1.00, 1.74)**
	Long sleep	CKD	1.02 (0.94, 1.10)	**0.75 (0.63, 0.89)**
	Short sleep	CAD	0.99 (0.89, 1.10)	**1.33 (1.03, 1.73)**
	OSA	CAD	1.02 (0.88, 1.19)	**1.38 (1.08, 1.79)**
$\hat{\gamma}_{APM}$ selection				
	Long Sleep	CAD	**1.16 (1.08, 1.24)**	**0.77 (0.61, 0.97)**
	Long Sleep	CKD	0.98 (0.90, 1.08)	**0.71 (0.58, 0.87)**
	Long Sleep	HTN	1.02 (0.98, 1.06)	**0.90 (0.83, 0.98)**
	OSA	CAD	0.89 (0.68, 1.16)	**1.31 (1.04, 1.66)**
	Insomnia	CKD	1.26 (0.95, 1.67)	**1.33 (1.01, 1.75)**

Selected findings from sex-specific MVMR exposure-outcome causal effect estimation, where significant results were identified in at least one sex-stratum, using robust MV-IVW. The results based on $\hat{\gamma}_{raw}$ for IV selection are shown in the top three rows, while the remaining results are from the analyses using $\hat{\gamma}_{APM}$ for IV selection. The exposure and outcome of interest are listed in the second and third columns, respectively. The estimated causal effects of sleep exposure on CVD outcome are given in an OR scale ($exp\{\beta\}$). The female-specific causal estimates and the corresponding 95% confidence intervals are shown in the fourth column, and the results for males are in the fifth column. Bold values indicate statistically significant results (p-value < 0.05).

Abbreviations: MVMR: multivariable Mendelian randomization; MV-IVW: multivariable inverse-variance weighted method; APM: adaptive posterior mean; IV: instrumental variable; OR: odds ratio; OSA: obstructive sleep apnea; CAD: coronary artery disease; CKD: chronic kidney disease; HTN: hypertension.

Figure S25: Results from sex-specific MVMR causal effect estimation

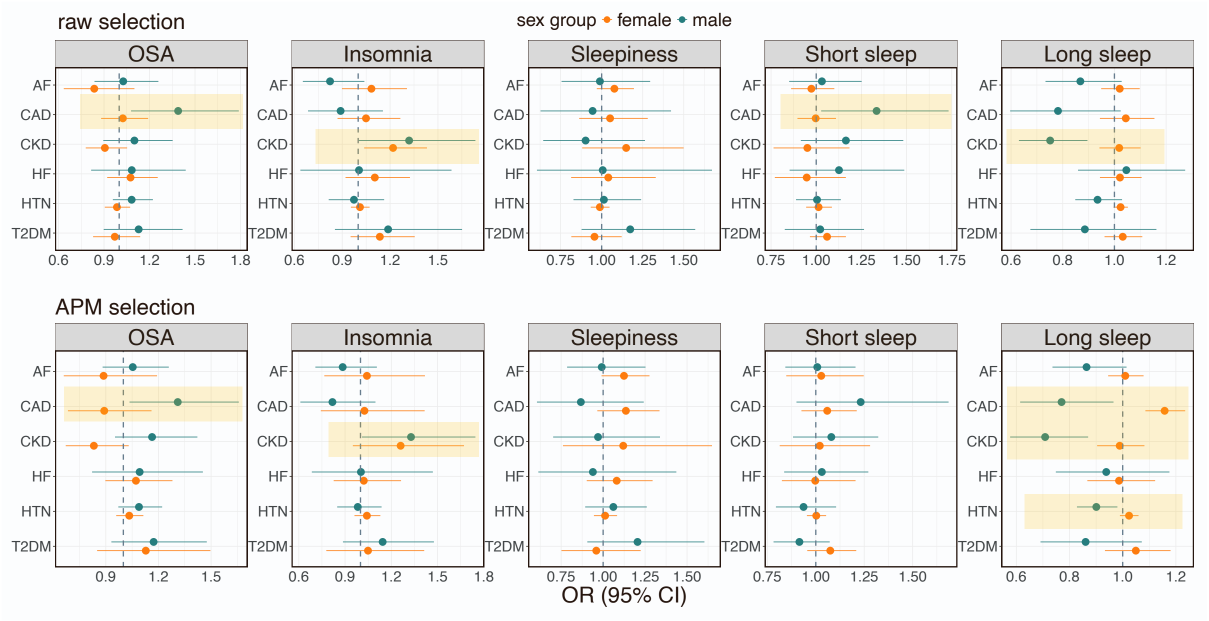

This figure presents sex-specific causal effect estimates of sleep exposures on CVD outcomes, by adjusting BMI as secondary exposure, from MVMR analysis. The estimated causal effects (from robust MV-IVW) are displayed on an OR scale, with the corresponding 95% CIs. Top panel provides results based on IVs selected by using $\hat{\gamma}_{raw}$, while bottom panel shows result using $\hat{\gamma}_{APM}$ for IV selection. For both sleep and CVD summary statistics, the variant-trait effect estimates were estimated without adjusting for BMI. Female-specific results are indicated in the color orange, while results from male-specific are shown in color green. Vertical dashed lines indicate the null causal effects (OR = 1). The exposure variables are shown as the titles of the sub-panels, while row names of the sub-panels provide the outcome variables. Statistically significant results (p-value < 0.05) for either the female or male-specific analysis are highlighted with yellow background.

Abbreviations: MVMR: multivariable Mendelian randomization; CI: confidence interval; APM: adaptive posterior mean; IV: instrumental variable; MV-IVW: multivariable inverse-variance weighted method; OR: odds ratio; BMI: body mass index; CVD: cardiovascular disease; OSA: obstructive sleep apnea; AF: atrial fibrillation; CAD: coronary artery disease; CKD: chronic kidney disease; HF: heart failure; HTN: hypertension; T2DM: type 2 diabetes mellitus.

Figure S26: Comparison of causal effect estimates from robust MV-IVW and MR-RAPS when using IVs selected by raw associations

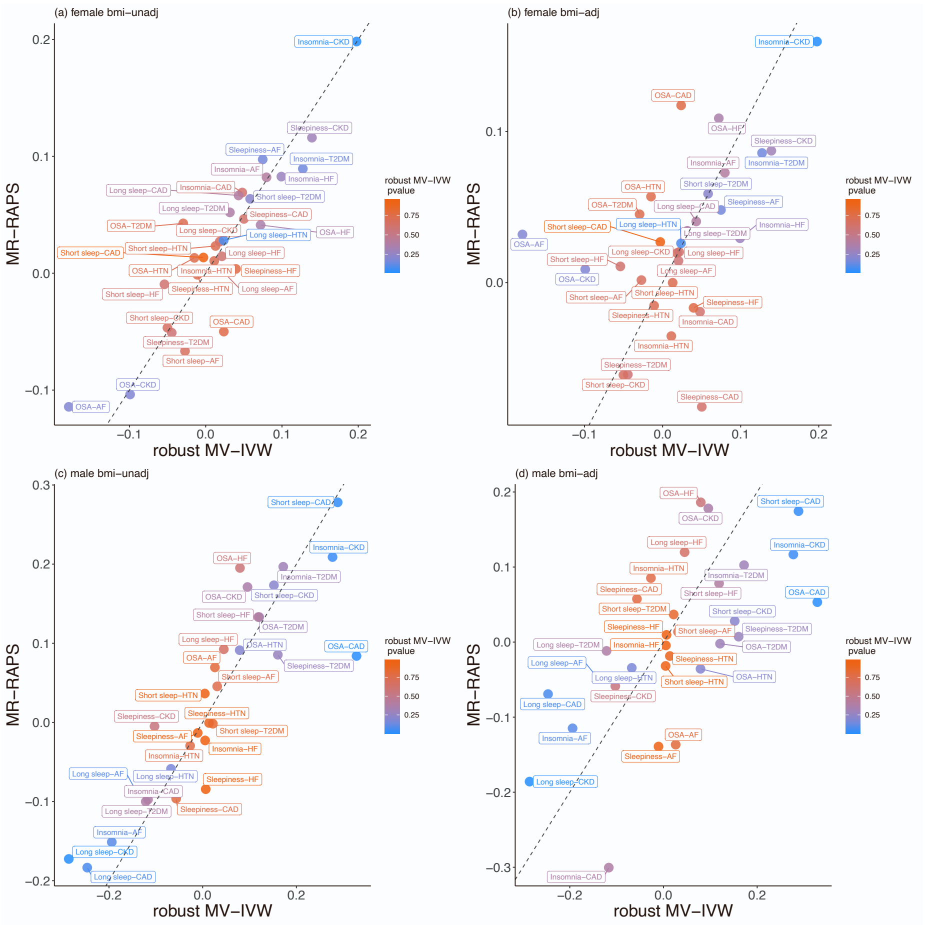

The figure compares exposure-outcome causal effect (β_F, β_M) estimates between MVMR (x-axis: robust MV-IVW and univariable MR (y-axis: MR-RAPS). The IVs were selected based on sex-specific variant-exposure estimates ($\hat{\gamma}_{raw}$). Estimated effects are displayed on the log(OR) scale. The color of each point is proportional to the p-value of the causal effect estimate from robust MV-IVW. All results from robust MV-IVW are based on the BMI-unadjusted MVP sleep GWAS. The summary statistics used for MR-RAPS are indicated at the top of each panel. Panels (a) and (b) show female-specific results using summary statistics from MVP sleep GWAS without BMI adjustment (panel a) and with BMI adjustment (panel b), while panels (c) and (d) show male-specific results without (panel c) and with (panel d) BMI adjustment.

Abbreviations: MR: Mendelian randomization; MVMR: multivariable MR; MV-IVW: multivariable inverse-variance weighted method; MR-RAPS: MR using robust adjusted profile score method; IV: instrumental variable; OR: odds ratio; MVP: Million Veteran Program; GWAS: Genome-wide association study; bmi-unadj: BMI unadjusted; bmi-adj: BMI adjusted; OSA: obstructive sleep apnea; sleepiness: excessive daytime sleepiness; AF: atrial fibrillation; CAD: coronary artery disease; CKD: chronic kidney disease; HF: heart failure; HTN: hypertension; T2DM: type 2 diabetes mellitus.

Figure S27: Comparison of causal effect estimates from robust MV-IVW and MR-RAPS when using IVs selected by APM estimates

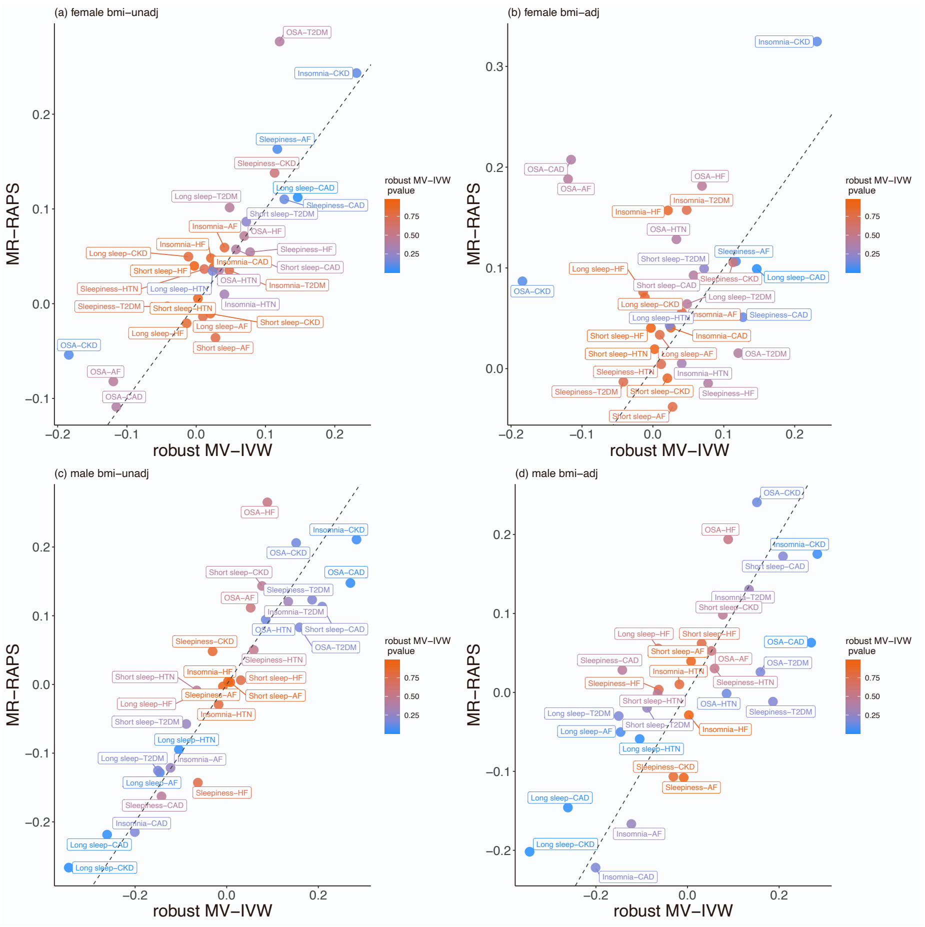

The figure compares exposure-outcome causal effect (β_F, β_M) estimates between MVMR (x-axis: robust MV-IVW and univariable MR (y-axis: MR-RAPS). The IVs were selected based on sex-specific APM estimates ($\hat{\gamma}_{APM}$). Estimated effects are displayed on the log(OR) scale. The color of each point is proportional to the p-value of the causal effect estimate from robust MV-IVW. All results from robust MV-IVW are based on the BMI-unadjusted MVP sleep GWAS. The summary statistics used for MR-RAPS are indicated at the top of each panel. Panels (a) and (b) show female-specific results using summary statistics from MVP sleep GWAS without BMI adjustment (panel a) and with BMI adjustment (panel b), while panels (c) and (d) show male-specific results without (panel c) and with (panel d) BMI adjustment.

Abbreviations: MR: Mendelian randomization; MVMR: multivariable MR; MV-IVW: multivariable inverse-variance weighted method; MR-RAPS: MR using robust adjusted profile score method; IV: instrumental variable; APM: adaptive posterior mean; OR: odds ratio; MVP: Million Veteran Program; GWAS: Genome-wide association study; bmi-unadj: BMI unadjusted; bmi-adj: BMI adjusted; OSA: obstructive sleep apnea; sleepiness: excessive daytime sleepiness; AF: atrial fibrillation; CAD: coronary artery disease; CKD: chronic kidney disease; HF: heart failure; HTN: hypertension; T2DM: type 2 diabetes mellitus.

Note S4: All of Us genomics and phenotype data

We used short-read whole-genome sequencing (srWGS) data (version 7) from the All of Us (AoU) study to estimate single variant association analysis with CVD-related outcomes. Report of sequencing and quality control methods are provided in this link: https://support.researchallofus.org/hc/article_attachments/19370367115796. AoU provided genetics PCs and performed relatedness analysis. The genetic PCs information for each individual can be found in: gs://fc-aou-datasets-controlled/v7/wgs/short_read/snpindel/aux/ancestry/ancestry_preds.tsv. The relatedness kinship score can be found in gs://fc-aou-datasets-controlled/v7/wgs/short_read/snpindel/aux/relatedness/relatedness.tsv. We use the information provided in gs://fc-aou-datasets-controlled/v7/wgs/short_read/snpindel/aux/relatedness/relatedness_flagged_samples.tsv to remove related individuals in our analysis. The data were accessed on October, 2024.

Preprocess of clinical variables and six binary CVD-related outcomes in AoU

We first loaded basic clinical information into a Jupyter notebook, including BMI, date of birth, sex assigned at birth, and race/ethnicity for all 245,394 individuals with srWGS data. For the analysis, we focused on six binary CVD-related outcomes: atrial fibrillation (AF), coronary artery disease (CAD), cardiovascular disease (CVD), heart failure (HF), hypertension (HTN), and type 2 diabetes mellitus (T2DM). The outcome data were loaded by selecting the corresponding SNOMED and OMOP Concept IDs for each phenotype, as detailed in Table S7. Individuals without a diagnosis of the specified outcomes were classified as controls. For individuals

diagnosed with a CVD-related condition, we defined their age based on the first recorded diagnosis date and used the closest BMI measurement to that date as the BMI value for further analysis. For control individuals, we define their age as “age at 2024”, and BMI as the value from their earliest recorded measurement.

Table S7: Standard concept names used to define outcome phenotypes in All of Us study

Outcome	OMOP concept ID	SNOMED ID	Number of cases
Atrial fibrillation (AF)	313217	49436004	7,247
Coronary arteriosclerosis (CAD)	317576	53741008	4,870
Chronic Kidney disease (CKD)	46271022	709044004	4,974
Essential hypertension (HTN)	320128	59621000	39,934
Heart failure (HF)	316139	84114007	3,939
Type 2 diabetes mellitus (T2DM)	201826	44054006	6,010

Note: number of cases are computed only from White individuals

We included only individuals who self-reported their sex assigned at birth as either “Female” or “Male”. To align with summary statistics computed for the White HARE group in the MVP, we restricted our analysis to individuals self-identified as White (from race and ethnicity information), however we did not use genetic ancestry. We further limited the analysis to adults aged 18 to 95 with BMI values between 17 and 55. Additionally, individuals with documented deaths in the EHR record were excluded. After the preprocessing step, approximately 114,000 White individuals remained, consisting of 67,600 females and 46,400 males, with slight variations in sample size depending on the specific phenotype analyzed. The characteristics of the samples used in the association analysis for the AoU study are summarized in Table S8.

Table S8: Characteristic of AoU individuals used in variant-outcome association analysis

AF	Female (N=67,610)	Male (N=46,411)	Overall (N=114,021)
AF = No	64,644 (95.6%)	42,130 (90.8%)	106,774 (93.6%)
AF = yes	2,966 (4.4%)	4,281 (9.2%)	7,247 (6.4%)
Age (overall)			
Mean (SD)	58.5 (16.7)	62.0 (16.3)	59.9 (16.6)
Median [Min, Max]	61.0 [18.0, 95.0]	65.0 [20.0, 95.0]	63.0 [18.0, 95.0]
Age (AF = No)			
Mean (SD)	58.1 (16.8)	61.5 (16.7)	59.4 (16.8)
Median [Min, Max]	61.0 [20.0, 95.0]	65.0 [20.0, 95.0]	62.0 [20.0, 95.0]
Age (AF = Yes)			
Mean (SD)	67.2 (11.8)	67.4 (11.0)	67.4 (11.4)
Median [Min, Max]	69.0 [18.0, 94.0]	68.0 [20.0, 95.0]	68.0 [18.0, 95.0]
BMI (overall)			
Mean (SD)	28.7 (7.13)	28.8 (5.63)	28.8 (6.56)
Median [Min, Max]	27.1 [17.0, 55.0]	27.9 [17.0, 55.0]	27.5 [17.0, 55.0]
BMI (AF = No)			
Mean (SD)	28.6 (7.10)	28.7 (5.58)	28.7 (6.54)
Median [Min, Max]	27.1 [17.0, 55.0]	27.8 [17.0, 55.0]	27.4 [17.0, 55.0]
BMI (AF = Yes)			
Mean (SD)	30.2 (7.63)	30.0 (5.94)	30.1 (6.69)
Median [Min, Max]	28.8 [17.0, 55.0]	28.9 [17.2, 54.9]	28.9 [17.0, 55.0]

CAD	Female (N=67,604)	Male (N=46,408)	Overall (N=114,012)
CAD = No	65,984 (97.6%)	43,158 (93.0%)	109,142 (95.7%)
CAD = Yes	1,620 (2.4%)	3,250 (7.0%)	4,870 (4.3%)
Age (overall)			
Mean (SD)	58.6 (16.7)	62.0 (16.3)	59.9 (16.7)
Median [Min, Max]	61.0 [20.0, 95.0]	65.0 [20.0, 95.0]	63.0 [20.0, 95.0]
Age (CAD = No)			
Mean (SD)	58.4 (16.8)	61.8 (16.7)	59.8 (16.9)
Median [Min, Max]	61.0 [20.0, 95.0]	65.0 [20.0, 95.0]	63.0 [20.0, 95.0]
Age (CAD = Yes)			
Mean (SD)	64.0 (11.0)	64.2 (9.91)	64.2 (10.3)
Median [Min, Max]	65.0 [20.0, 95.0]	65.0 [24.0, 95.0]	65.0 [20.0, 95.0]
BMI			
Mean (SD)	28.7 (7.14)	28.8 (5.63)	28.8 (6.57)
Median [Min, Max]	27.1 [17.0, 55.0]	27.9 [17.0, 55.0]	27.5 [17.0, 55.0]
BMI (CAD = No)			

Mean (SD)	28.7 (7.13)	28.8 (5.65)	28.7 (6.59)
Median [Min, Max]	27.1 [17.0, 55.0]	27.8 [17.0, 55.0]	27.4 [17.0, 55.0]
BMI (CAD = Yes)			
Mean (SD)	30.5 (7.22)	29.7 (5.28)	29.9 (6.01)
Median [Min, Max]	29.4 [17.0, 54.9]	28.8 [17.1, 53.7]	29.0 [17.0, 54.9]

**CKD**	**Female (N=67,573)**	**Male (N=46,391)**	**Overall (N=113,964)**
CKD = No	65,464 (96.9%)	43,526 (93.8%)	108,990 (95.6%)
CKD = Yes	2,109 (3.1%)	2,865 (6.2%)	4,974 (4.4%)
Age (overall)			
Mean (SD)	58.6 (16.8)	62.3 (16.5)	60.1 (16.8)
Median [Min, Max]	61.0 [20.0, 95.0]	66.0 [19.0, 95.0]	63.0 [19.0, 95.0]
Age (CKD = No)			
Mean (SD)	58.4 (16.9)	62.0 (16.7)	59.9 (16.9)
Median [Min, Max]	61.0 [20.0, 95.0]	66.0 [20.0, 95.0]	63.0 [20.0, 95.0]
Age (CKD = Yes)			
Mean (SD)	64.3 (13.2)	66.5 (12.3)	65.5 (12.7)
Median [Min, Max]	66.0 [21.0, 94.0]	68.0 [19.0, 95.0]	67.0 [19.0, 95.0]
BMI (overall)			
Mean (SD)	28.7 (7.14)	28.8 (5.63)	28.8 (6.57)
Median [Min, Max]	27.1 [17.0, 55.0]	27.9 [17.0, 55.0]	27.5 [17.0, 55.0]
BMI (CKD = No)			
Mean (SD)	28.6 (7.10)	28.7 (5.58)	28.7 (6.54)
Median [Min, Max]	27.1 [17.0, 55.0]	27.8 [17.0, 55.0]	27.4 [17.0, 55.0]
BMI (CKD = Yes)			
Mean (SD)	31.3 (7.67)	30.4 (6.10)	30.8 (6.82)
Median [Min, Max]	30.1 [17.0, 54.9]	29.4 [17.0, 54.9]	29.7 [17.0, 54.9]

**HF**	**Female (N=67,582)**	**Male (N=46,380)**	**Overall (N=113,962)**
HF = No	65,871 (97.5%)	44,152 (95.2%)	110,023 (96.5%)
HF = Yes	1,711 (2.5%)	2,228 (4.8%)	3,939 (3.5%)
Age (overall)			
Mean (SD)	58.7 (16.8)	62.5 (16.6)	60.2 (16.8)
Median [Min, Max]	61.0 [20.0, 95.0]	66.0 [20.0, 95.0]	63.0 [20.0, 95.0]
Age (HF = No)			
Mean (SD)	58.5 (16.9)	62.2 (16.7)	60.0 (16.9)
Median [Min, Max]	61.0 [20.0, 95.0]	66.0 [20.0, 95.0]	63.0 [20.0, 95.0]
Age (HF = Yes)			
Mean (SD)	65.5 (13.5)	67.4 (12.4)	66.6 (13.0)
Median [Min, Max]	67.0 [21.0, 95.0]	68.0 [22.0, 95.0]	68.0 [21.0, 95.0]

BMI (overall)			
Mean (SD)	28.7 (7.14)	28.8 (5.63)	28.8 (6.57)
Median [Min, Max]	27.1 [17.0, 55.0]	27.9 [17.0, 55.0]	27.5 [17.0, 55.0]
BMI (HF = No)			
Mean (SD)	28.6 (7.08)	28.7 (5.55)	28.7 (6.51)
Median [Min, Max]	27.1 [17.0, 55.0]	27.8 [17.0, 55.0]	27.4 [17.0, 55.0]
BMI (HF = Yes)			
Mean (SD)	32.5 (8.49)	31.1 (6.66)	31.7 (7.54)
Median [Min, Max]	31.2 [17.0, 55.0]	30.1 [17.1, 54.5]	30.5 [17.0, 55.0]
**HTN**	**Female (N=67,680)**	**Male (N=46,490)**	**Overall (N=114,170)**
HTN = No	46,877 (69.3%)	27,359 (58.8%)	74,236 (65.0%)
HTN = Yes	20,803 (30.7%)	19,131 (41.2%)	39,934 (35.0%)
Age (overall)	46,877 (69.3%)	27,359 (58.8%)	74,236 (65.0%)
Mean (SD)	55.9 (15.9)	58.6 (15.5)	57.0 (15.8)
Median [Min, Max]	58.0 [18.0, 95.0]	61.0 [18.0, 95.0]	59.0 [18.0, 95.0]
Age (HTN = No)			
Mean (SD)	54.8 (16.8)	57.6 (17.3)	55.9 (17.1)
Median [Min, Max]	56.0 [20.0, 95.0]	60.0 [20.0, 95.0]	57.0 [20.0, 95.0]
Age (HTN = Yes)			
Mean (SD)	58.3 (13.0)	60.1 (12.3)	59.2 (12.7)
Median [Min, Max]	60.0 [18.0, 95.0]	62.0 [18.0, 95.0]	61.0 [18.0, 95.0]
BMI (overall)			
Mean (SD)	28.8 (7.16)	28.9 (5.65)	28.8 (6.59)
Median [Min, Max]	27.2 [17.0, 55.0]	27.9 [17.0, 55.0]	27.5 [17.0, 55.0]
BMI (HTN = No)			
Mean (SD)	27.6 (6.67)	27.8 (5.27)	27.7 (6.19)
Median [Min, Max]	26.0 [17.0, 55.0]	26.9 [17.0, 55.0]	26.4 [17.0, 55.0]
BMI (HTN = Yes)			
Mean (SD)	31.4 (7.54)	30.4 (5.82)	30.9 (6.79)
Median [Min, Max]	30.3 [17.0, 55.0]	29.5 [17.0, 54.9]	29.8 [17.0, 55.0]
**T2DM**	**Female (N=67,564)**	**Male (N=46,361)**	**Overall (N=113,925)**
T2DM = No	64,872 (96.0%)	43,043 (92.8%)	107,915 (94.7%)
T2DM = Yes	2,692 (4.0%)	3,318 (7.2%)	6,010 (5.3%)
Age (overall)			
Mean (SD)	58.5 (16.8)	62.2 (16.5)	60.0 (16.8)
Median [Min, Max]	61.0 [18.0, 95.0]	66.0 [20.0, 95.0]	63.0 [18.0, 95.0]
Age (T2DM = No)			
Mean (SD)	58.5 (17.0)	62.1 (16.9)	59.9 (17.0)

Median [Min, Max]	61.0 [20.0, 95.0]	66.0 [20.0, 95.0]	63.0 [20.0, 95.0]
Age (T2DM = Yes)			
Mean (SD)	59.5 (12.3)	63.3 (11.6)	61.6 (12.1)
Median [Min, Max]	60.0 [18.0, 92.0]	65.0 [20.0, 94.0]	63.0 [18.0, 94.0]
BMI			
Mean (SD)	28.7 (7.14)	28.8 (5.63)	28.8 (6.57)
Median [Min, Max]	27.1 [17.0, 55.0]	27.9 [17.0, 55.0]	27.5 [17.0, 55.0]
BMI (T2DM = No)			
Mean (SD)	28.4 (7.00)	28.6 (5.47)	28.5 (6.43)
Median [Min, Max]	26.9 [17.0, 55.0]	27.6 [17.0, 55.0]	27.3 [17.0, 55.0]
BMI (T2DM = Yes)			
Mean (SD)	35.1 (7.57)	32.6 (6.33)	33.7 (7.03)
Median [Min, Max]	34.6 [17.0, 55.0]	31.9 [17.3, 54.4]	32.9 [17.0, 55.0]

Sex was based on sex assigned at birth.
Abbreviations: AF: atrial fibrillation; CAD: coronary artery disease; CKD: chronic kidney disease; HF: heart failure; HTN: hypertension; T2DM: type 2 diabetes mellitus.

Association analysis with CVD-related outcomes in AoU

We extracted the variants identified as IVs in the exposure GWAS (MVP) using the Hail table from the AoU srWGS data. Variants with a minor allele frequency (MAF) <1% in the AoU dataset were excluded. The resulting Hail table was then converted into a PLINK file. To obtain individual-level genetic data for association analysis, we used the R package “BEDMatrix” to import the .bed file using the function “BEDMatrix”. Single-variant association analyses were performed for six binary outcomes using logistic regression, adjusting for age and 16 genetic principal components (PCs) in the BMI-unadjusted models. For BMI-adjusted analyses, the models were further adjusted for BMI, incorporating both linear and quadratic terms. The effect sizes of the variants (log(OR) with standard deviation) were extracted and used as summary statistics for the outcome GWAS. For the sex-combined analysis, we additionally adjusted for sex assigned at birth in the association models

Ethics statement

The All of Us research program was approved by a single IRB, the "All of Us IRB", which is charged with reviewing the protocol, informed consent, and other participant-facing materials for the All of Us Research Program. The IRB follows the regulations and guidance of the Office for Human Research Protections (https://www.hhs.gov/ohrp/index.html) for all studies, ensuring that the rights and welfare of research participants are overseen and protected uniformly. More information is provided online https://allofus.nih.gov/about/who-we-are/institutional-review-board-irb-of-all-of-us-research-program and in the All of Us design paper.

Acknowledgements

We gratefully acknowledge All of Us participants for their contributions and also thank the National Institutes of Health's All of Us Research Program for making available the participant data examined in this study. The All of Us Research Program is supported by the National Institutes of Health, Office of the Director: Regional Medical Centers: 1 OT2 OD026549; 1 OT2 OD026554; 1 OT2 OD026557; 1 OT2 OD026556; 1 OT2 OD026550; 1 OT2 OD 026552; 1 OT2 OD026553; 1 OT2 OD026548; 1 OT2 OD026551; 1 OT2 OD026555; IAA #: AOD 16037; Federally Qualified Health Centers: HHSN 263201600085U; Data and Research Center: 5 U2C OD023196; Biobank: 1 U24 OD023121; The Participant Center: U24 OD023176; Participant Technology Systems Center: 1 U24 OD023163; Communications and Engagement: 3 OT2 OD023205; 3 OT2 OD023206; and Community Partners: 1 OT2 OD025277; 3 OT2 OD025315; 1 OT2 OD025337; 1 OT2 OD025276. The All of Us Research Program would not be possible without the partnership of its participants.

Note S5: Summary of sleep-related GWASs from Million Veteran Program

We present Miami plots and quantile-quantile plot (Q-Q plot) to visualize the results of GWASs for five sleep-related traits (obstructive sleep apnea, insomnia, excessive daytime sleepiness, short sleep duration, and long sleep duration) using data from participants in the Million Veteran Program (MVP). Analyses were stratified by sex and conducted both with and without adjustment for BMI. The sample sizes for MVP's sex-specific sleep GWAS are reported in Table S9.

Table S9: Sample sizes of MVP sleep-related GWASs

	BMI-unadjusted		BMI-adjusted	
	**female**	**male**	**female**	**male**
**OSA**	29,795	380,473	29,325	372,890
**Insomnia**	29,793	380,450	29,323	372,867
**sleepiness**	15,346	212,574	15,033	206,861
**Short sleep**	15,262	210,589	14,951	204,913
**Long sleep**	15,262	210,589	14,951	204,913

Figure S28: Miami plot of sex-specific OSA GWAS without BMI adjustment

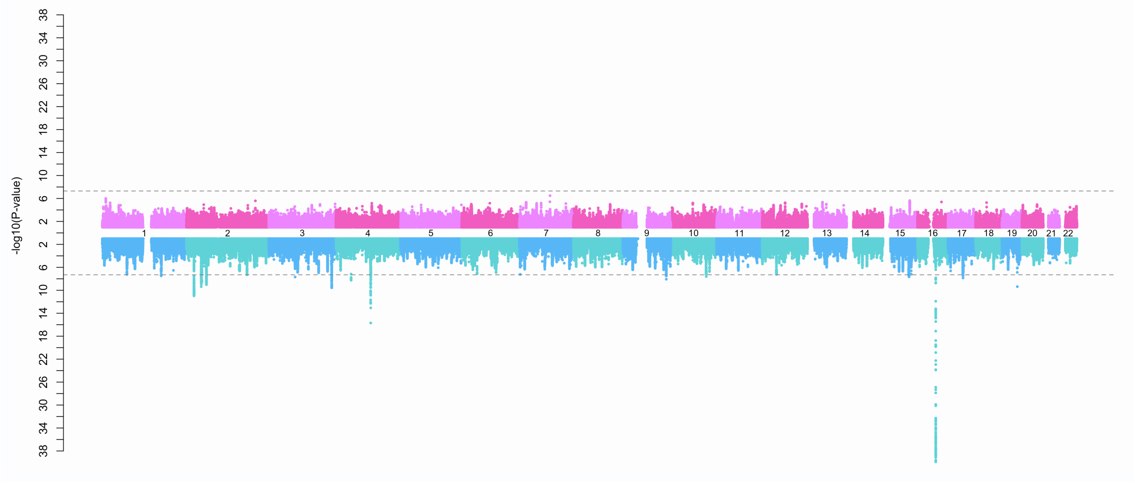

Female-specific results are shown at the top of the plot (pink color), and male-specific results are shown at the bottom (blue color). The y-axis provides the negative log p-values. The horizontal dashed line represents the genome-wide significance threshold (5×10^{-8}).
Abbreviations: OSA: obstructive sleep apnea; GWAS: genome-wide association study; BMI: body mass index.

Figure S29: Miami plot of sex-specific OSA GWAS with BMI adjustment

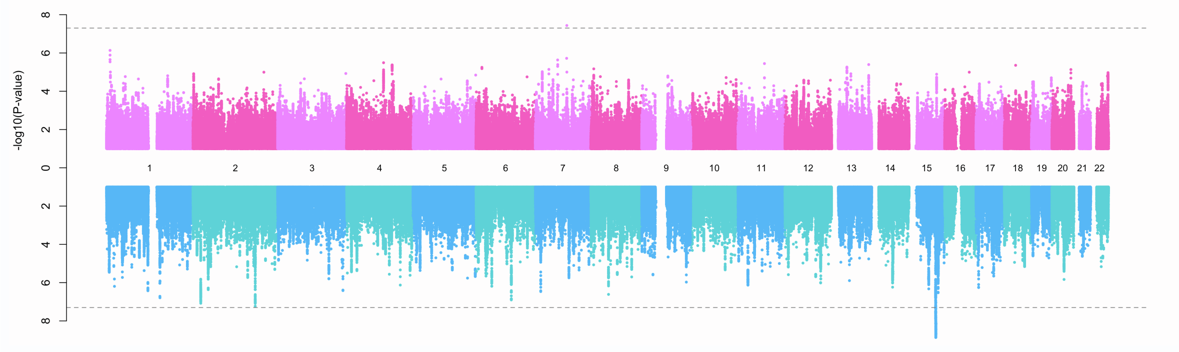

Female-specific results are shown at the top of the plot (pink color), and male-specific results are shown at the bottom (blue color). The y-axis provides the negative log p-values. The horizontal dashed line represents the genome-wide significance threshold (5×10^{-8}).
Abbreviations: OSA: obstructive sleep apnea; GWAS: genome-wide association study; BMI: body mass index.

Figure S30: Q-Q plots of sex-specific OSA GWASs

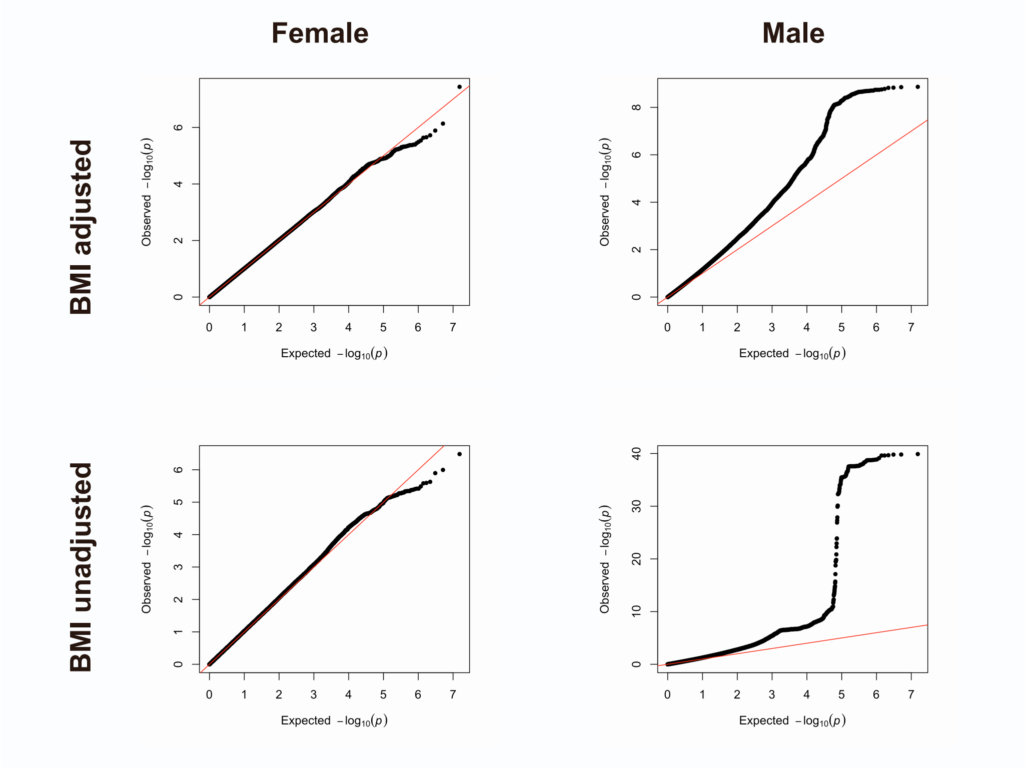

Female-specific results are shown on the left side of the plot, while male-specific results are shown on the right. GWAS results with BMI adjustment are shown at the top, and those without BMI adjustment are shown at the bottom.
Abbreviations: Q-Q plot: quantile-quantile plot; OSA: obstructive sleep apnea; GWAS, genome-wide association study; BMI, body mass index.

Figure S31: Miami plot of sex-specific insomnia GWAS without BMI adjustment

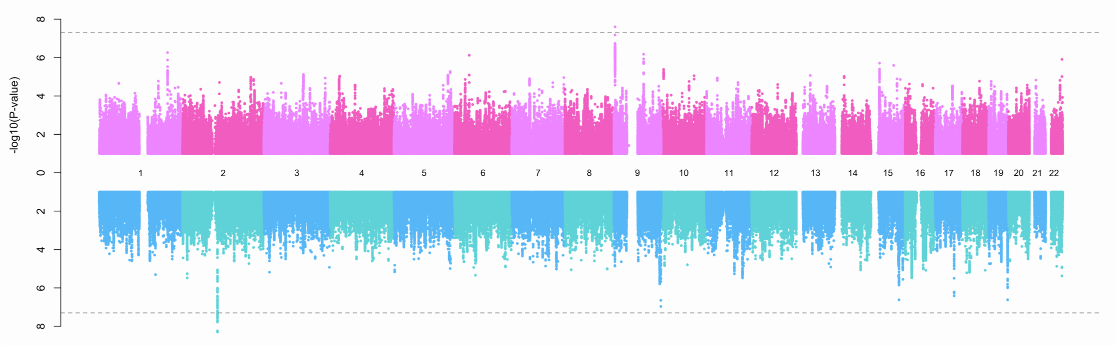

Female-specific results are shown at the top of the plot (pink color), and male-specific results are shown at the bottom (blue color). The y-axis provides the negative log p-values. The horizontal dashed line represents the genome-wide significance threshold (5×10^{-8}).

Abbreviations: GWAS: genome-wide association study; BMI: body mass index.

Figure S32: Miami plot of sex-specific insomnia GWAS with BMI adjustment

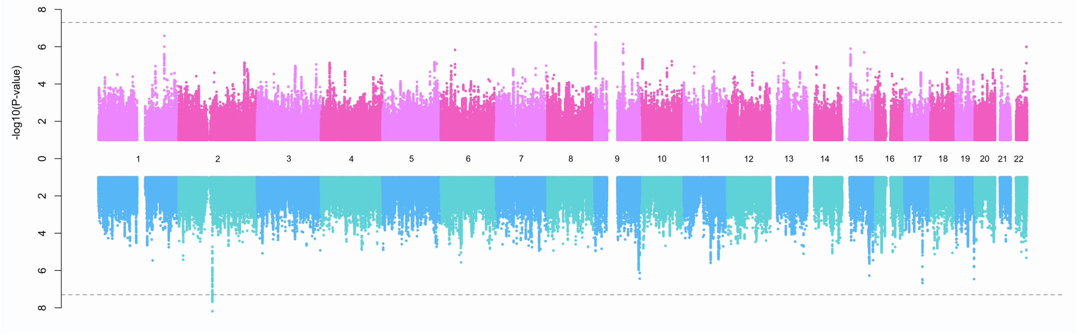

Female-specific results are shown at the top of the plot (pink color), and male-specific results are shown at the bottom (blue color). The y-axis provides the negative log p-values. The horizontal dashed line represents the genome-wide significance threshold (5×10^{-8}).

Abbreviations: GWAS: genome-wide association study; BMI: body mass index.

Figure S33: Q-Q plots of sex-specific insomnia GWASs

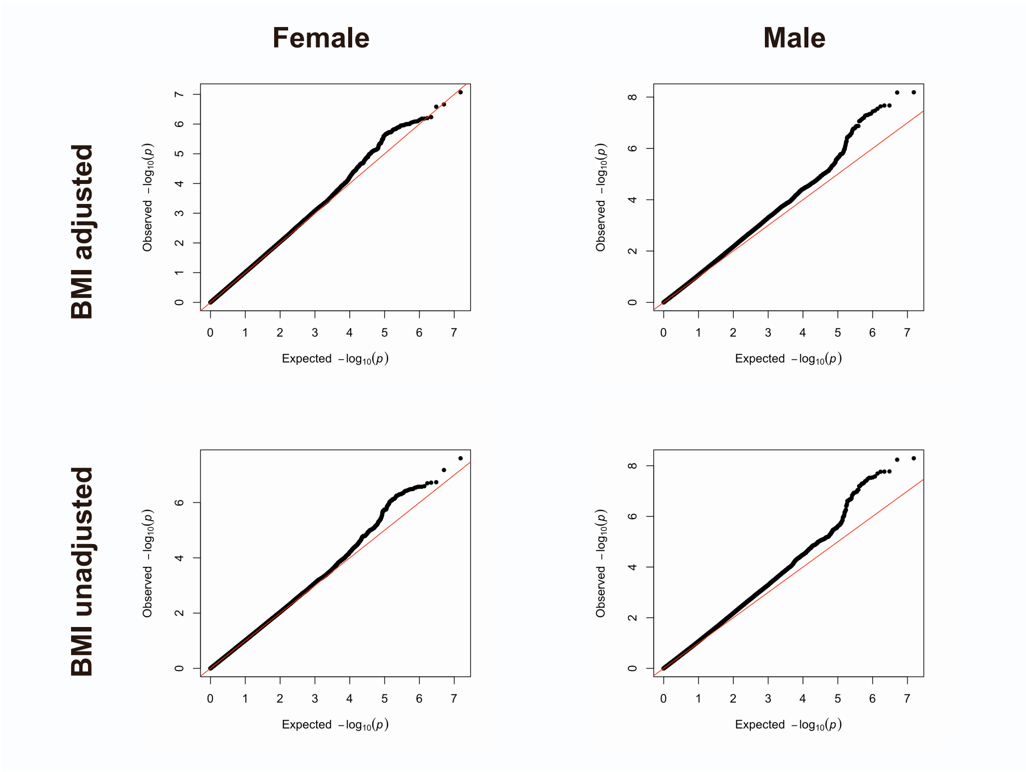

Female-specific results are shown on the left side of the plot, while male-specific results are shown on the right. GWAS results with BMI adjustment are shown at the top, and those without BMI adjustment are shown at the bottom.

Abbreviations: Q-Q plot: quantile-quantile plot; GWAS, genome-wide association study; BMI, body mass index.

Figure S34: Miami plot of sex-specific GWAS of excessive daytime sleepiness GWAS without BMI adjustment

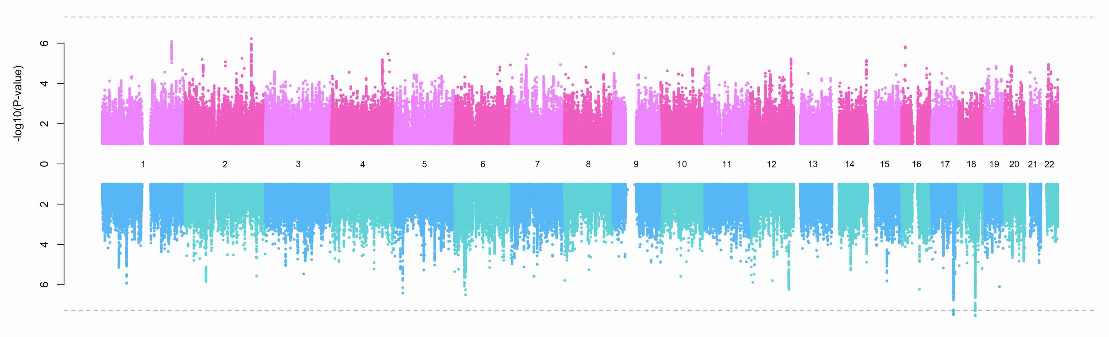

Female-specific results are shown at the top of the plot (pink color), and male-specific results are shown at the bottom (blue color). The y-axis provides the negative log p-values. The horizontal dashed line represents the genome-wide significance threshold (5×10^{-8}).
Abbreviations: GWAS: genome-wide association study; BMI: body mass index.

Figure S35: Miami plot of sex-specific GWAS of excessive daytime sleepiness GWAS with BMI adjustment

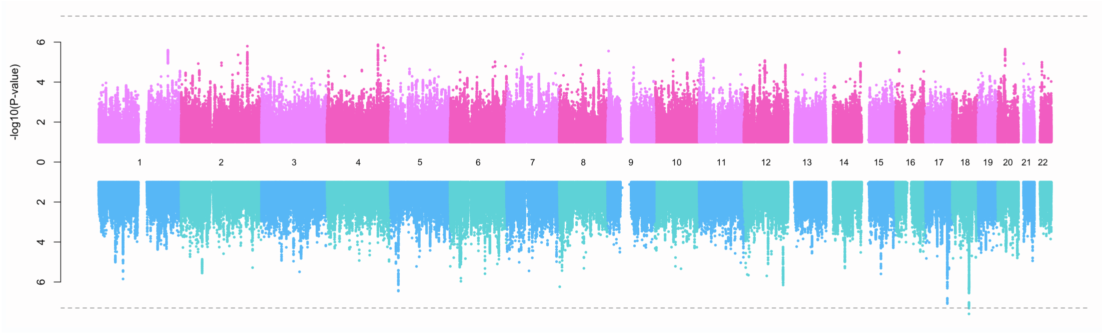

Female-specific results are shown at the top of the plot (pink color), and male-specific results are shown at the bottom (blue color). The y-axis provides the negative log p-values. The horizontal dashed line represents the genome-wide significance threshold (5×10^{-8}).
Abbreviations: GWAS: genome-wide association study; BMI: body mass index.

Figure S36: Q-Q plots of sex-specific excessive daytime sleepiness GWASs

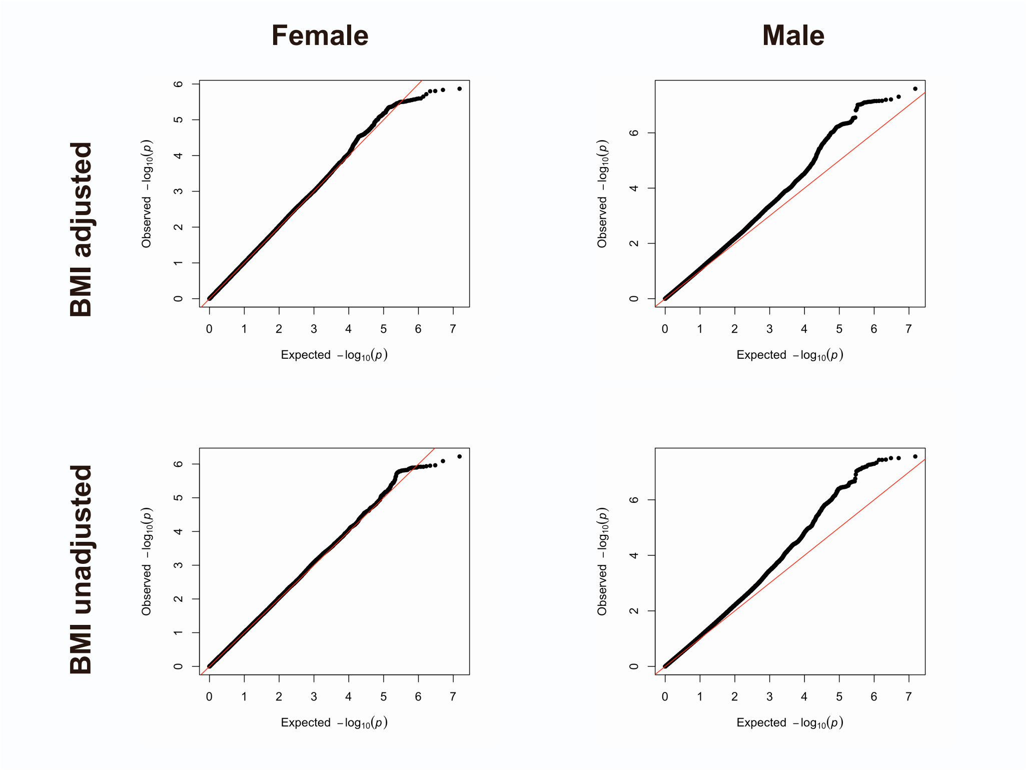

Female-specific results are shown on the left side of the plot, while male-specific results are shown on the right. GWAS results with BMI adjustment are shown at the top, and those without BMI adjustment are shown at the bottom.

Abbreviations: Q-Q plot: quantile-quantile plot; GWAS, genome-wide association study; BMI, body mass index.

Figure S37: Miami plot of sex-specific GWAS of short sleep duration, without BMI adjustment

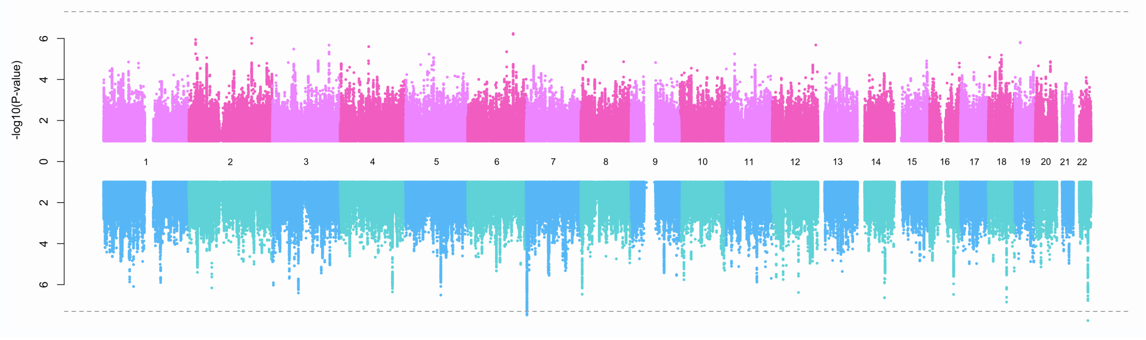

Female-specific results are shown at the top of the plot (pink color), and male-specific results are shown at the bottom (blue color). The y-axis provides the negative log p-values. The horizontal dashed line represents the genome-wide significance threshold (5×10^{-8}).
Abbreviations: GWAS: genome-wide association study; BMI: body mass index.

Figure S38: Miami plot of sex-specific GWAS of short sleep duration GWAS, BMI adjusted

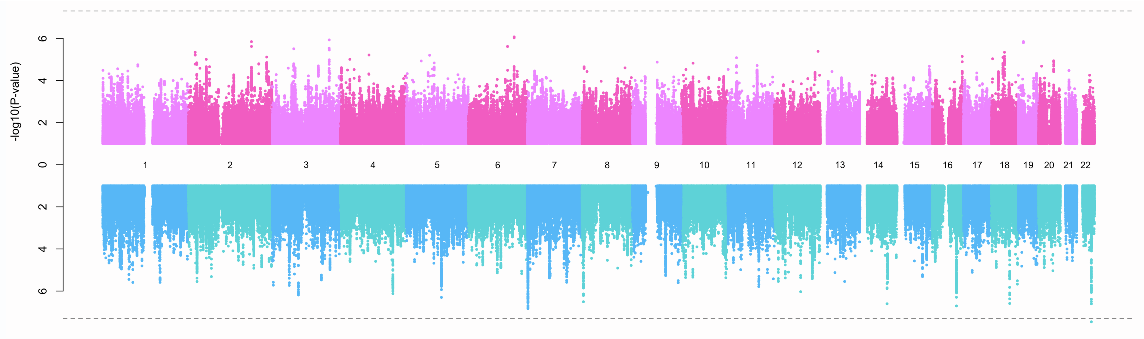

Female-specific results are shown at the top of the plot (pink color), and male-specific results are shown at the bottom (blue color). The y-axis provides the negative log p-values. The horizontal dashed line represents the genome-wide significance threshold (5×10^{-8}).
Abbreviations: GWAS: genome-wide association study; BMI: body mass index.

Figure S39: Q-Q plots of sex-specific short sleep duration GWASs

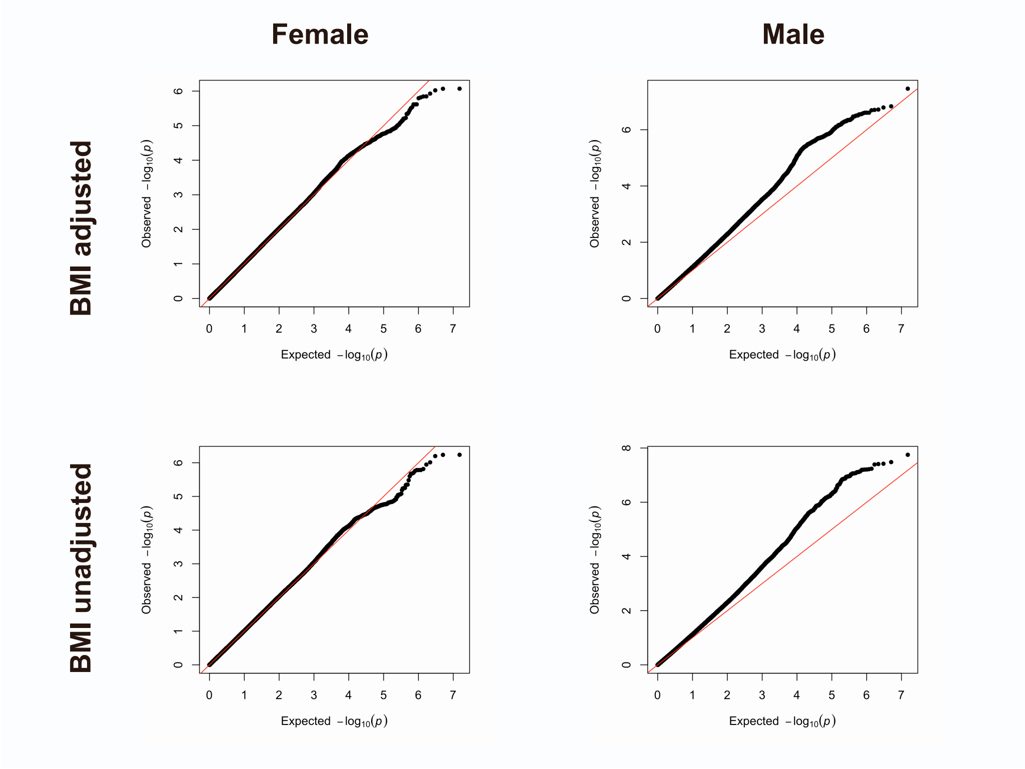

Female-specific results are shown on the left side of the plot, while male-specific results are shown on the right. GWAS results with BMI adjustment are shown at the top, and those without BMI adjustment are shown at the bottom.

Abbreviations: Q-Q plot: quantile-quantile plot; GWAS, genome-wide association study; BMI, body mass index.

Figure S40: Miami plot of sex-specific GWAS of long sleep duration, unadjusted to BMI

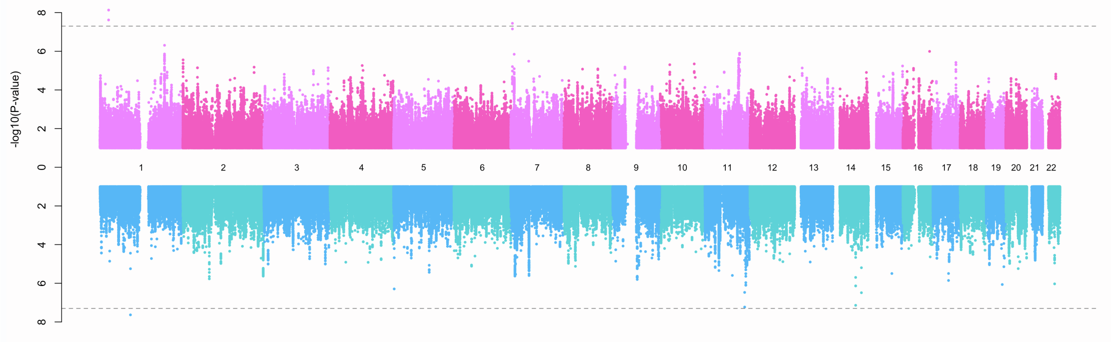

Female-specific results are shown at the top of the plot (pink color), and male-specific results are shown at the bottom (blue color). The y-axis provides the negative log p-values. The horizontal dashed line represents the genome-wide significance threshold (5×10^{-8}).
Abbreviations: GWAS: genome-wide association study; BMI: body mass index.

Figure S41: Miami plot of sex-specific GWAS of long sleep duration, BMI adjusted

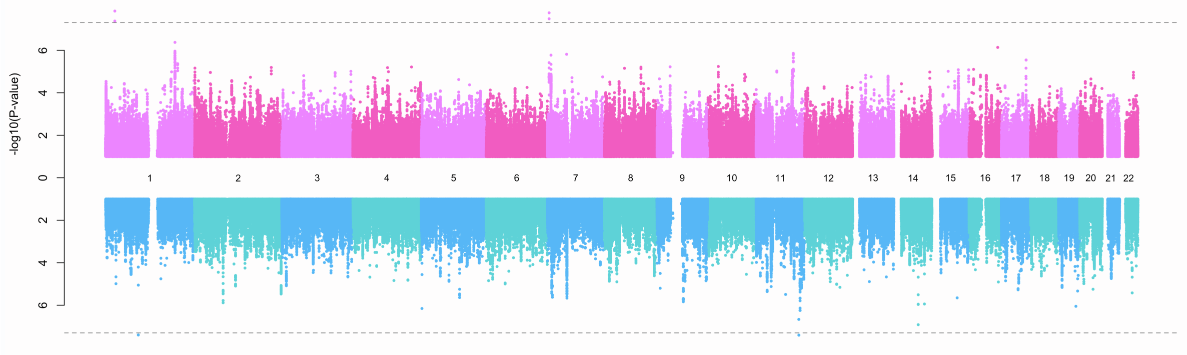

Female-specific results are shown at the top of the plot (pink color), and male-specific results are shown at the bottom (blue color). The y-axis provides the negative log p-values. The horizontal dashed line represents the genome-wide significance threshold (5×10^{-8}).
Abbreviations: GWAS: genome-wide association study; BMI: body mass index.

Figure S42: Q-Q plots of sex-specific long sleep duration GWASs

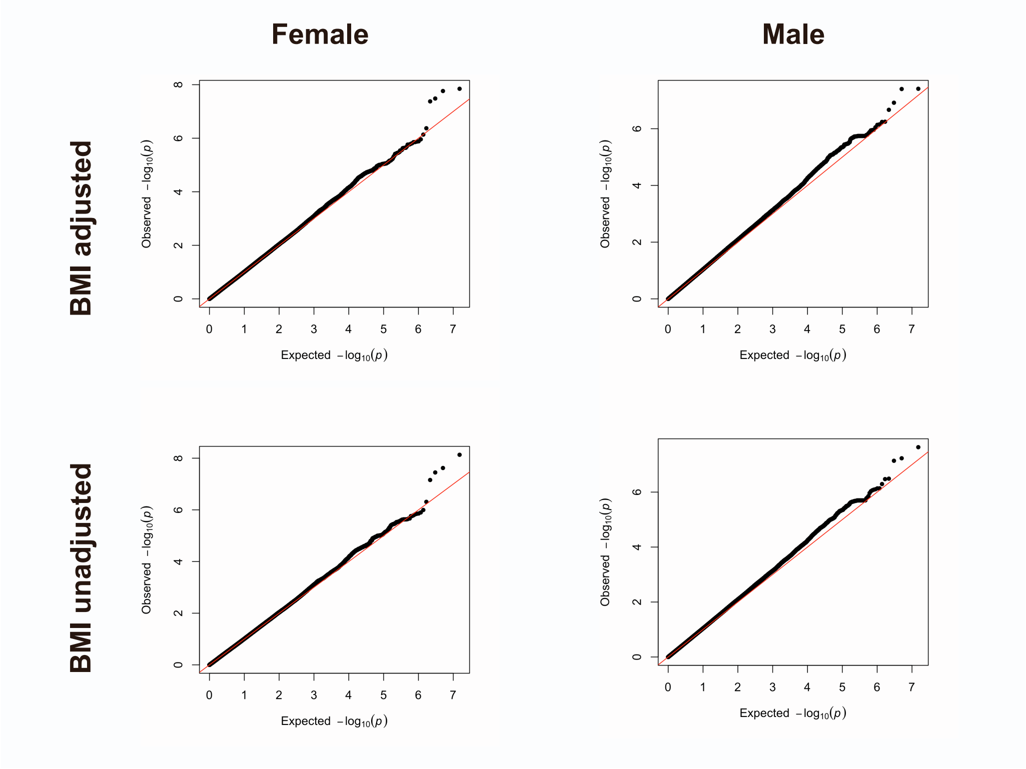

Female-specific results are shown on the left side of the plot, while male-specific results are shown on the right. GWAS results with BMI adjustment are shown at the top, and those without BMI adjustment are shown at the bottom.

Abbreviations: Q-Q plot: quantile-quantile plot; GWAS, genome-wide association study; BMI, body mass index.

Note S6: Million Veteran Program: Core Acknowledgements for Publications

MVP Ethics Statement

MVP received ethical/study protocol approval from the VA Central Institutional Review Board, and written informed consent was obtained for all participants.

MVP Program Office

- Sumitra Muralidhar, Ph.D., Program Director

  US Department of Veterans Affairs, 810 Vermont Avenue NW, Washington, DC 20420
- Jennifer Moser, Ph.D., Associate Director, Scientific Programs

  US Department of Veterans Affairs, 810 Vermont Avenue NW, Washington, DC 20420
- Jennifer E. Deen, B.S., Associate Director, Cohort & Public Relations

  US Department of Veterans Affairs, 810 Vermont Avenue NW, Washington, DC 20420

MVP Executive Committee

- Co-Chair: Philip S. Tsao, Ph.D.

  VA Palo Alto Health Care System, 3801 Miranda Avenue, Palo Alto, CA 94304
- Co-Chair: Sumitra Muralidhar, Ph.D.

  US Department of Veterans Affairs, 810 Vermont Avenue NW, Washington, DC 20420
- J. Michael Gaziano, M.D., M.P.H.

  VA Boston Healthcare System, 150 S. Huntington Avenue, Boston, MA 02130
- Elizabeth Hauser, Ph.D.

Durham VA Medical Center, 508 Fulton Street, Durham, NC 27705

- Amy Kilbourne, Ph.D., M.P.H.

  VA HSR&D, 2215 Fuller Road, Ann Arbor, MI 48105

- Michael Matheny, M.D., M.S., M.P.H.

  VA Tennessee Valley Healthcare System, 1310 24th Ave. South, Nashville, TN 37212

- Dave Oslin, M.D.

  Philadelphia VA Medical Center, 3900 Woodland Avenue, Philadelphia, PA 19104

- Deepak Voora, MD

  Durham VA Medical Center, 508 Fulton Street, Durham, NC 27705

MVP Co-Principal Investigators

- J. Michael Gaziano, M.D., M.P.H.

  VA Boston Healthcare System, 150 S. Huntington Avenue, Boston, MA 02130

- Philip S. Tsao, Ph.D.

  VA Palo Alto Health Care System, 3801 Miranda Avenue, Palo Alto, CA 94304

MVP Core Operations

- Jessica V. Brewer, M.P.H., Director, MVP Cohort Operations

  VA Boston Healthcare System, 150 S. Huntington Avenue, Boston, MA 02130

- Mary T. Brophy M.D., M.P.H., Director, VA Central Biorepository

  VA Boston Healthcare System, 150 S. Huntington Avenue, Boston, MA 02130

- Kelly Cho, M.P.H, Ph.D., Director, MVP Phenomics

VA Boston Healthcare System, 150 S. Huntington Avenue, Boston, MA 02130

- Lori Churby, B.S., Director, MVP Regulatory Affairs

  VA Palo Alto Health Care System, 3801 Miranda Avenue, Palo Alto, CA 94304

- Scott L. DuVall, Ph.D., Director, VA Informatics and Computing Infrastructure (VINCI)

  VA Salt Lake City Health Care System, 500 Foothill Drive, Salt Lake City, UT 84148

- Saiju Pyarajan Ph.D., Director, Data and Computational Sciences

  VA Boston Healthcare System, 150 S. Huntington Avenue, Boston, MA 02130

- Robert Ringer, Pharm.D., Director, VA Albuquerque Central Biorepository

  New Mexico VA Health Care System, 1501 San Pedro Drive SE, Albuquerque, NM 87108

- Luis E. Selva, Ph.D., Director, MVP Biorepository Coordination

  VA Boston Healthcare System, 150 S. Huntington Avenue, Boston, MA 02130

- Shahpoor (Alex) Shayan, M.S., Director, MVP PRE Informatics

  VA Boston Healthcare System, 150 S. Huntington Avenue, Boston, MA 02130

- Brady Stephens, M.S., Principal Investigator, MVP Information Center

  Canandaigua VA Medical Center, 400 Fort Hill Avenue, Canandaigua, NY 14424

- Stacey B. Whitbourne, Ph.D., Director, MVP Cohort Development and Management

  VA Boston Healthcare System, 150 S. Huntington Avenue, Boston, MA 02130

MVP Publications and Presentations Committee

- Co-Chair: Themistocles L. Assimes, M.D., Ph. D

  VA Palo Alto Health Care System, 3801 Miranda Avenue, Palo Alto, CA 94304

- Co-Chair: Adriana Hung, M.D.; M.P.H

VA Tennessee Valley Healthcare System, 1310 24th Ave. South, Nashville, TN 37212

- Co-Chair: Henry Kranzler, M.D.

  Philadelphia VA Medical Center, 3900 Woodland Avenue, Philadelphia, PA 19104
